# Comprehensive perspectives of metal and metal-free C–H activation approaches in pyrimidines and related heteroarenes: scope, mechanistic insights, and synthetic utility

**DOI:** 10.1039/d5ra07044a

**Published:** 2025-11-03

**Authors:** Amin Mirfarah, Soodabeh Hatami, Ali Shiri

**Affiliations:** a Department of Chemistry, Faculty of Science, Ferdowsi University of Mashhad Mashhad Iran alishiri@um.ac.ir

## Abstract

Pyrimidines, fundamental constituents of nucleic acids, pharmaceuticals, and agrochemicals, represent crucial scaffolds in organic synthesis owing to their electron-deficient aromatic character and multifaceted reactivity. This review meticulously assesses three distinct methodologies for the direct activation of C–H bonds in pyrimidines and associated heteroarenes bearing a pyrimidine moiety: transition-metal-catalyzed processes, metal-free techniques, and photochemical approaches. Metal-catalyzed strategies, utilizing catalysts such as palladium, nickel, or copper, facilitate the formation of C–C and C–N bonds with high regioselectivity, exemplified by the C(5)-arylation of 2-amino pyrimidines and the C(7)–H activation in pyrazolo[1,5-*a*]pyrimidines. Metal-free methodologies, which exploit Minisci-type radical reactions involving persulfates or phosphonium intermediates, provide sustainable functionalization pathways under mild reaction conditions. Photochemical methodologies, incorporating visible-light-driven photocatalysts such as eosin-Y or iridium complexes, facilitate radical-mediated arylations while offering environmental advantages. Through a comprehensive analysis of efficiency, regioselectivity, and scalability, this review also highlights significant progress in the synthesis of bioactive heterocycles, addresses pertinent challenges in green chemistry, and delineates avenues for future advancements in pyrimidine-centric therapeutics and innovative materials.

## Abstract 2

Pyrimidine C–H activation can be efficiently achieved through three complementary strategies: metal-catalyzed, metal-free, and photochemical approaches enabling direct functionalization and structural diversification of heteroaromatic frameworks.

## Graphical abstract

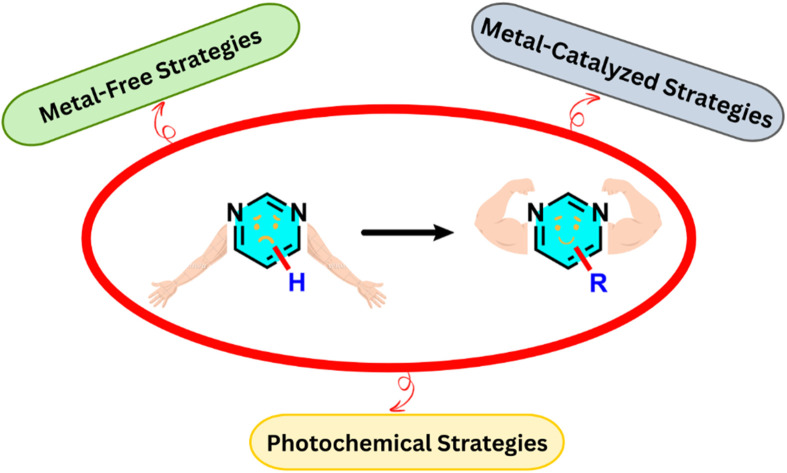

## 1 Introduction

Pyrimidines are heterocyclic compounds classified as diazines.^1^ These six-membered rings contain two nitrogen atoms and are recognized for their distinct molecular properties, including aromatic stability,^2,3^ hydrogen-bonding capability,^4–8^ selective reactivity,^8,9^ and electron-deficient nature due to electron-withdrawing nitrogen atoms.^1,5^ These characteristics establish pyrimidines as key components in the synthesis of bioactive pharmaceuticals^10^ and advanced agricultural materials.^7^ In 1888, Albrecht Kossel, a German physiologist and biochemist, successfully isolated theophylline (1,3-dimethylxanthine), a pyrimidine-containing compound, from tea leaves. A few years later, Neumann and Kossel further advanced this field by isolating and characterizing two fundamental pyrimidine derivatives, thymine and cytosine, from calf thymus tissues in 1893 and 1894, respectively.^4^ The term “PYRIMIDINE” was coined by Pinner, marking the inception of scientific studies on this cyclic system.^11^ Direct carbon–hydrogen (C–H) bond activation in these compounds, which enables precise molecular modification without requiring pre-functionalization steps, has emerged as one of the forefront areas in organic chemistry.^12^

**Fig. 1:**
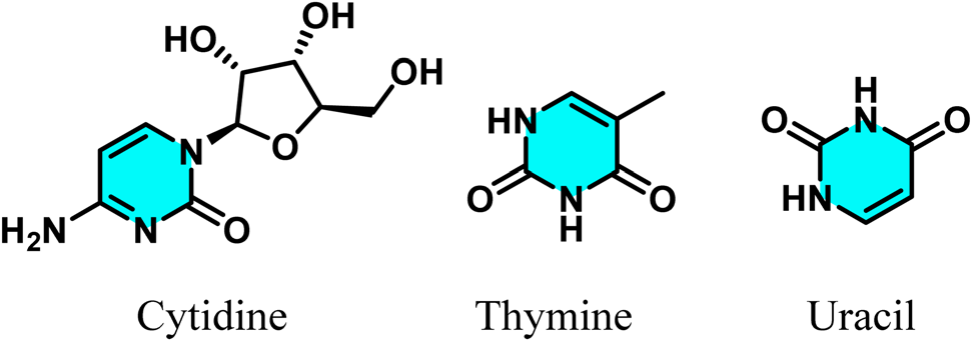
Chemical structures of cytidine, thymine, and uracil, essential pyrimidine components of nucleic acids.

In biological sciences, pyrimidines exhibit a wide range of properties. These molecules are integral to the structure of nucleic acids, including cytidine, thymine, and uracil, which are essential for forming the genetic code of cells (Fig. 1).^13^

**Fig. 2:**
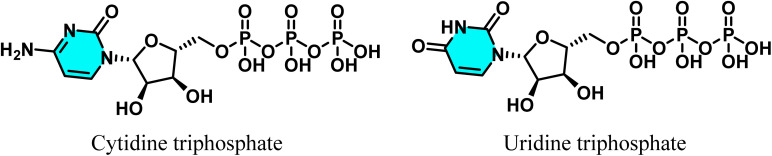
Chemical structures of cytidine triphosphate (CTP) and uridine triphosphate (UTP), key pyrimidine nucleotides in cellular metabolism.

Pyrimidines are critical for cell division, growth, and genetic functions. Deficiencies or disruptions in their biosynthetic pathways can impair cell growth or induce cell death, underscoring their functional significance and defining a crucial avenue for cancer therapeutics.^14^ In cellular metabolism, uridine-5′-triphosphate (UTP) and cytidine-5′-triphosphate (CTP), pyrimidine-derived nucleic acids, play key roles in carbohydrate and phospholipid metabolism, respectively (Fig. 2).^15,16^ Cells meet their pyrimidine requirements through two pathways. In the *de novo* pathway, pyrimidines are synthesized from simple precursors such as glycine, aspartate, and carbamoyl phosphate. Conversely, the salvage pathway recycles degraded bases and nucleotides for resynthesis.^17^ During catabolism, unlike purine rings, the pyrimidine ring can be fully degraded, ultimately yielding β-alanine or β-aminoisobutyrate, which contribute to the TCA cycle, amino acid metabolism, or fatty acid synthesis.^13^

**Fig. 3:**
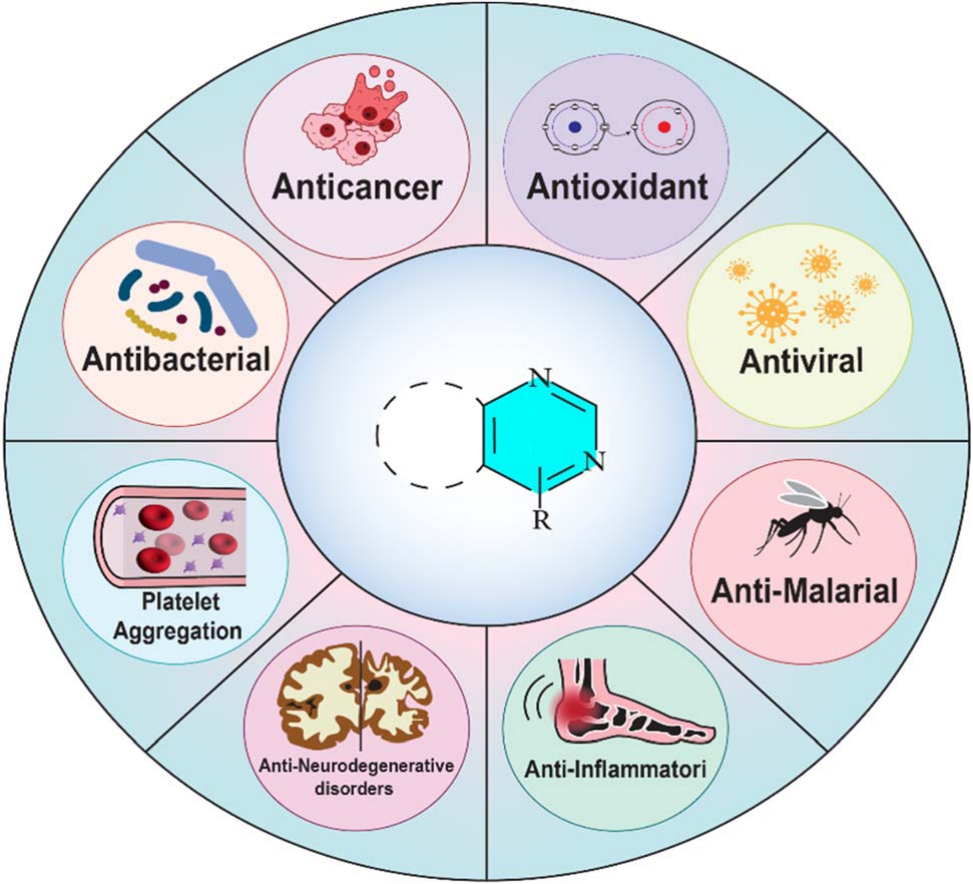
Biological activities of pyrimidine derivatives highlighting their therapeutic potential across various diseases.

Pyrimidine derivatives have been extensively utilized in the development of therapeutic agents, exhibiting a broad spectrum of pharmacological activities (Fig. 3), including antimicrobial, antiviral, anticancer, anti-inflammatory, and antioxidant properties.^8^ Furthermore, pyrimidines and their derivatives have garnered significant attention from researchers due to their diverse medicinal effects, such as antibacterial,^18^ antimalarial,^19^ antifungal,^20^ anti-Parkinson's, anti-Alzheimer's,^21^ antitubercular,^22^ anticonvulsant,^23^ antidepressant,^24^ anticoagulant,^25^ antidiabetic,^26^ analgesic,^27^ gastroprotective, kinase inhibitory,^28^ platelet aggregation inhibitory, antihypertensive, antiulcer,^29^ and antiplasmodial activities.^5^

**Fig. 4:**
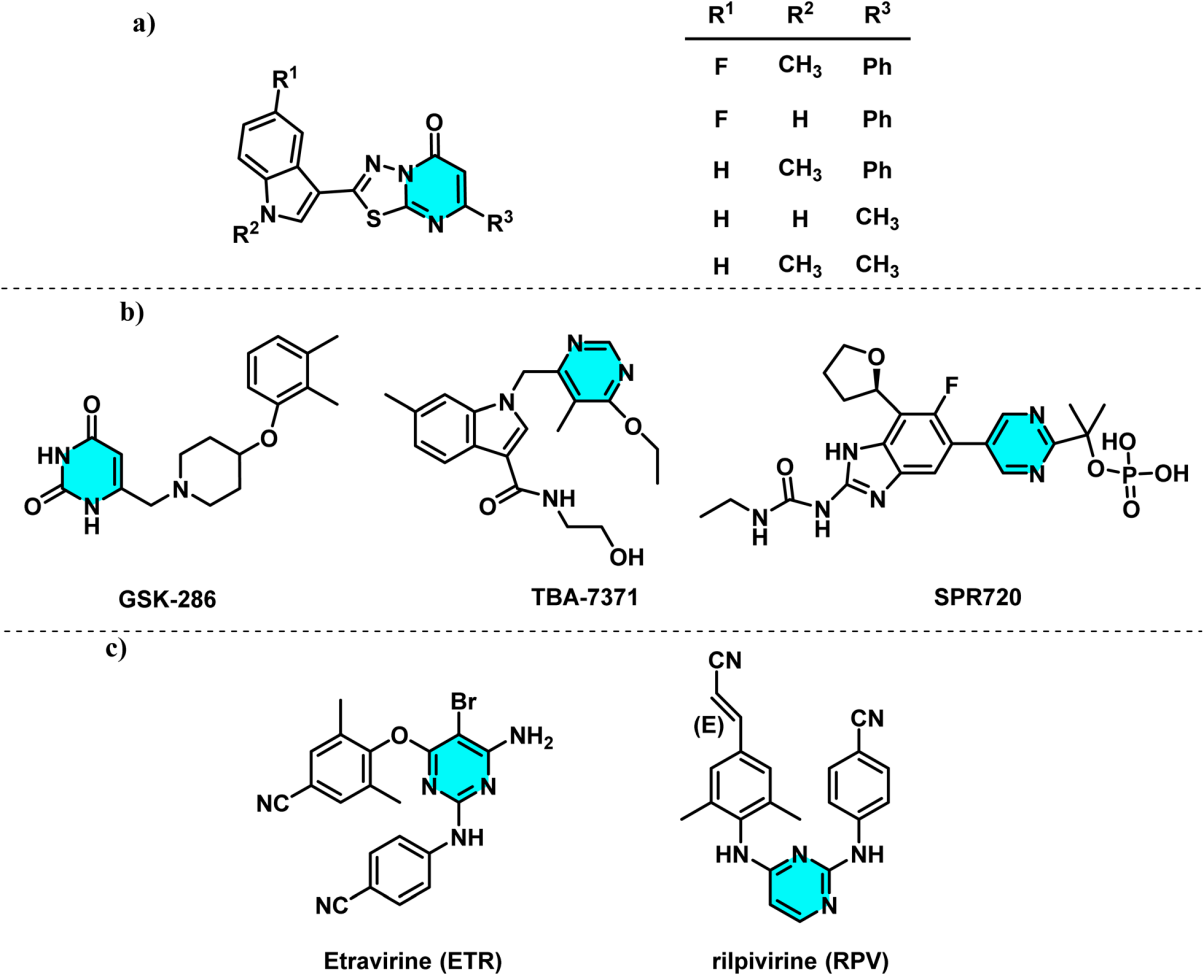
Representative pyrimidine-based compounds. (a) Thiadiazapyrimidinone derivatives with anti-biofilm activity. (b) Pyrimidine-containing clinical candidates for tuberculosis treatment. (c) Diarylpyrimidine (DAPY) NNRTIs used in HIV-1 therapy.

Carbone and co-workers developed twenty-two thiadiazapyrimidinone derivatives that effectively prevented biofilm formation and disrupted mature biofilms in both Gram-positive and Gram-negative pathogens (Fig. 4a).^30^ The azaindole derivative TBA-737, benzimidazole SPR720, and GSK 2556286, all containing a pyrimidine core, are currently under investigation in clinical trials for the treatment of *Mycobacterium tuberculosis* (Fig. 4b).^31–34^ Non-nucleoside reverse transcriptase inhibitors (NNRTIs) are critical for anti-HIV-1 therapy.^35^ Etravirine (ETR) and rilpivirine (RPV), second-generation NNRTIs, belong to the diarylpyrimidine (DAPY) family (Fig. 4c).^36^

**Fig. 5:**
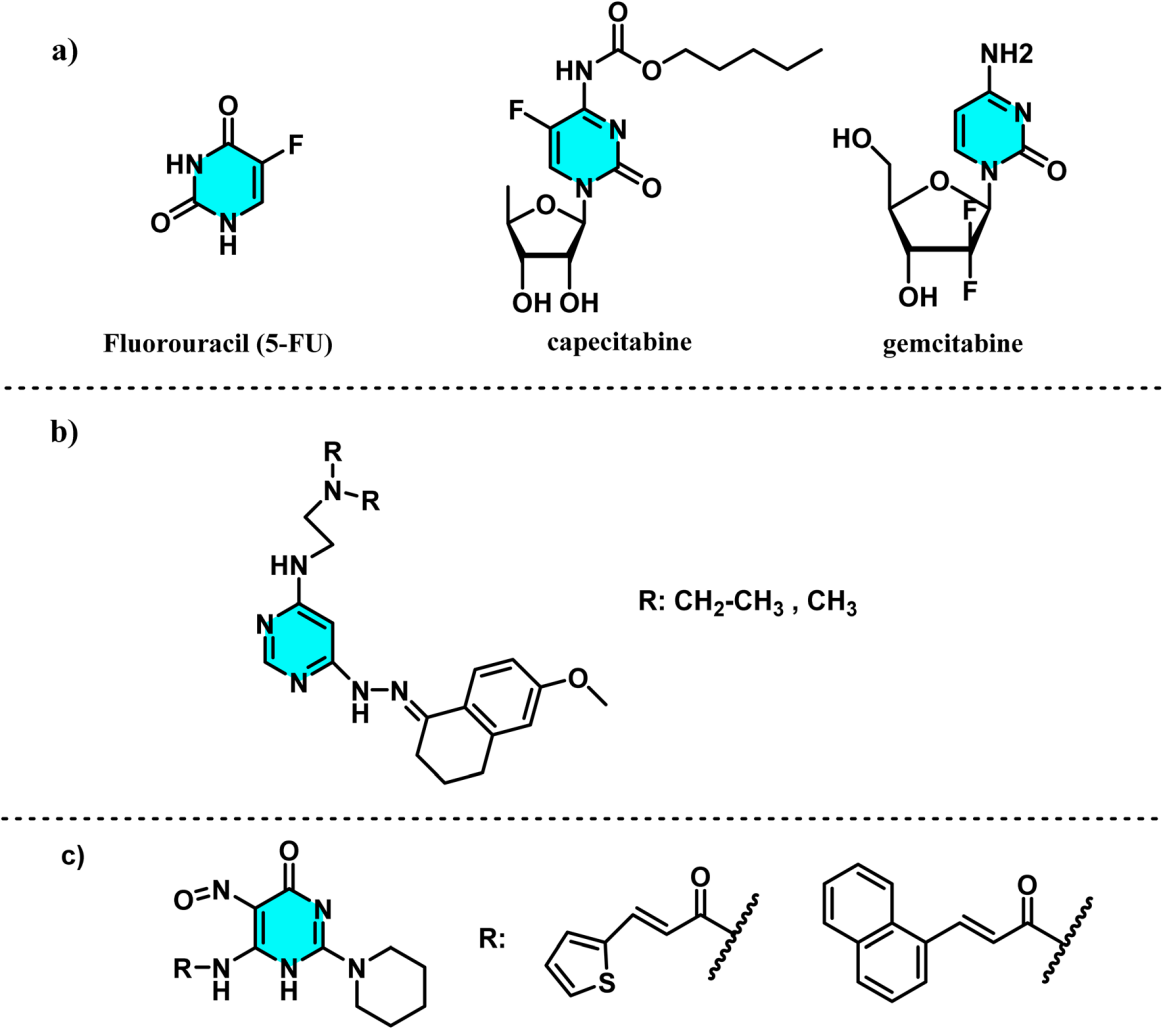
Structures of pyrimidine analog antimetabolites and derivatives with therapeutic potential. (a) Fluorouracil (5-FU), capecitabine, and gemcitabine, which mimic natural nucleotides and disrupt polymerase function, inhibiting cancer cell replication. (b) A pyrimidine derivative with antioxidant and anti-inflammatory properties, inhibiting COX-1 and COX-2 enzymes. (c) A novel pyrimidine acrylamide derivative exhibiting potent anti-lipoxygenase activity.

Additionally, these molecules show promising results in cancer treatment. As previously discussed, since nucleic acids are essential for replication, targeting pyrimidine biosynthesis pathways can be leveraged for the development of chemotherapeutic agents. Fluorouracil (5-FU), capecitabine, and gemcitabine are pyrimidine analog antimetabolites that mimic natural nucleotides but disrupt polymerase enzyme function, interfering with replication and proliferation in cancer cells (Fig. 5a).^37,38^ Beyond these roles, certain pyrimidine derivatives also exhibit antioxidant and anti-inflammatory properties. Tylińska and co-workers demonstrated that specific pyrimidine derivatives can inhibit COX-1 and COX-2, which are crucial for anti-inflammatory and anticancer therapies, while reducing Reactive Oxygen Species (ROS) levels in inflammatory cell lines (Fig. 5b).^39^ Saragatsis and co-workers designed six novel pyrimidine acrylamides, some of which emerged as promising candidates for developing new antioxidant derivatives with potent anti-lipoxygenase properties (Fig. 5c).^40^

Direct activation of carbon–hydrogen (C–H) bonds represents a transformative strategy in organic chemistry, enabling efficient and sustainable syntheses by converting inert bonds into functional ones.^41^ By eliminating the need for pre-functionalization, this approach reduces synthetic steps and minimizes the generation of hazardous waste, making it highly appealing for pharmaceutical and materials industries.^42^ However, the high bond dissociation energies of C–H bonds (≥90–110 kcal mol^−1^) and their ubiquity in organic molecules pose significant challenges in achieving chemo- and site-selectivity.^41,43^ C–H bond activation catalyzed by transition metals, involving inner-sphere cleavage of the C–H bond to form a carbon–metal bond, offers a sustainable and cost-effective method for synthesizing organic compounds. Consequently, this technique is recognized as an innovative and pivotal tool in organic chemistry.^44^ In this context, the terms “C–H activation” and “C–H functionalization” are commonly employed.^45^ For instance, C(sp^3^)–H activation in molecules such as 2-methylquinoline has facilitated the synthesis of complex heterocycles through cycloaddition reactions.^46^ The use of directing groups, such as Lewis-basic moieties, enhances selectivity by guiding the catalyst to the target bond.^43^ Recent advancements highlight the potential of this method for constructing complex heterocycles with minimal waste.^47^ Precise control of reaction conditions, such as temperature and solvent, is critical for optimizing yields.^48^ Broadly, C–H activation can be achieved through metal-catalyzed, metal-free, and photochemical approaches.^49^

Transition-metal-catalyzed methods, employing metals such as palladium,^50^ copper,^51^ rhodium,^52^ and nickel,^53^ achieve carbon–hydrogen (C–H) activation with high efficiency. Palladium excels in the arylation of pyrimidines with unparalleled selectivity, whereas cost-effective metals like copper and nickel offer compelling alternatives, particularly for large-scale industrial syntheses.^12^ For instance, nickel, owing to its abundance and low cost, has gained prominence in green synthesis approaches for pyrimidine-based pharmaceuticals.^54^ Environmental toxicity concerns have spurred the development of metal-free strategies.^55^ These methods utilize organic reagents, such as persulfates or hypervalent iodine, to enhance sustainability and are widely applied in the synthesis of bioactive molecules.^56,57^ Visible-light-mediated photochemical approaches, leveraging organic or metal-based photocatalysts and electron donor–acceptor (EDA) complexes, generate reactive radicals under mild conditions without heavy metal catalysts.^58^ Recent hybrid strategies combining metal catalysis with photochemistry have improved selectivity and efficiency, unlocking new applications in the synthesis of bioactive pyrimidines, although scalability for industrial production remains a challenge.^59^ Although several reviews have addressed aspects of C–H functionalization in heteroarenes, each exhibits clear limitations compared to the scope and depth of the present work. Verbitskiy *et al.*^60^ (2018) summarized both metal-catalyzed and metal-free functionalization of pyrimidines, yet their coverage predates major developments over the past eight years and entirely omits photochemical activation strategies. Gramage-Doria and Bruneau^61^ focused exclusively on transition-metal-catalyzed C–C bond formation in diazines, overlooking C–N and C–halogen coupling processes as well as metal-free and photochemical approaches. Docherty *et al.*^48^ examined C–H functionalization in complex molecular frameworks but without specific attention to pyrimidines, thereby missing the distinct reactivity features of this scaffold. Zheng and Schafer,^62^ restricted their discussion to C–H alkylation in saturated (aliphatic) N-heterocycles, excluding aromatic systems such as pyrimidines and disregarding the broader diversity of activation strategies.

In contrast, the present review delivers a comprehensive and up-to-date synthesis of three complementary activation modes metal-catalyzed, metal-free, and photochemical for C–H functionalization of pyrimidine-based heteroarenes. It systematically covers C–C, C–N, and C–halogen bond-forming reactions, while providing critical mechanistic analyses that dissect key pathways, intermediates, and reactivity trends across different catalytic platforms. By uniting methodological breadth with mechanistic depth, this review offers a distinctive, authoritative, and forward-looking resource that not only consolidates current knowledge up to 2025 but also maps out future directions for innovation in heterocyclic chemistry.

## 2 Metal-catalyzed strategies for direct C–H activation of pyrimidines and related heteroarenes

The activation of C–H bonds in heteroarenes using metal catalysts has emerged as a cornerstone in organic chemistry, owing to its high efficiency, selectivity, and capacity to synthesize complex molecules. This section surveys methodologies catalyzed by 12 transition metals, including palladium, copper, rhodium, and others, each contributing distinctively to the functionalization of pyrimidines and related heterocycles. This section of the review aims to provide a comprehensive perspective on the advancements and challenges of these approaches in synthesizing bioactive molecules.

### 2.1 Palladium-catalyzed C–H activation of pyrimidines

Palladium stands as one of the most widely employed catalysts for C–H activation in heteroarenes, particularly pyrimidines, owing to its exceptional efficiency and selectivity. Its ability to facilitate coupling reactions and operate under mild conditions has rendered it a prominent choice in the synthesis of bioactive compounds.

**Scheme 1:**
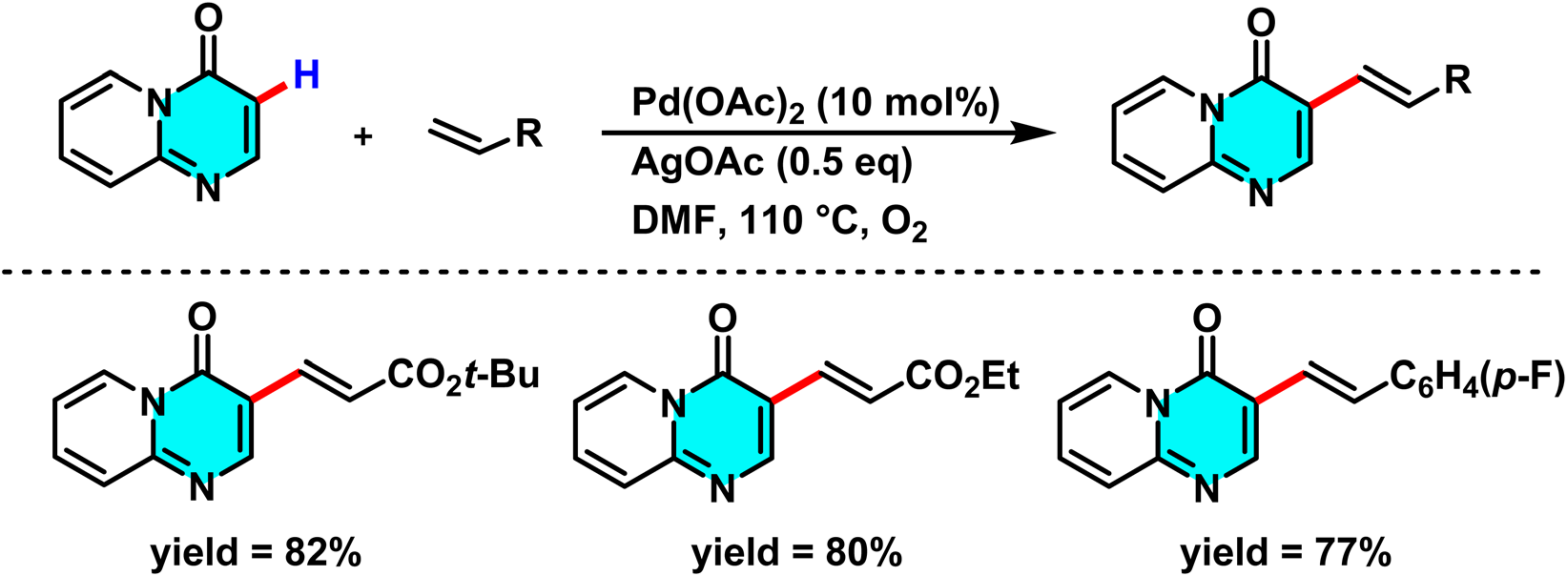
Pd(ii)-catalyzed alkenylation of 4*H*-pyrido[1,2-*a*]pyrimidin-4-ones.

Liu and co-workers reported a Pd(OAc)_2_-catalyzed method for selective C–H alkenylation at the C-3 position of 4*H*-pyrido[1,2-*a*]pyrimidin-4-ones using an AgOAc/O_2_ oxidizing system. The reaction proceeds directly with alkenes such as styrene, acrylates, and acrylamides without pre-derivatization. AgOAc and O_2_ regenerate the active Pd species, while DMF serves as an efficient solvent enhancing solubility and electron transfer (Scheme 1). This method achieves efficient alkenylation with high C-3 selectivity without requiring directing groups.

**Scheme 2:**
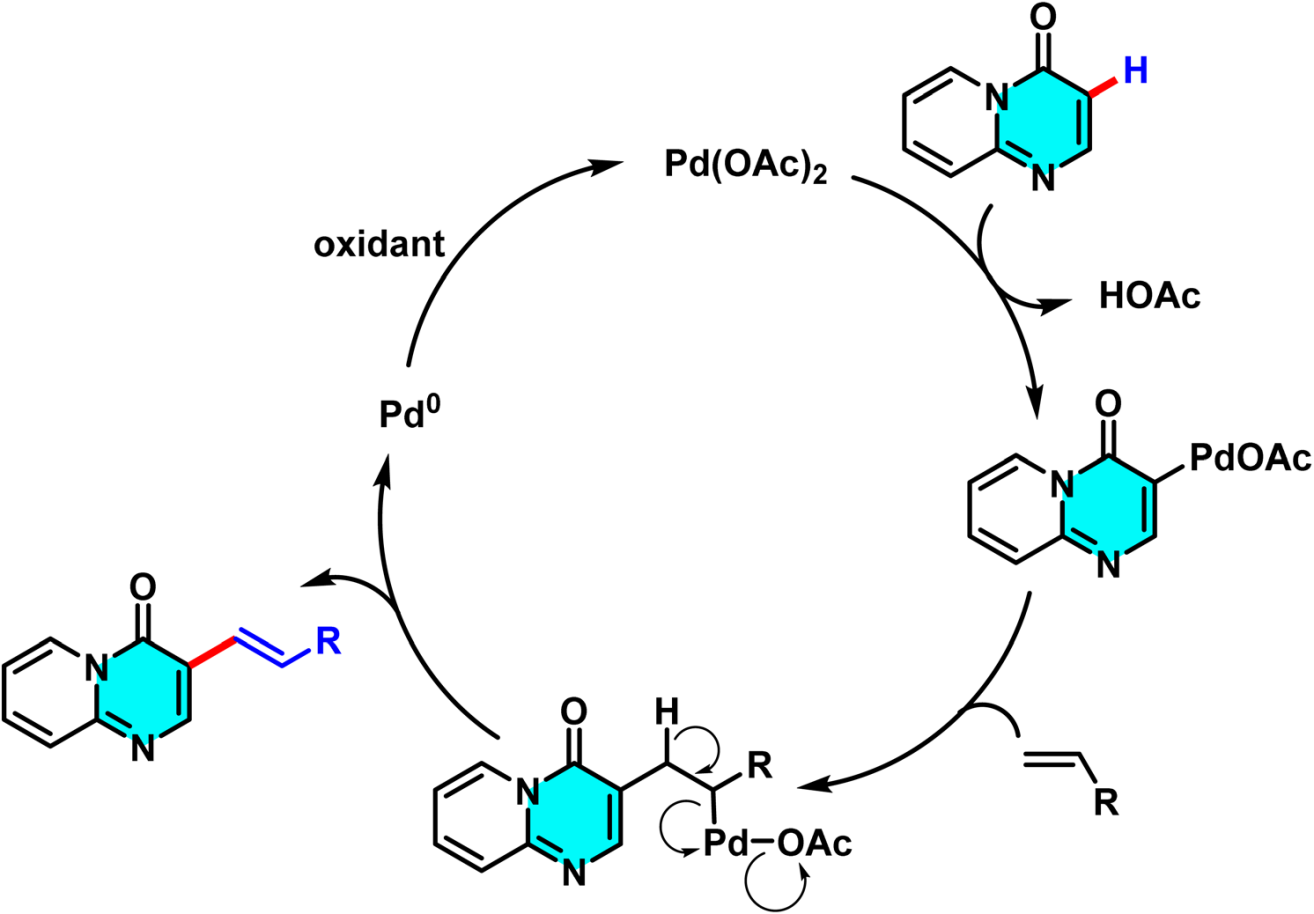
Plausible mechanism alkenylation of 4*H*-pyrido[1,2-*a*]pyrimidin-4-ones.

The reaction mechanism involves the formation of an intermediate palladium complex at the C-3 position, followed by the oxidative addition of the alkene to Pd(ii) and the transfer of the alkenyl group *via* a concerted metalation–dehydrogenation (CMD) process. Reductive elimination subsequently yields the final product and regenerates the palladium catalyst for further cycles (Scheme 2).^63^

**Scheme 3:**
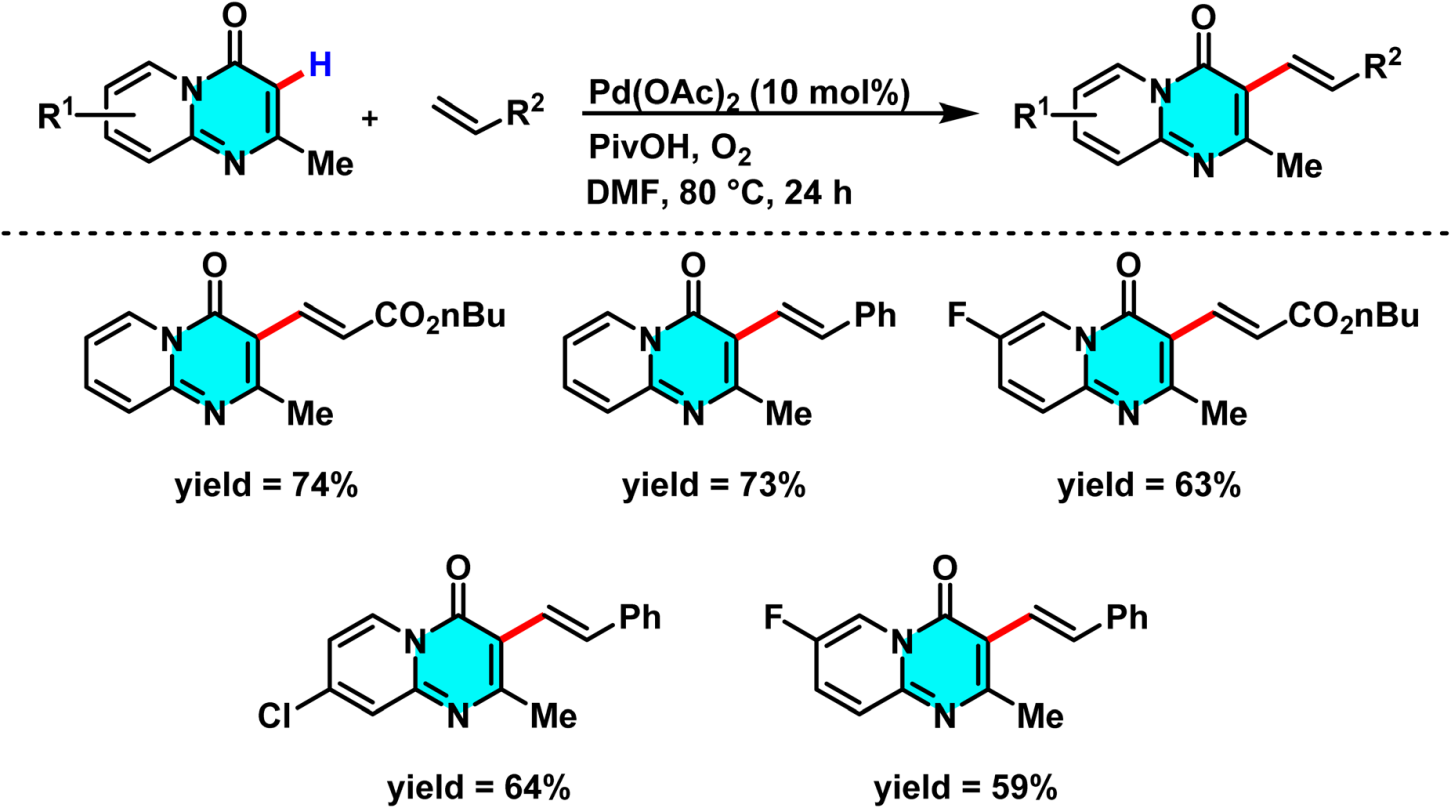
Selective C(3)–H alkenylation of 2-methyl-4*H*-pyrido[1,2-*a*]pyrimidin-4-ones.

In study conducted in 2015, Lv and co-workers reported a green and efficient method for the selective C–H alkenylation at the C-3 position of 2-methyl-4*H*-pyrido[1,2-*a*]pyrimidin-4-ones, catalyzed by palladium(ii) and utilizing molecular oxygen as the oxidant. Unlike traditional approaches that require pre-derivatization or employ toxic metal-based oxidants such as AgOAc and CuI, this method uses oxygen as a sustainable and environmentally benign oxidant. The use of oxygen sustains the palladium catalytic cycle and significantly enhances reaction yields. The catalyst Pd(OAc)_2_ plays a critical role by forming an intermediate palladium complex and selectively metalating the C(3)–H bond, while the additive pivalic acid (PivOH) reduces the activation energy, boosting the yield from 23% to 80%. Conducted in DMF solvent, which enhances reactant solubility and stabilizes palladium complexes, this reaction enables the direct coupling of pyrido[1,2-*a*]pyrimidines with alkenes such as styrene, acrylate esters, and acrylamides, achieving high C-3 selectivity (Scheme 3).

**Scheme 4:**
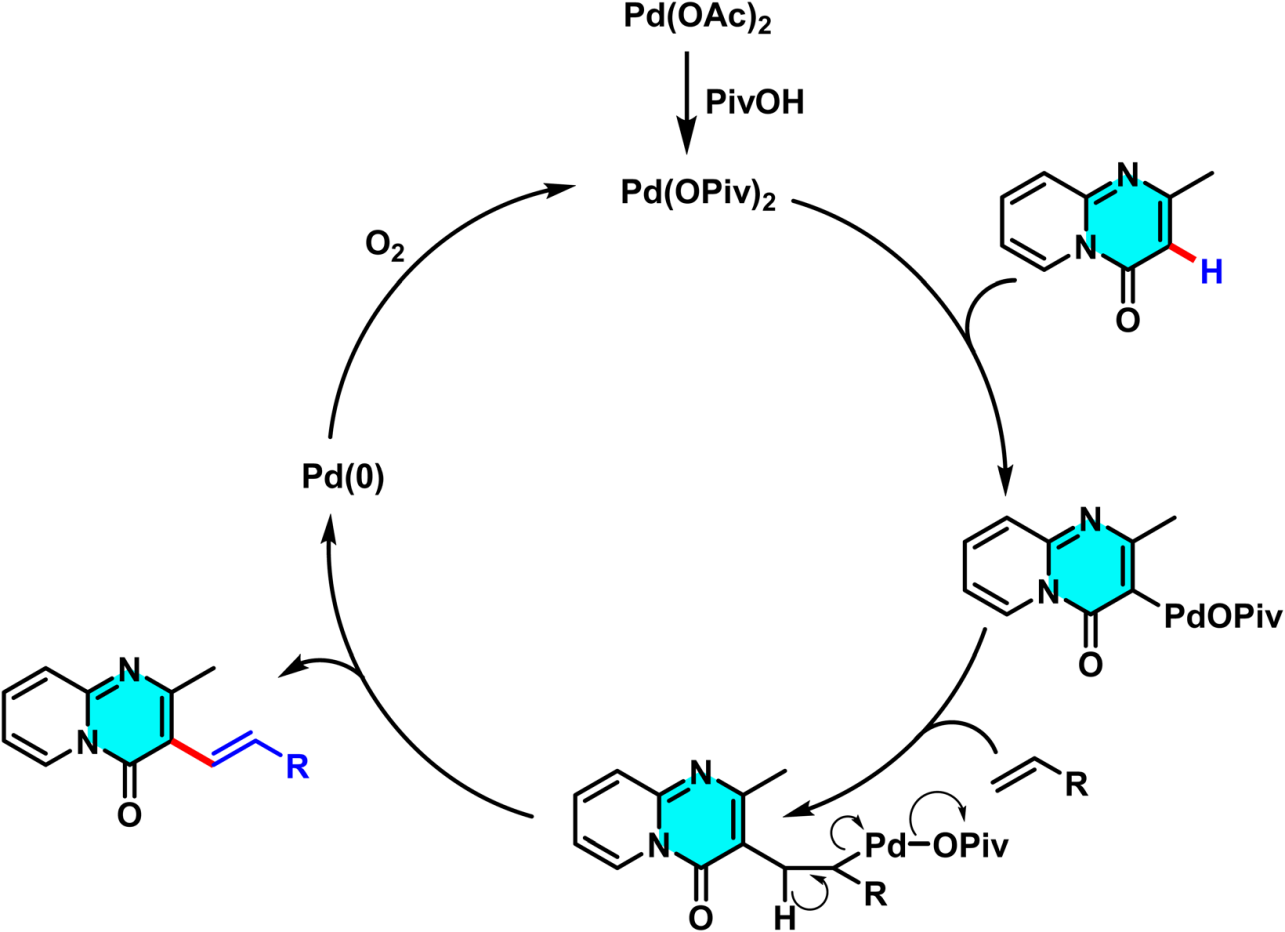
Proposed mechanism for alkenylation of 2-methyl-4*H*-pyrido[1,2-*a*]pyrimidin-4-ones.

According to the proposed mechanism, palladium first coordinates at the C-3 position, where it undergoes oxidative addition with the alkene, followed by CMD-facilitated alkenyl transfer. Reductive elimination subsequently yields the final product and regenerates the palladium catalyst for further cycles (Scheme 4).^64^

**Scheme 5:**
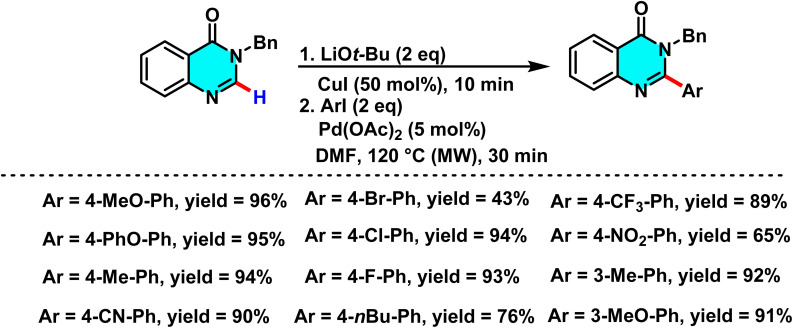
A ligand-free method for the direct C–H arylation of quinazolin-4-ones using Pd(ii) as catalyst and Cu(i) as a cocatalyst.

In 2015, Laclef and co-workers reported a ligand-free Pd(OAc)_2_/CuI-catalyzed C–H arylation of quinazolin-4-ones. Pd(ii) activates the C(2)–H bond *via* metalation, while CuI enhances catalyst stability and electron transfer. *t*-BuOLi assists proton abstraction, promoting formation of the active Pd species. Conducting the reaction in DMF under microwave irradiation greatly accelerates the process, completing it within 30 minutes (Scheme 5).

**Scheme 6:**
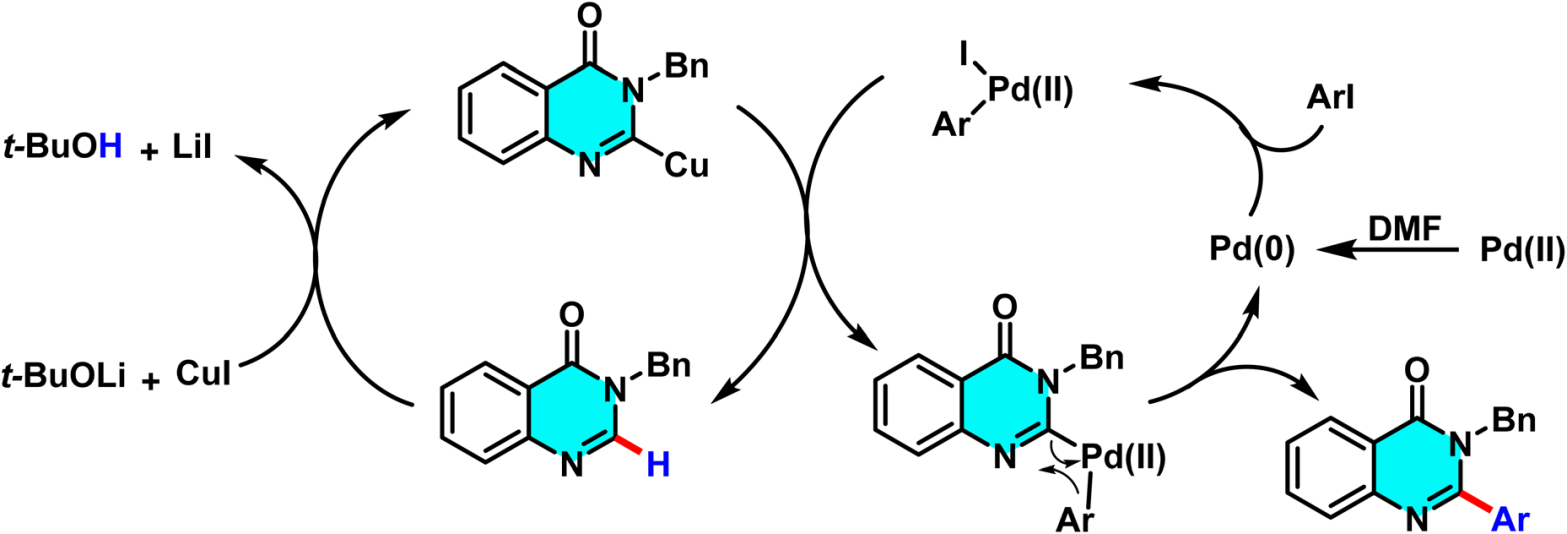
Plausible pathway illustrating the selective C-2 arylation of quinazolin-4-ones derivative.

The reaction mechanism involves the formation of an intermediate palladium complex *via* C(2)–H metalation, followed by the oxidative addition of an aryl iodide to generate a Pd(ii)-aryl species. Subsequently, the aryl group is transferred to the C-2 position through a CMD process, forming a new C–C bond. Reductive elimination then releases the final product while regenerating the palladium catalyst for the catalytic cycle (Scheme 6). Experimental results demonstrate that this method is compatible with a broad range of aryl iodides, including both electron-withdrawing and electron-donating variants, and is scalable for larger reactions. By avoiding ligands and minimizing synthetic steps, this approach offers a rapid and efficient strategy for modifying heterocyclic scaffolds such as quinazolin-4-ones in bioactive molecule development.^65^

**Scheme 7:**
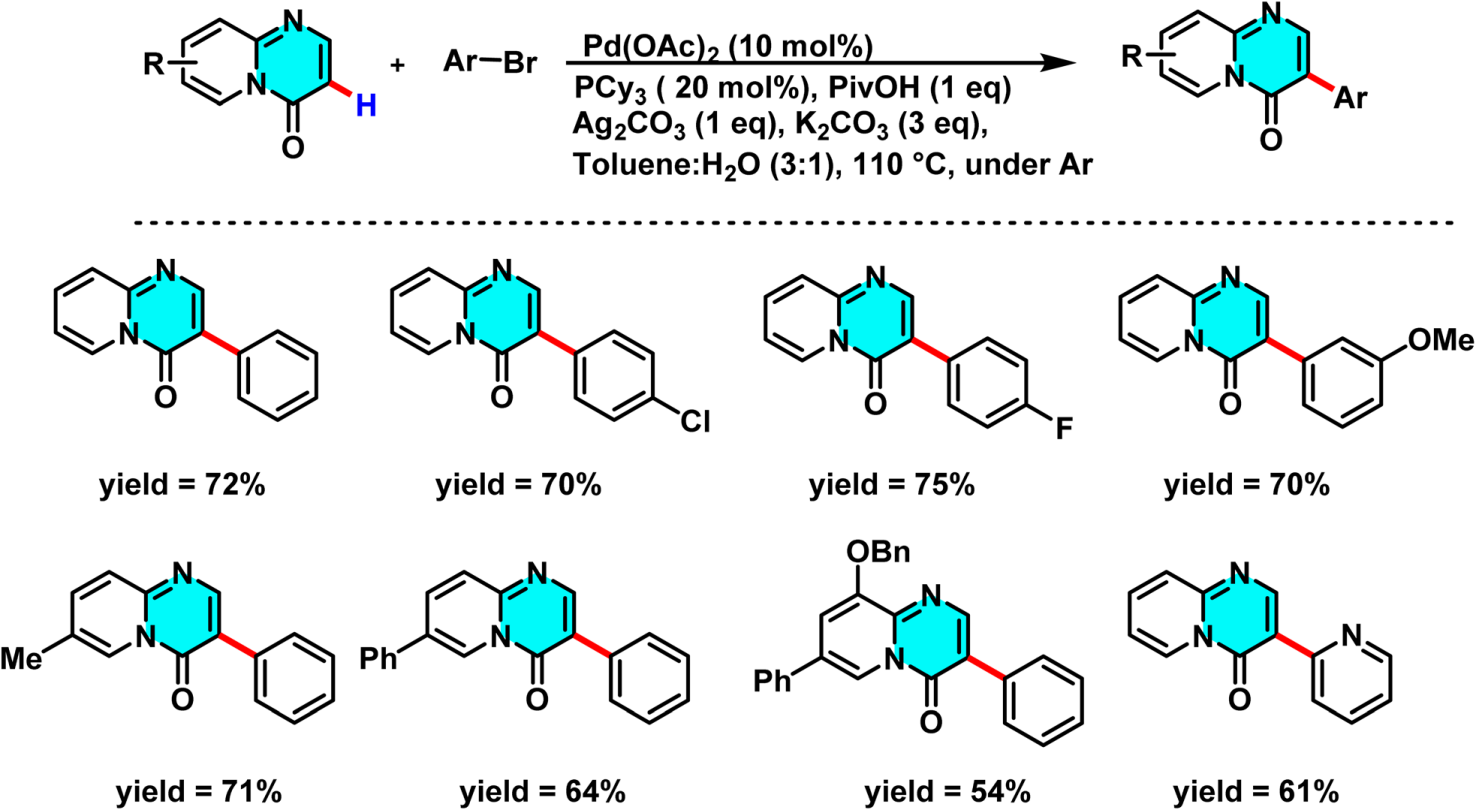
Pd(ii)-catalyzed synthesis of 3-aryl-pyrido[1,2-*a*]pyrimidin-4-ones in the presence of Ag(i) as a co-catalyst.

Studies in 2015 introduced a new method to synthesize 3-aryl-pyrido[1,2-*a*]pyrimidin-4-ones, compounds with high pharmaceutical value. This approach utilizes palladium (Pd) and silver (Ag(i)) catalysts to selectively activate the C(3)–H bond of pyrido[1,2-*a*]pyrimidin-4-ones with bromo/iodoarenes. The reaction is performed in a water-toluene mixture, offering advantages such as good yields (up to 75%), compatibility with diverse functional groups (*e.g.*, formyl and acetyl), and avoidance of common byproducts (Scheme 7).

**Scheme 8:**
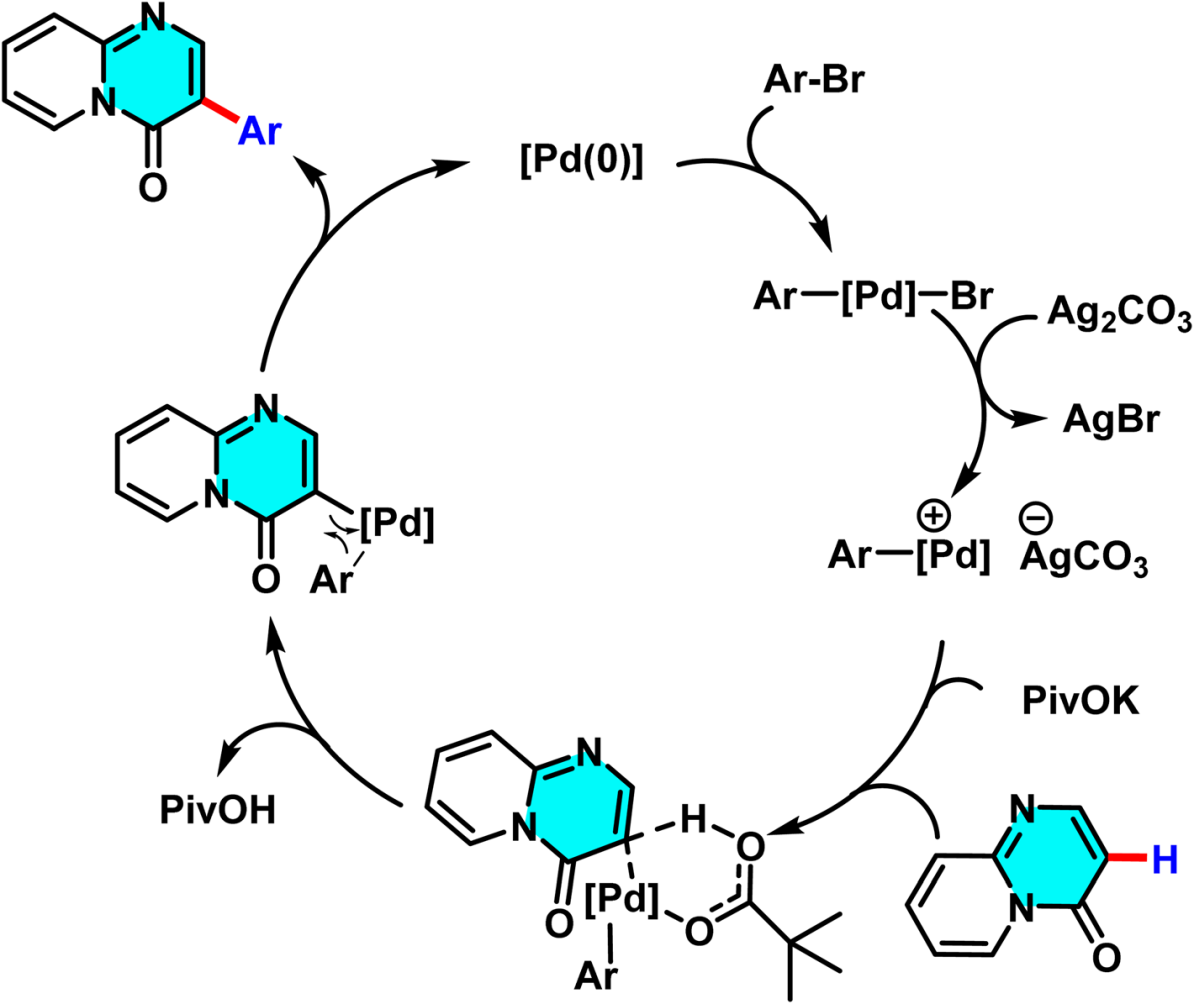
Proposed mechanism for the synthesis of 3-aryl-pyrido[1,2-*a*]pyrimidin-4-ones.

The reaction mechanism comprises several key steps. Initially, the bromoarene undergoes oxidative addition with Pd(0), forming an aryl-palladium intermediate (Ar–Pd(ii)–Br). Subsequently, silver carbonate (Ag_2_CO_3_) acts as a halogen scavenger, removing bromide to generate a cationic aryl-palladium species (Ar–Pd(ii)^+^) a critical and unconventional step confirmed by electrospray ionization mass spectrometry (ESI-MS). This cationic species then reacts with pyrido[1,2-*a*]pyrimidin-4-one in the presence of pivalate (PivO^−^), activating the C(3)–H bond *via* a CMD route. This process forms a transition state in which palladium binds to C-3 while the proton is abstracted. Finally, reductive elimination yields the arylated product and regenerates Pd(0) (Scheme 8).^66^

**Scheme 9:**
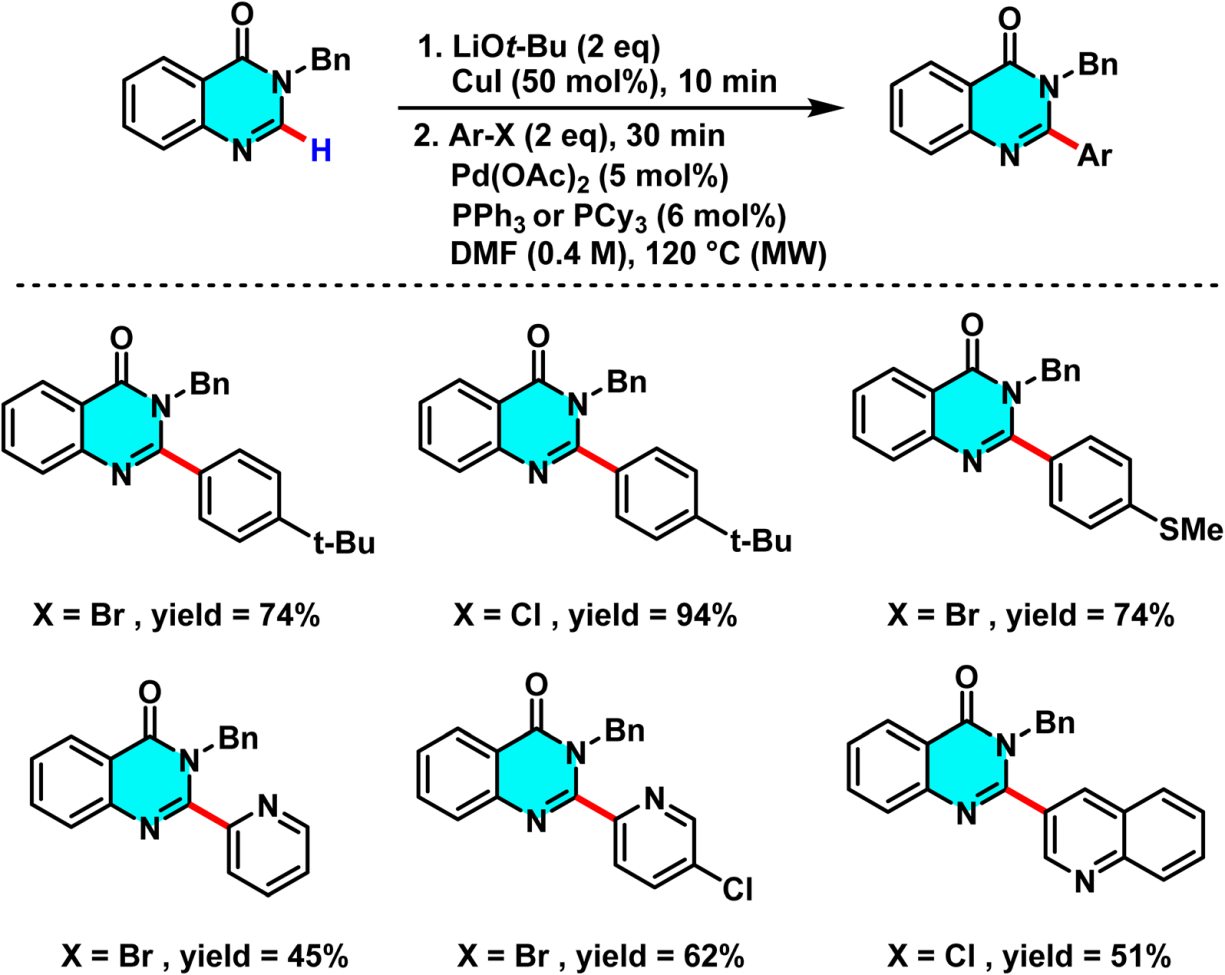
Selective arylation of the C-2 position of N-3-substituted quinazolin-4(3*H*)-ones using (hetero)aryl halides under microwave irradiation.

**Scheme 10:**
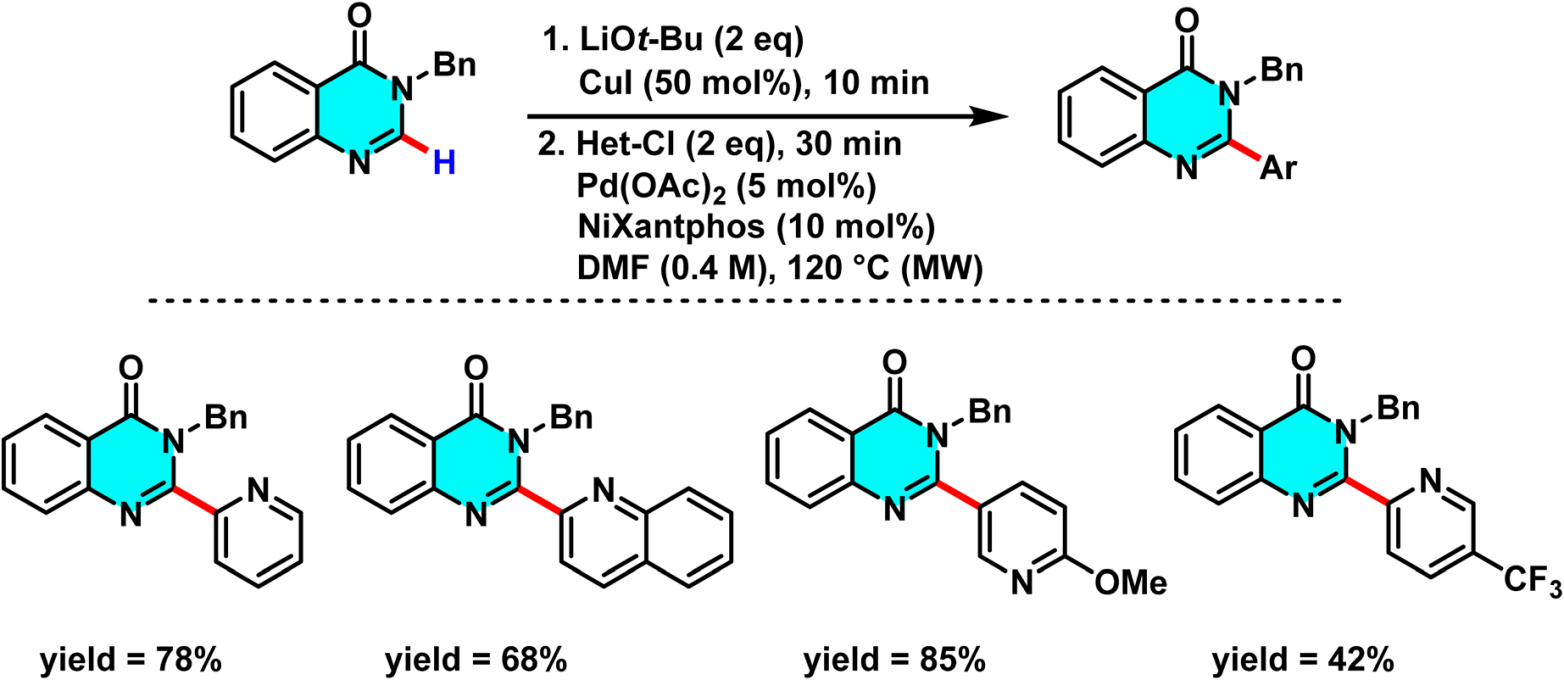
Selective Pd(ii)-catalyzed C–H arylation of quinazolin-4(3*H*)-ones with (hetero)aryl halides under microwave irradiation, using NiXantphos as the ligand.

In 2015, Godeau and co-workers introduced a novel method for the direct C–H activation of heterocycles, enabling the selective arylation of the C-2 position of N-3-substituted quinazolin-4(3*H*)-ones using (hetero)aryl halides under microwave irradiation. The initial protocol (Scheme 9) involved the use of aryl bromides and chlorides as electrophilic coupling partners, and employed a bimetallic catalytic system comprising Pd(OAc)_2_ (5 mol%) as the primary catalyst, CuI (50 mol%) as cocatalyst, *t*-BuOLi as base, and PPh_3_ (for bromides) or NiXantphos (for chlorides) as ligand. The palladium catalyst undergoes oxidative addition to the C–X bond of the aryl halide, while copper facilitates substrate activation through coordination with the quinazoline core, ensuring positional selectivity. In a subsequent extension of this methodology, the authors demonstrated its applicability to heteroaryl bromides under similar conditions, with NiXantphos acting as an effective ligand (Scheme 10). The transformation proved compatible with a wide range of (hetero)aryl halides, including iodides, bromides, and chlorides. It proceeds efficiently in DMF at 120 °C, affording high yields within 30 minutes.

**Scheme 11:**
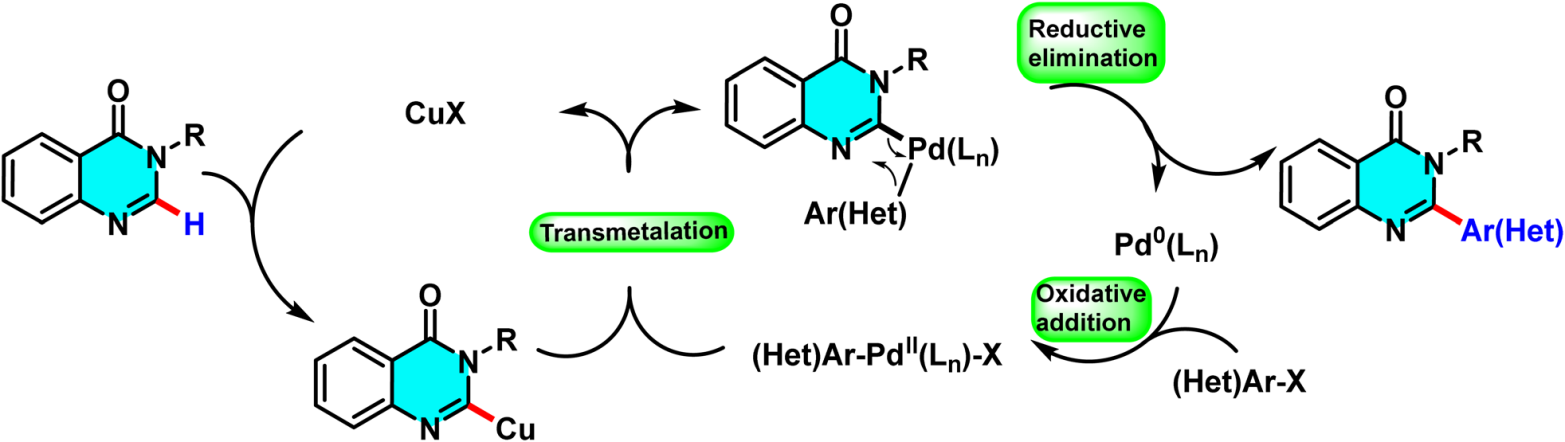
Plausible mechanism for arylation of quinazolin-4(3*H*)-ones.

The proposed mechanism, supported by control experiments and condition optimization, involves a cooperative action between copper and palladium. Initially, CuI coordinates with quinazoline and *t*-BuOLi to generate an active Cu complex. Pd(0), stabilized by the ligand, then undergoes oxidative addition with the aryl halide to afford an Ar–Pd(ii)–X intermediate. Subsequent transmetalation transfers the aryl group to quinazoline, and reductive elimination yields the 2-arylated product. Ligand effects are decisive: electron-rich PPh_3_ is optimal for bromides, whereas NiXantphos favors chlorides by stabilizing the Ar–Pd–Cl intermediate (Scheme 11).^67^

**Scheme 12:**
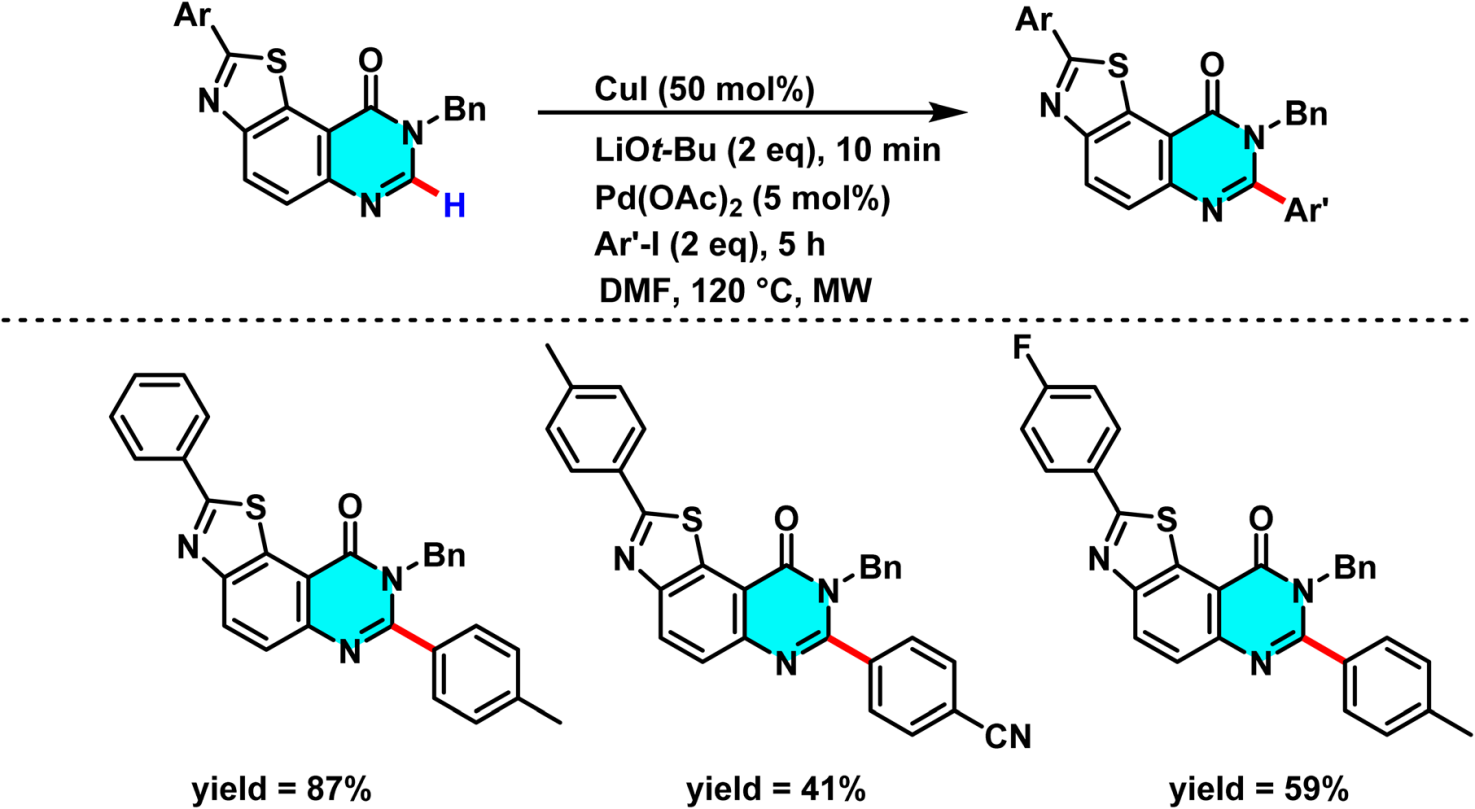
Pd(OAc)_2_-catalyzed activation of C(2)–H bonds in pyrimidine-containing structures.

Harari *et al.* developed a Pd(OAc)_2_/CuI-catalyzed method for selective C–H arylation at the C-2 and C-7 positions of pyrimidine rings. The reaction proceeds without directing groups, as Pd(ii) and *t*-BuOLi directly activate the pyrimidine core, while CuI enhances metalation efficiency and yield. This one-pot approach simplifies the process by eliminating intermediate isolation (Scheme 12).

The reaction mechanism shows that the palladium catalyst first coordinates at the C-2 position of the pyrimidine ring to form a metalated intermediate, followed by electron transfer and aryl substitution. The activated catalyst then targets the C-7 position, repeating a similar arylation process. *t*-BuOLi acts as a key base, assisting proton abstraction and enhancing pyrimidine reactivity to rapidly generate the Pd intermediate. Moreover, sequential arylation at the C-2 and C-7 positions underscores the method's versatility for structural modification of pyrimidine frameworks.^68^

**Scheme 13:**
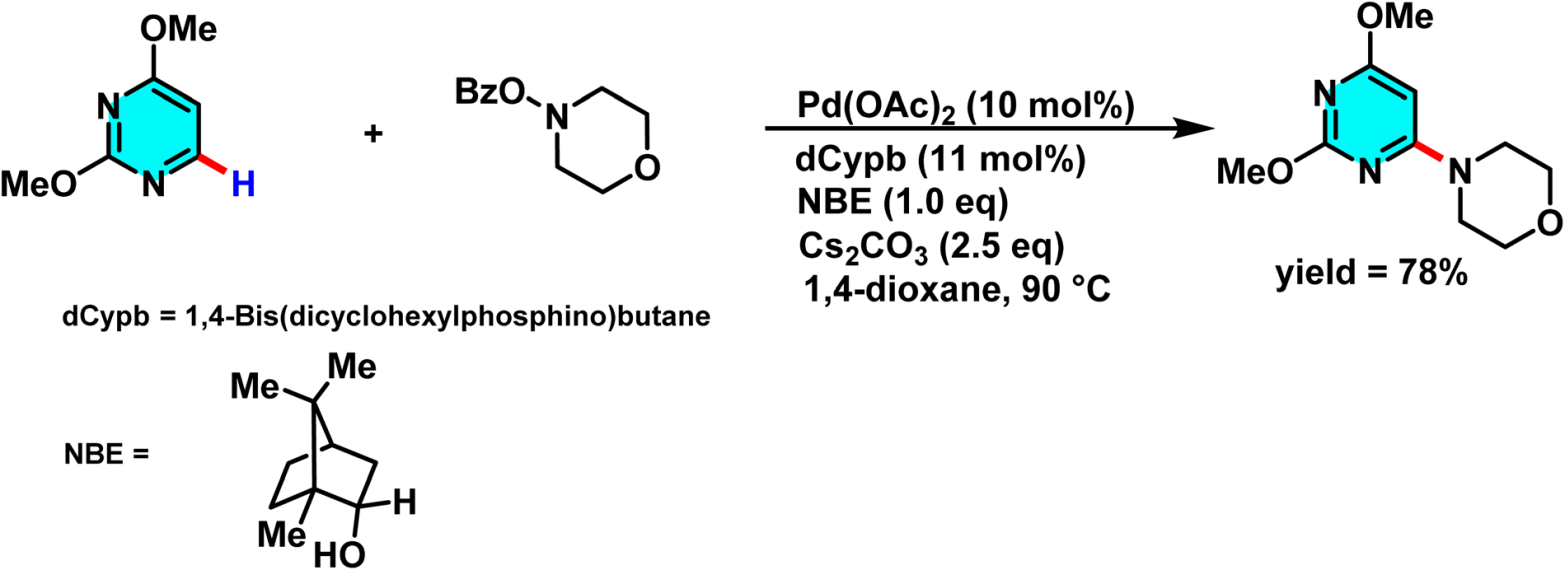
Pd/norbornene-catalyzed C(2)-amination of pyrimidine using *O*-benzoyl hydroxylamines as electrophiles.

Dong and colleagues developed a new method for C–H bond activation in heterocyclic systems, especially pyrimidine bromides, using a palladium/norbornene (Pd/NBE) catalytic system. This approach, which outperforms classical methods such as Suzuki–Miyaura coupling in terms of efficiency, enables selective C–H activation at the *ortho* position and facilitates *ipso*/*ortho* functionalization reactions. The catalyst Pd(OAc)_2_, in conjunction with norbornene, initiates the process through oxidative addition to the C–Br bond, forming an active intermediate palladium complex that plays a crucial role in controlling reaction selectivity. Additionally, the inclusion of phosphine ligands such as DPEphos and dCypb enhances the stability of the palladium complex and improves reaction yields. Evaluation of a broad range of aromatic substituents demonstrated high compatibility with various compounds, including *O*-benzoyl hydroxylamines, carboxylic anhydrides, and alkyl halides (Scheme 13).

The mechanism begins with oxidative addition of Pd(0) to the C–Br bond of the aryl bromide, generating an aryl–norbornene–palladium (ANP) intermediate. Subsequent C–H metalation occurs at the C-2 position, followed by nucleophilic substitution at the *ipso* site. A final β-elimination step releases the product and regenerates the active palladium catalyst.^69^

**Scheme 14:**
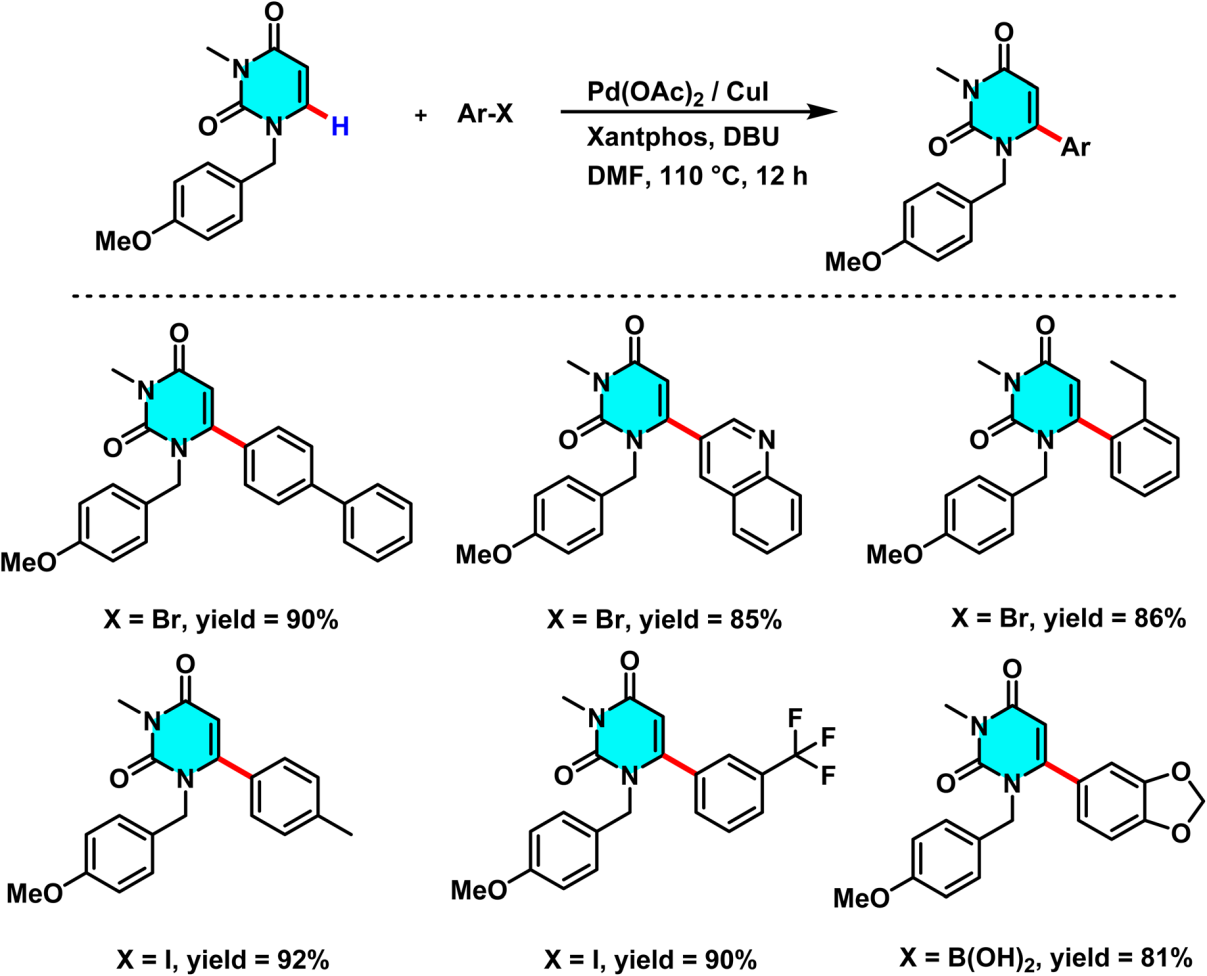
C(6)–H arylation of benzylated uracils, catalyzed by palladium(ii).

In 2019, Savitha and co-workers reported a Pd(OAc)_2_/CuI-catalyzed method for selective C(6)–H arylation of benzylated uracils. The Pd–Xantphos complex activates the C(6)–H bond, enabling efficient aryl transfer, while CuI improves metalation and yield. 1,8 Diazabicyclo[5.4.0]undec-7-ene (DBU) assists proton abstraction, and DMF provides optimal stabilization of the catalytic species. Aryl iodides and bromides show higher reactivity than chlorides, and aryl boronic acids can also participate with moderate yields (Scheme 14).

The reaction mechanism involves the formation of an intermediate palladium complex at the C-6 position, followed by the oxidative addition of an aryl halide or boronic acid to Pd(ii), and the transfer of the aryl group *via* a CMD pathway. Reductive elimination subsequently yields the final product and regenerates the palladium catalyst for further cycles.^70^

**Scheme 15:**
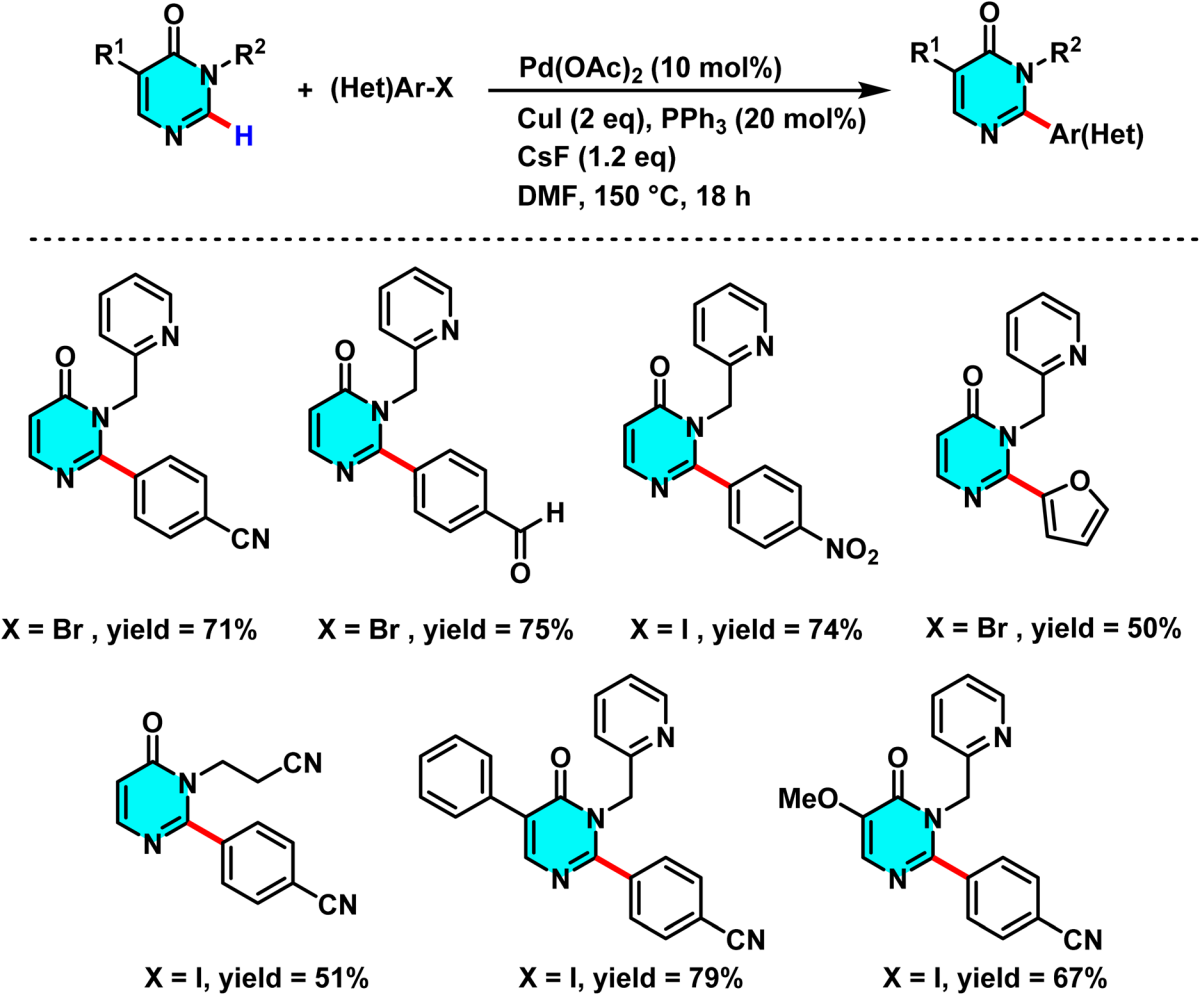
C–H arylation at the C-2 position of pyrimidin-4-ones *via* utilizing Pd(ii) catalyst with Cu(i) as cocatalyst.

In 2020, Ruiz *et al.* developed a new method for selective C–H arylation at the C-2 position of pyrimidin-4-ones using a Pd(ii) catalyst and Cu(i) as a cocatalyst. This approach employs activating groups such as picolinyl and cyanoethyl to facilitate metalation, enabling direct coupling of pyrimidine with aryl halides without the need for substrate pre-derivatization. Investigations revealed that the use of Pd(OAc)_2_ as the catalyst, CsF as the base, and CuI as the cocatalyst significantly enhances reaction yield and selectivity at the C-2 position. This process not only ensures precise positional control but also allows for the synthesis of 2-aryl and 2,5-diaryl pyrimidinone derivatives under mild conditions with high efficiency (Scheme 15).

**Scheme 16:**
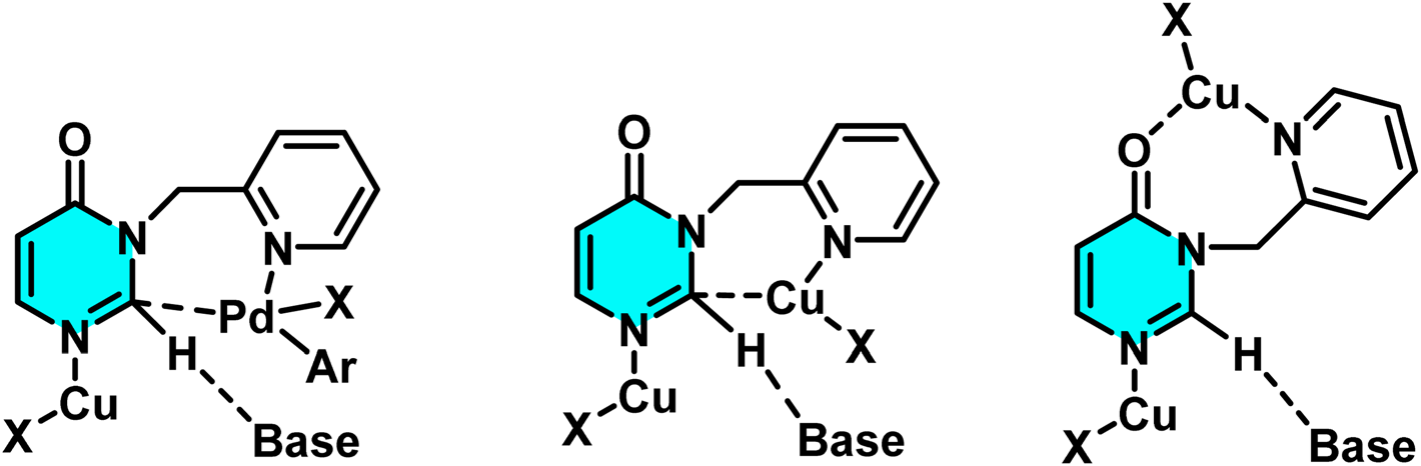
Possible models of C–H activation of pyrimidin-4-ones.

The reaction mechanism involves the formation of a palladium complex at the C-2 position, followed by the transfer of an aryl group from the aryl halide and the formation of a new C–C bond *via* a CMD pathway. Subsequent reductive elimination releases the arylated product, regenerating the palladium catalyst (Scheme 16).^71^

**Scheme 17:**
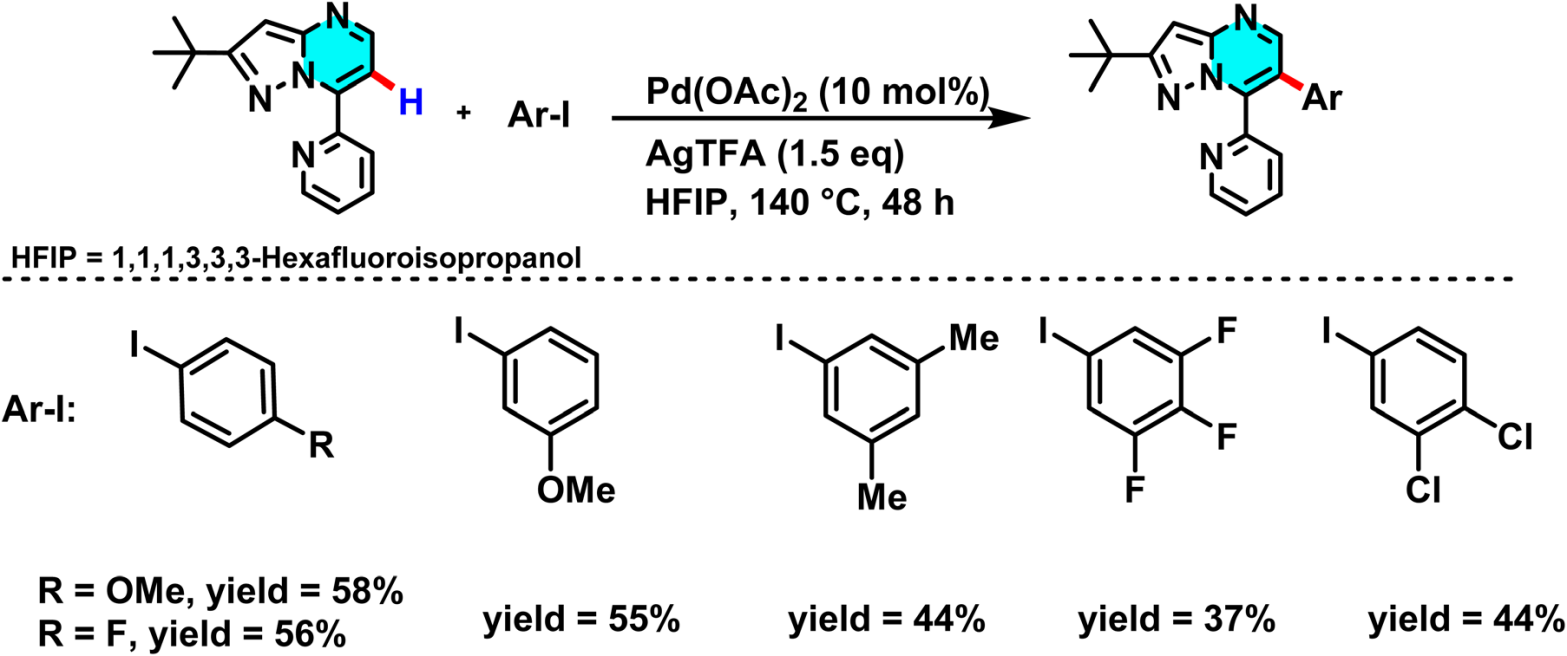
Pd(ii) catalyzed C(sp^2^)–H arylation 7-pyridyl-pyrazolo[1,5-*a*]pyrimidine.

In 2020, the Gogula research group developed a new method for selective C–H activation of pyrimidine derivatives using a Pd(ii) catalyst and temperature modulation to guide reaction pathways. This investigation demonstrated that at 120 °C, the reaction activates the C(sp^3^)–H bond, whereas elevating the temperature to 140 °C shifts the pathway toward C(sp^2^)–H arylation. The core structure, 7-pyridyl-pyrazolo[1,5-*a*]pyrimidine, plays a crucial role in guiding the palladium catalyst's selectivity, providing favorable conditions for the formation of active palladium complexes. The presence of AgTFA as an oxidant sustains the Pd(ii) catalytic cycle and enhances reaction yields (Scheme 17).

**Scheme 18:**
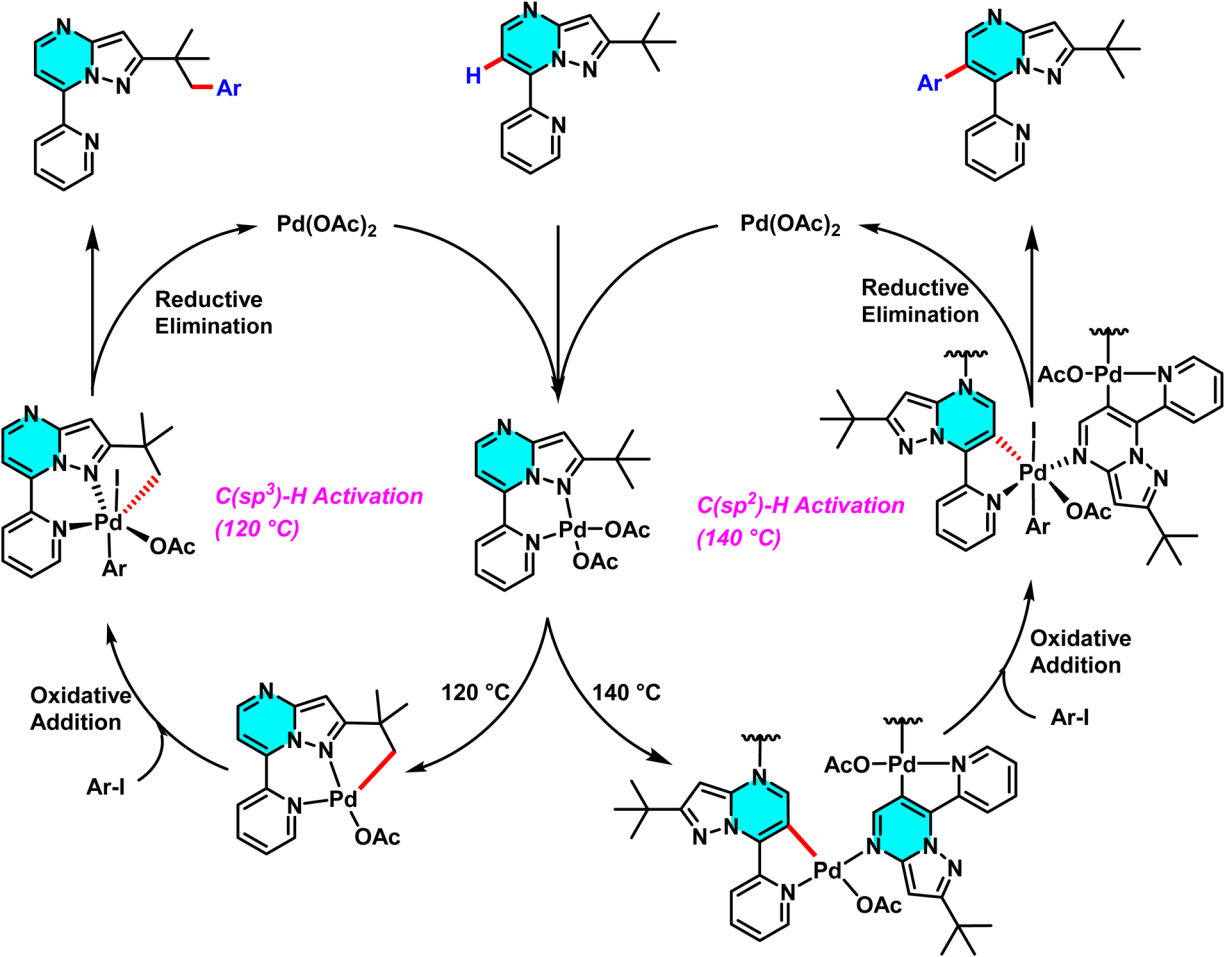
Proposed pathway for C–H activation of 7-pyridyl-pyrazolo[1,5-*a*]pyrimidine in temperature modulation.

The reaction mechanism involves the formation of an intermediate palladium complex, which transforms into either a [6,5]-fused palladacycle for the C(sp^3^)–H pathway or a 16-membered tetrameric complex for the C(sp^2^)–H one (Scheme 18).^72^

**Scheme 19:**
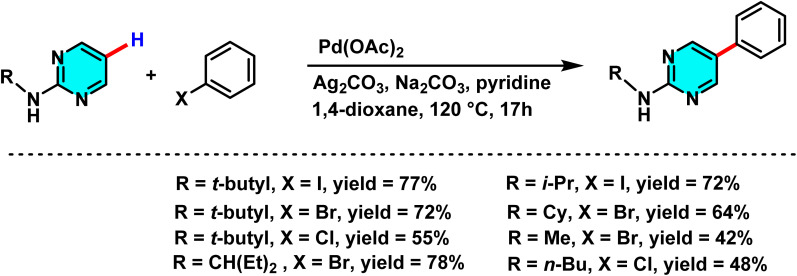
Representative C(5)–H arylation of 2-aminopyrimidine derivatives under Pd(ii) catalysis.

**Scheme 20:**
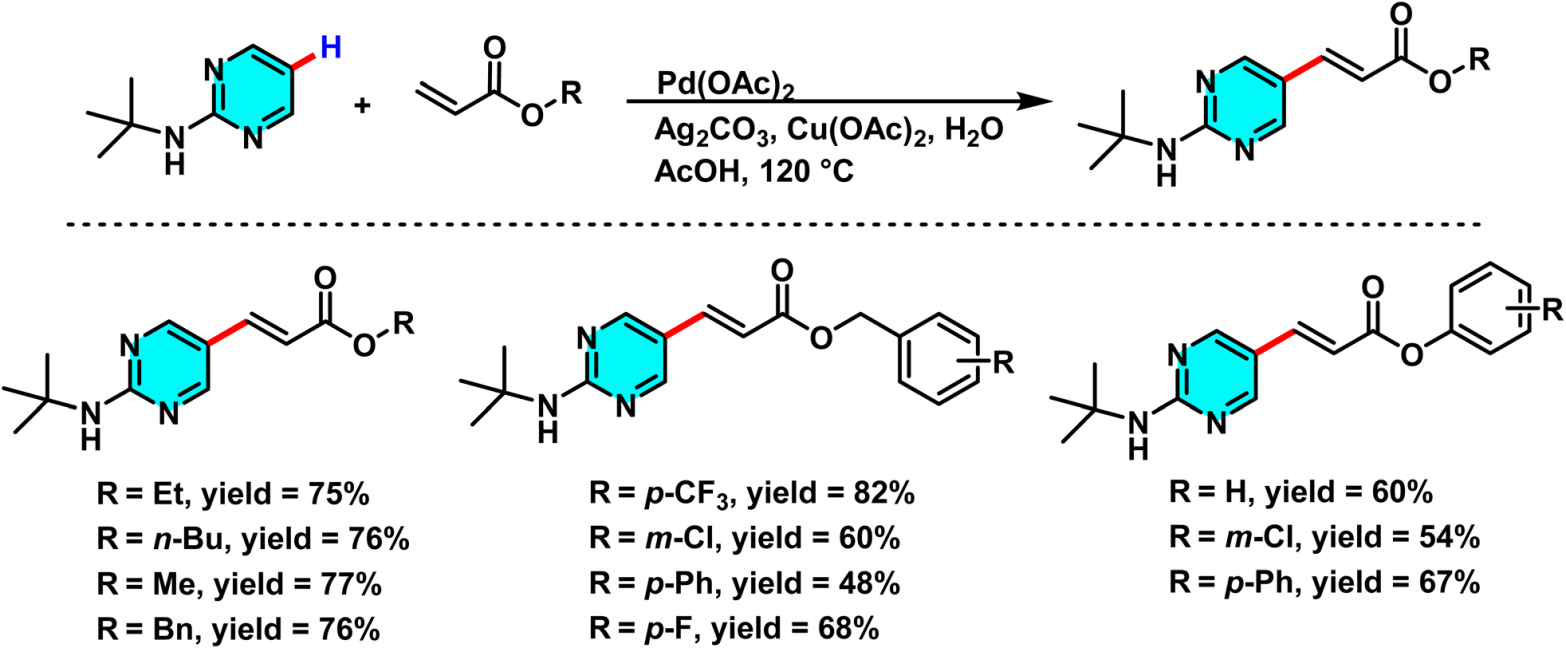
Olefination at the C-5 position of 2-aminopyrimidines derivatives under Pd(ii) catalysis.

Das and co-workers in 2020, developed a novel method for the selective C–H arylation and olefination at the C-5 position of 2-aminopyrimidines, catalyzed by either Pd(ii) or Pd(0) species. The C–H arylation proceeding through a Pd(ii)/Pd(iv) cycle, employs aryl halides as coupling partners and is carried out in 1,4-dioxane using Pd(OAc)_2_ as the catalyst and Na_2_CO_3_ as base (Scheme 19). The base assists in deprotonating the N–H group and enhancing electron density at the C(5)-position, thereby promoting electrophilic palladation. In contrast, the olefination pathway proceeds *via* a Pd(0)/Pd(ii) mechanism and utilizes alkenes under oxidative conditions in the presence of Cu(OAc)_2_·H_2_O and Ag_2_CO_3_, with AcOH as the reaction medium (Scheme 20).

**Scheme 21:**
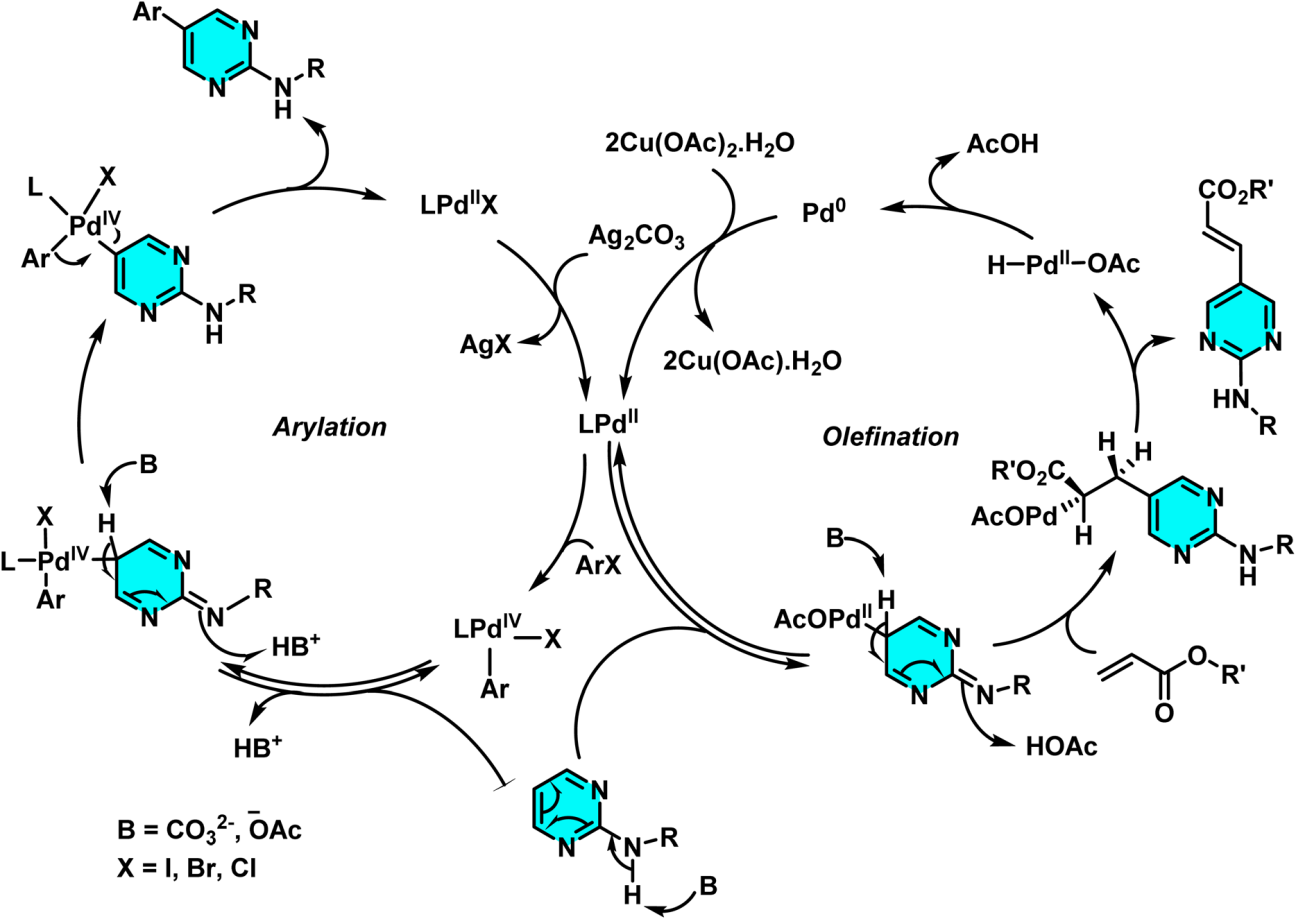
Proposed mechanism for C(5)–H arylation and olefination of 2-aminopyrimidines derivatives under Pd(ii)/Pd(iv) catalysis.

The reaction mechanism involves the formation of an active palladium–carbonyl complex, followed by metalation of the C(5)–H bond of pyrimidine and electron transfer to generate a palladium intermediate species. Subsequently, the aryl or alkene group couples with the pyrimidine ring, and after reductive elimination and rearomatization, the final arylated or alkenylated product is released (Scheme 21).^73^

**Scheme 22:**
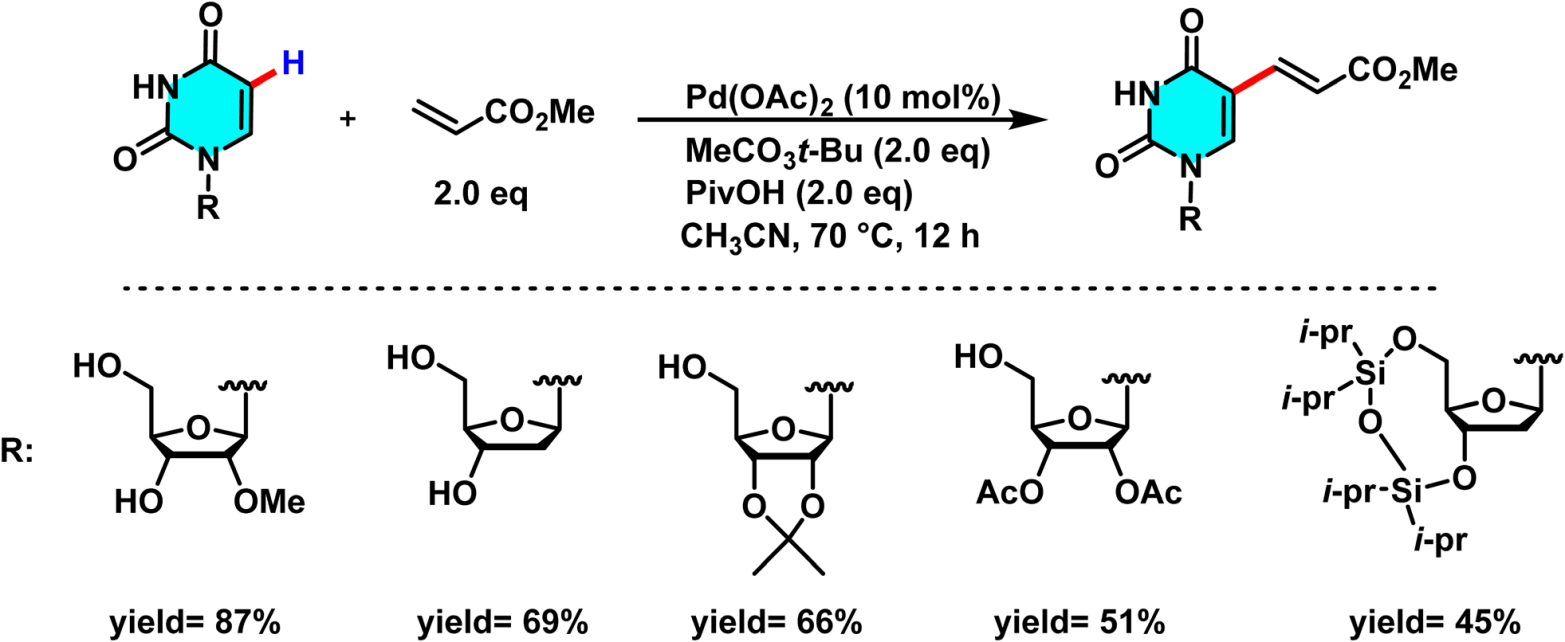
C(5)–H olefination of uridine, deoxyuridine in the presence of Pd(OAc)_2_.

In 2022, Zhao and co-workers developed a new method for selective C(5)–H olefination of uridine, deoxyuridine, and uridine monophosphate. It is catalyzed by Pd(ii) and utilizes CH_3_CO_3_*t*-Bu as the oxidant. The catalyst Pd(OAc)_2_ plays a central role in activating the C(5)–H bond through the formation of a palladium complex, enabling alkenyl coupling. The additive PivOH enhances the reaction yield by lowering the activation energy and improving the metalation process (Scheme 22).^74^

The uridine derivatives, such as deoxyuridine and uridine monophosphate, play critical roles in biological processes, including gene expression regulation and nucleotide metabolism.^75^ The reaction proceeds through C(5)–H metalation to form a palladium intermediate in the presence of Pd(OAc)_2_ and PivOH. Subsequent oxidative addition of the alkene produces a Pd(ii)–alkenyl complex, which undergoes CMD to form a new C–C bond at the C-5 position. Reductive elimination then releases the product and regenerates Pd(ii) for further catalysis.^74^

**Scheme 23:**
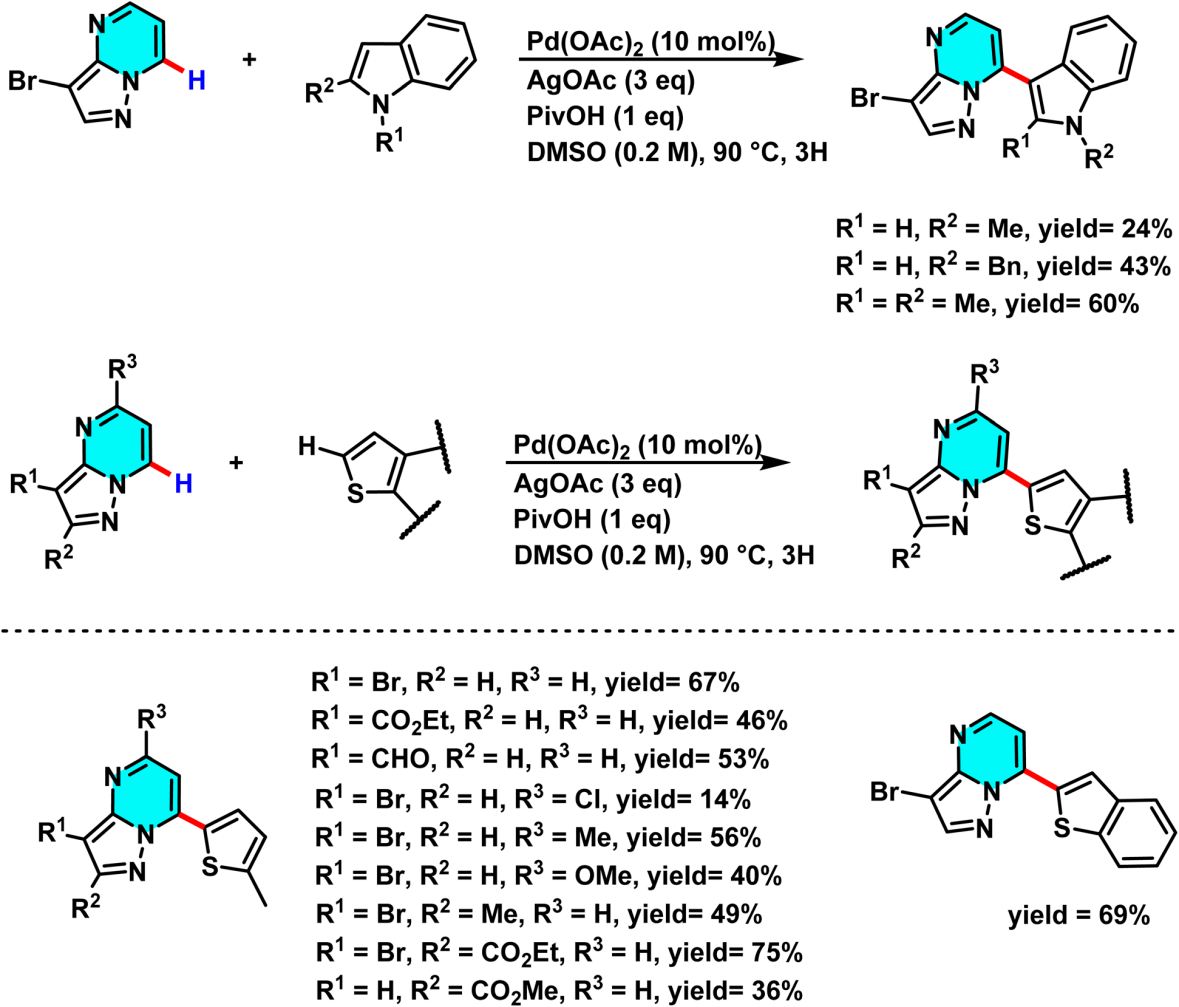
Pd(OAc)_2_-catalyzed reaction between pyrazolo[1,5-*a*]pyrimidines and five-membered heterocyclic.

In 2022, Nguyen *et al.* developed a Pd(OAc)_2_-catalyzed oxidative C–H/C–H cross-coupling of pyrazolo[1,5-*a*]pyrimidines with five-membered heterocycles. Using AgOAc as an oxidant and PivOH as an additive in DMSO at 90 °C, the reaction proceeds under mild conditions with high selectivity at the C(7)–H position. The method enables direct coupling with various heterocycles, including thiophene, benzothiophene, thiazole, furan, oxazole, indole, and imidazo[1,2-*a*]pyridine, without the need for directing or activating groups. Pd(OAc)_2_ facilitates metalation and formation of a palladium intermediate that drives the coupling process efficiently (Scheme 23).

**Scheme 24:**
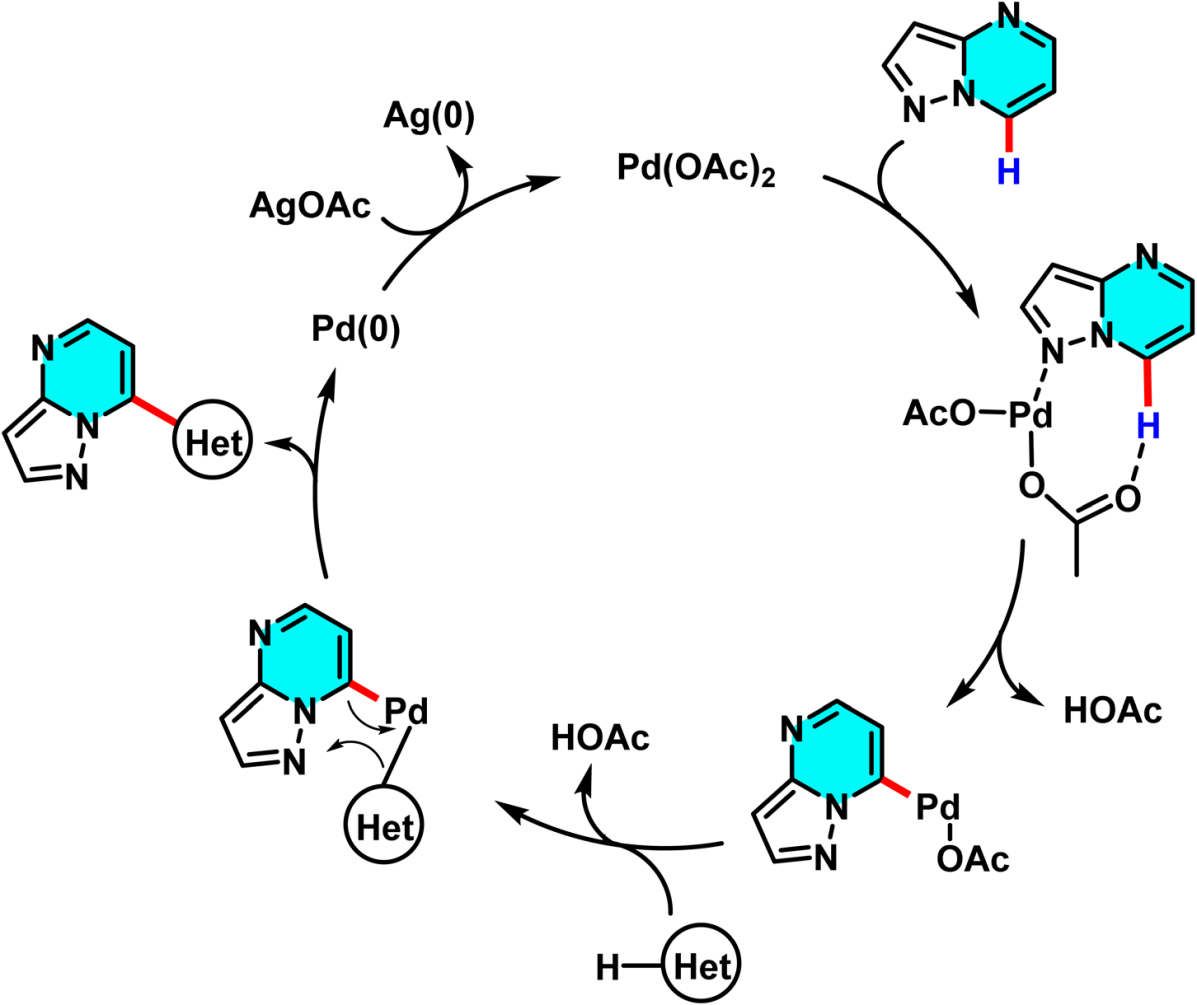
Proposed pathway for Pd(OAc)_2_-catalyzed reaction between pyrazolo[1,5-*a*]pyrimidines and five-membered heterocyclic.

The mechanism starts with coordination of Pd(OAc)_2_ to the C(7)–H bond, forming a palladium intermediate. AgOAc then promotes metalation–dehydrogenation to generate a new Pd species (Scheme 24). Electron transfer between the activated substrates enables cross-coupling of the pyrimidine and the five-membered heterocycle. Finally, Pd is reoxidized to Pd(ii) by AgOAc to complete the catalytic cycle. Among the tested heterocycles, thiophene and thiazole afforded the highest yields.^76^

**Scheme 25:**
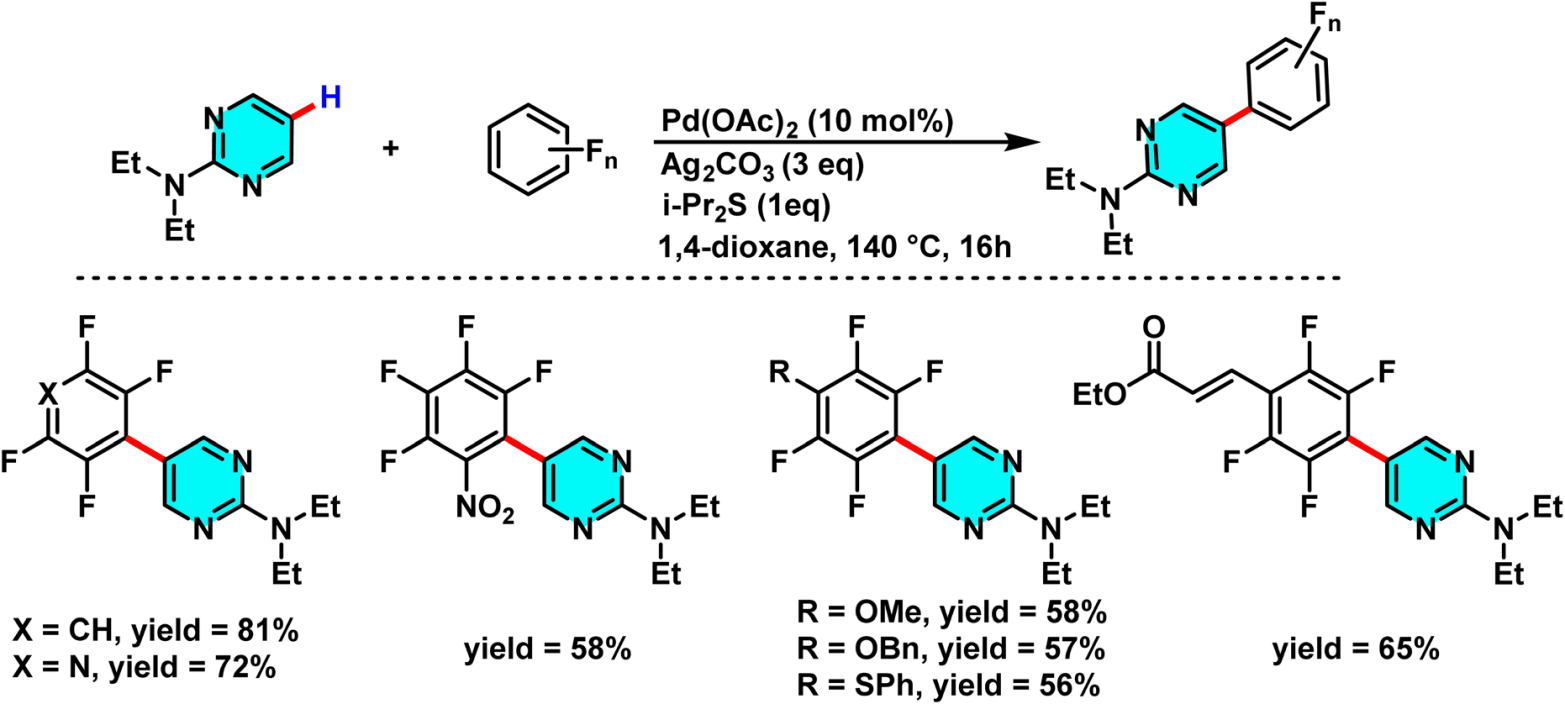
Pd(ii)-catalyzed C(5)–H alkylation of 2-aminopyrimidine derivatives.

Along these lines, in 2024, Das and Maji introduced a novel and controlled method for the selective polyfluoroarylation of C–H and N–H bonds in 2-aminopyrimidines, catalyzed by Pd(ii)/Pd(0) systems. This study demonstrated that the reaction pathway can be directed toward either C-5 arylation or N–H arylation depending on the nature of the substituent attached to the nitrogen. When an alkyl substituent is present, the polyfluoroarene reactant targets the C-5 position, forming a new C–C bond, whereas an aromatic substituent shifts the reaction toward *N*-arylation. The process employs Pd(OAc)_2_ as the catalyst, Ag_2_CO_3_ as the oxidant, and i-Pr_2_S as the ligand, achieving high yields in 1,4-dioxane as solvent. The mechanism proceeds *via* an active Pd–polyfluoroaryl species, with the reaction pathway governed by the amine substituent. For alkyl amines, migration of the Pd complex to the C-5 position affords the C(5)-arylated product. In contrast, aromatic amines undergo reductive elimination from a four-membered palladacycle to yield the *N*-polyfluoroarylated product (Scheme 25).

**Scheme 26:**
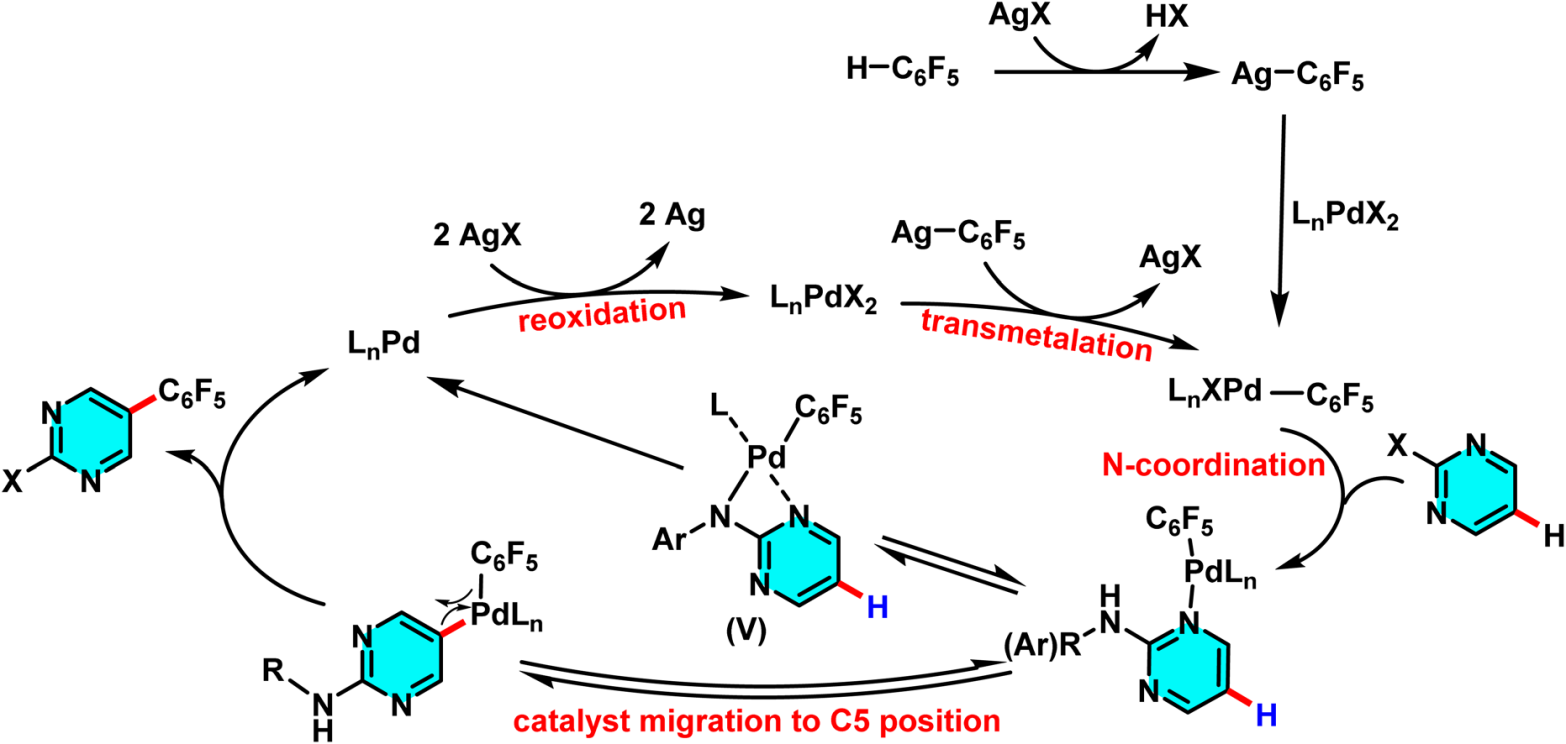
Proposed mechanism for Pd(ii)-catalyzed C(5)–H alkylation of 2-aminopyrimidine derivatives.

This mechanism diverges from traditional approaches that often require directing groups and harsh conditions, operating instead under mild conditions with exceptional selectivity. Beyond its high efficiency and broad adaptability to various 2-aminopyrimidine derivatives and polyfluoroarenes, this method offers significant potential for applications in the development of pharmaceutical compounds, bioactive materials, and electroactive substances (Scheme 26).^77^

### 2.2 Copper-catalyzed C–H activation of pyrimidines

Copper has garnered attention as a cost-effective and readily available catalyst for C–H activation in heteroarenes, including pyrimidines. This metal enables a variety of reactions with satisfactory yields and is explored in four key studies reviewed herein.

**Scheme 27:**
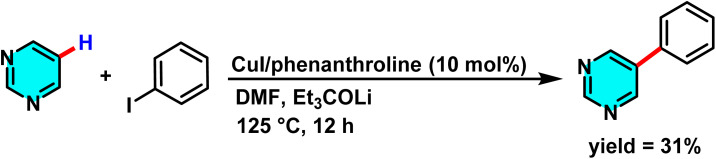
CuI-catalyzed arylation of pyrimidine.

In a research conducted by Daugulis and co-workers in 2008, the arylation reaction of aromatic and heterocyclic compounds was investigated. This research team utilized copper iodide as a catalyst, phenanthroline as a ligand, aryl halides as the reacting species, and lithium 3-ethylpentan-3-olate (Et_3_COLi) as a base to synthesize successfully novel aromatic compounds (Scheme 27).

The study found that pyrimidine showed low reactivity in this reaction due to its low acidity. Its electron-deficient ring hindered the arylation process, causing slow reaction kinetics and low product yield compared to consumed starting materials. However, the most acidic hydrogen in pyrimidine acted as the target for nucleophilic attack, enabling aryl group attachment at this site. For successful arylation of electron-deficient heterocycles, the p*K*_a_ value (indicating acidity) must be below 35.^78^

**Scheme 28:**
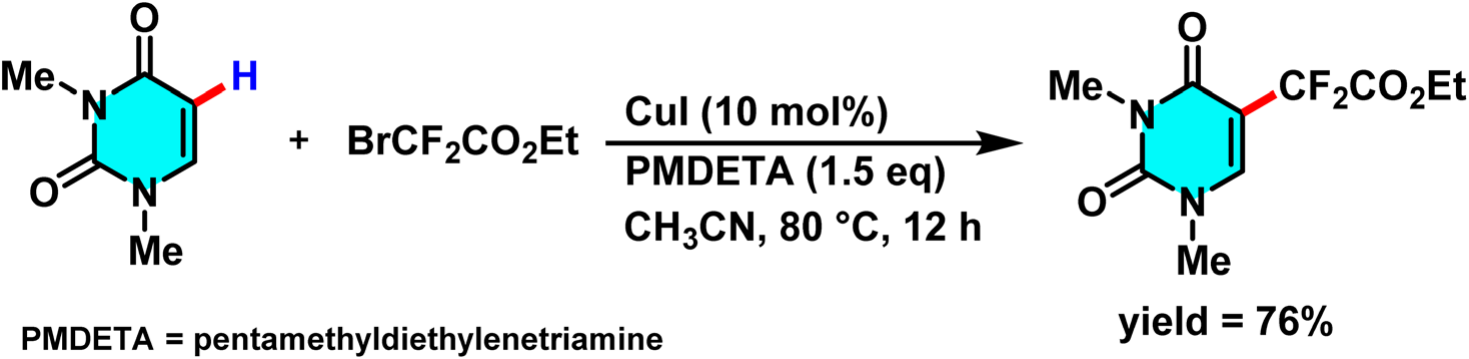
Cu(i)-catalyzed C–H difluoroalkylation of pyrimidine.

In 2017 Wang *et al.* introduced an innovative approach for the difluoroalkylation of pyrimidine and other heterocyclic compounds *via* C–H activation. The optimized reaction conditions involve the use of (CuI) at (10 mol%) as the catalyst, pentamethyldiethylenetriamine (PMDETA) serving dual roles as both ligand and base (1.5 eq.), acetonitrile (CH_3_CN) as the solvent, a temperature of 80 °C, and an argon atmosphere for 12 hours. The selection of PMDETA is critical due to its ability to form stable complexes with copper and neutralize byproduct acids, achieving yields as high as 97% for model substrates such as styrene (Scheme 28).

**Scheme 29:**
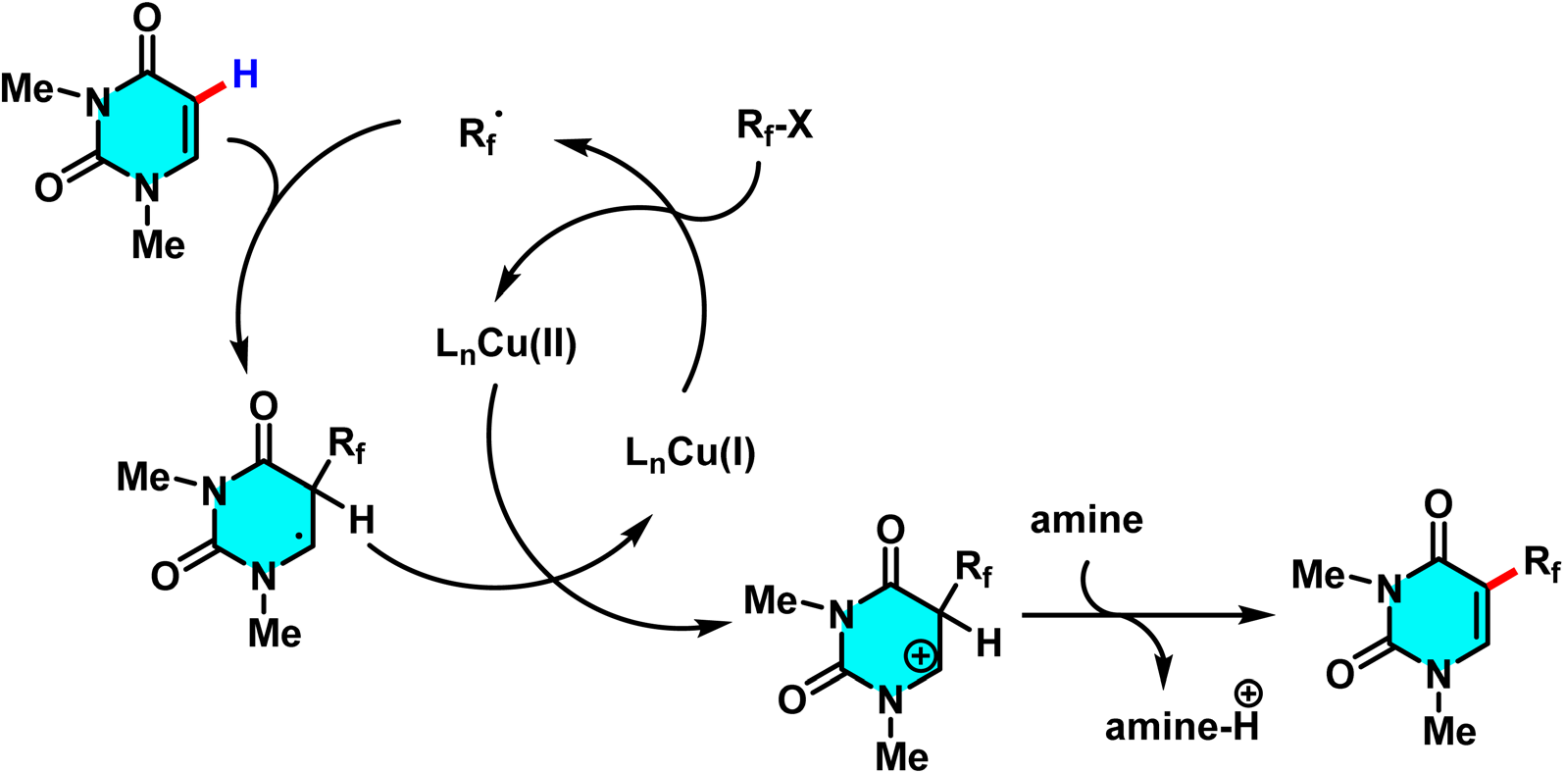
Plausible mechanism for C–H difluoroalkylation of pyrimidine.

The proposed mechanism relied on a radical-based process, starting with the formation of the [CuI(PMDETA)] complex. This complex reacted with a fluoroalkyl halide, like BrCF_2_CO_2_Et, producing the ˙CF_2_CO_2_Et radical *via* a single-electron transfer (SET) mechanism, oxidizing copper from Cu(i) to Cu(ii). The radical then added to the pyrimidine C–H bond, and the Cu(ii) complex stabilized the reaction, forming the fluoroalkylated product while regenerating CuI to complete the catalytic cycle. CuI acted as a recyclable catalyst, and PMDETA served as a stabilizing and neutralizing agent, allowing minimal catalyst use with high yields, demonstrating the method's practicality and efficiency (Scheme 29).^79^

**Scheme 30:**
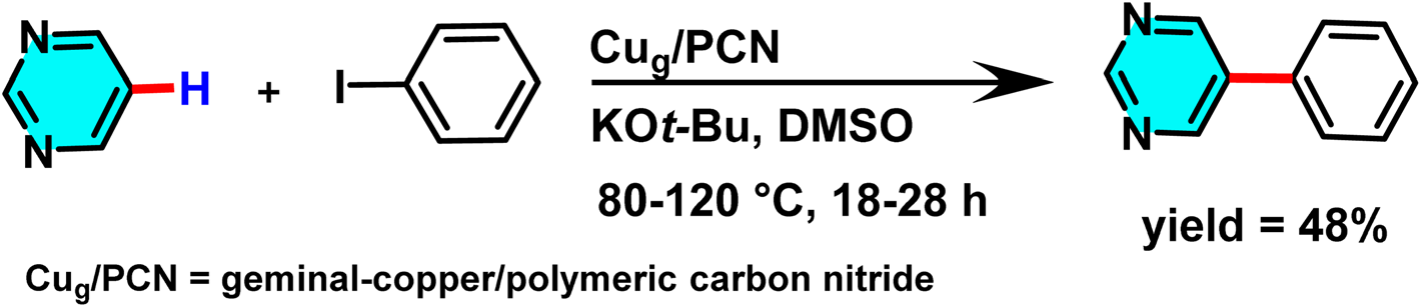
Cu_g_/PCN-catalyzed arylation of pyrimidine with aromatic rings.

In 2023, researchers investigated geminal-atom catalysts (GACs) as a new class of heterogeneous catalysts. In this system, metal atoms, particularly copper, are immobilized in close proximity on a solid support, creating a unique spatial arrangement that enhances synergistic interactions between metal centres and facilitates complex organic transformations. This precisely designed catalytic architecture has demonstrated high efficiency in catalyzing C–C coupling reactions between pyrimidine and aromatic rings. In this process, the Cu_g_/PCN catalyst was employed in the presence of DMSO as a solvent to promote the coupling reaction (Scheme 30). The polymeric carbon nitride (PCN) support, composed of carbon and nitrogen, provides an ideal environment for stabilizing twin copper sites due to its porous structure and delocalized π-bonding framework. These features enable PCN to fine-tune the electronic and spatial environment of the active sites, thereby enhancing catalytic efficiency and improving the selectivity of coupling reactions.^80^

**Scheme 31:**
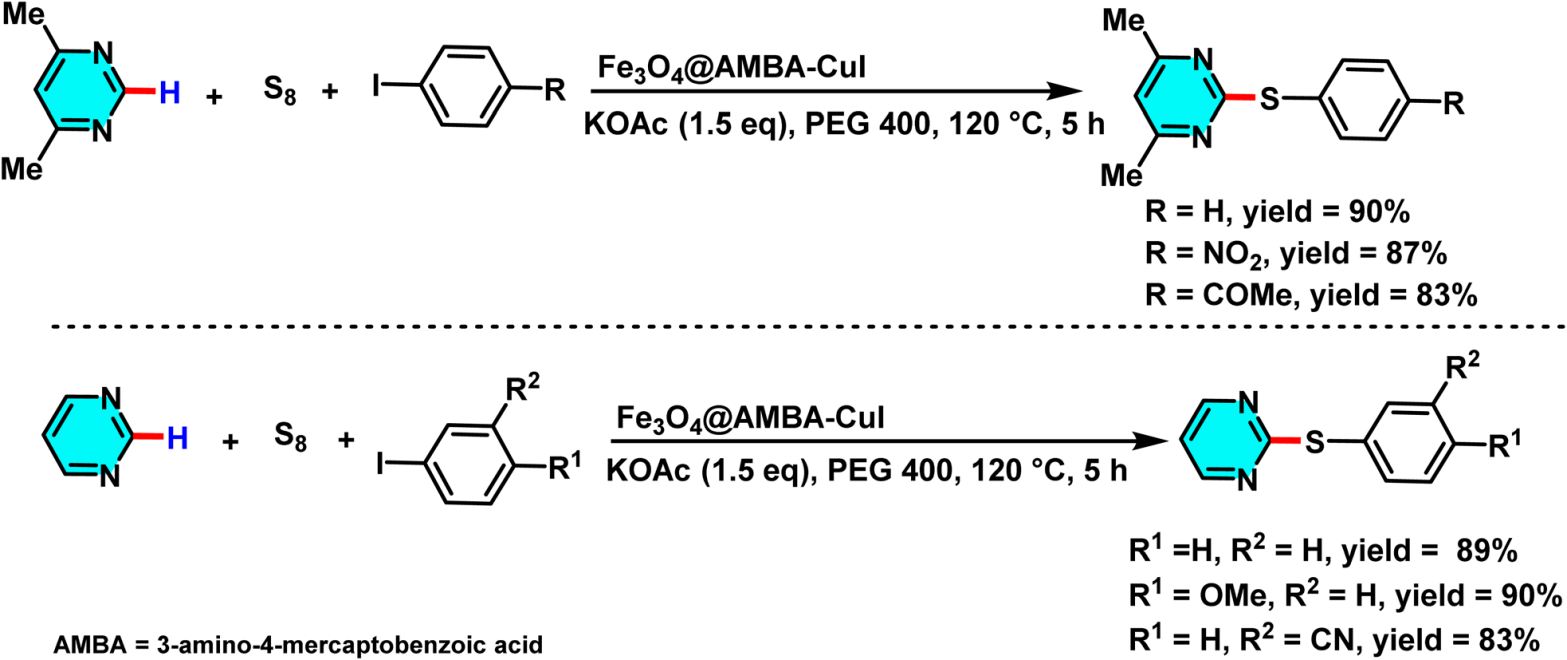
Sulfenylation of pyrimidines using Fe_3_O_4_@AMBA-CuI as a catalyst.

In a study conducted in 2024, the magnetic catalyst Fe_3_O_4_@AMBA-CuI was investigated as an efficient mediator for the sulfenylation reaction of heterocyclic compounds, particularly pyrimidines. The magnetic properties of this nanocatalyst facilitated its easy separation from the reaction mixture upon completion, enhancing its practical applicability (Scheme 31). To optimize the reaction conditions, various parameters such as catalyst loading, base selection, and solvent type were systematically evaluated. The results indicated that the use of potassium acetate as the base and polyethylene glycol (PEG) as the solvent led to the highest reaction yield.^81^

### 2.3 Rhodium-catalyzed C–H activation of pyrimidines

Rhodium, renowned for its high selectivity and ability to catalyze complex reactions, stands out as a prominent catalyst for C–H activation in pyrimidines.

**Scheme 32:**
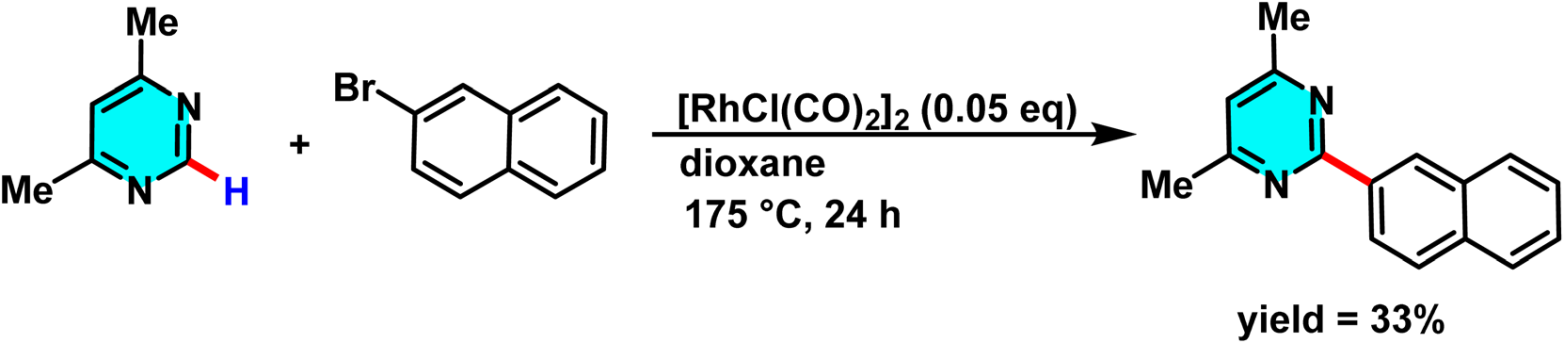
Rh(i)-catalyzed C–H activation of pyrimidines.

Bergman and co-workers in 2010, introduced an innovative method for C–H bond activation in azines, utilizing a Rh(i) catalyst. This process employs [RhCl(CO)_2_]_2_, a stable and commercially available catalyst, to facilitate the selective activation of the C–H bond the 2-position adjacent to a nitrogen atom in pyrimidine, facilitated by initial coordination of the nitrogen to the rhodium center, followed by C–H activation. Pyrimidine serves as a substrate, coupling with aryl bromides under standard conditions without requiring pre-activation. Aryl bromides act as sources of the aryl group, while solvents such as dioxane or toluene provide a suitable reaction medium. Conducted at 175 °C over 24 hours. This method achieves favorable yields across a broad range of functional groups, including chloro, fluoro, and ketone substituents (Scheme 32).

**Scheme 33:**
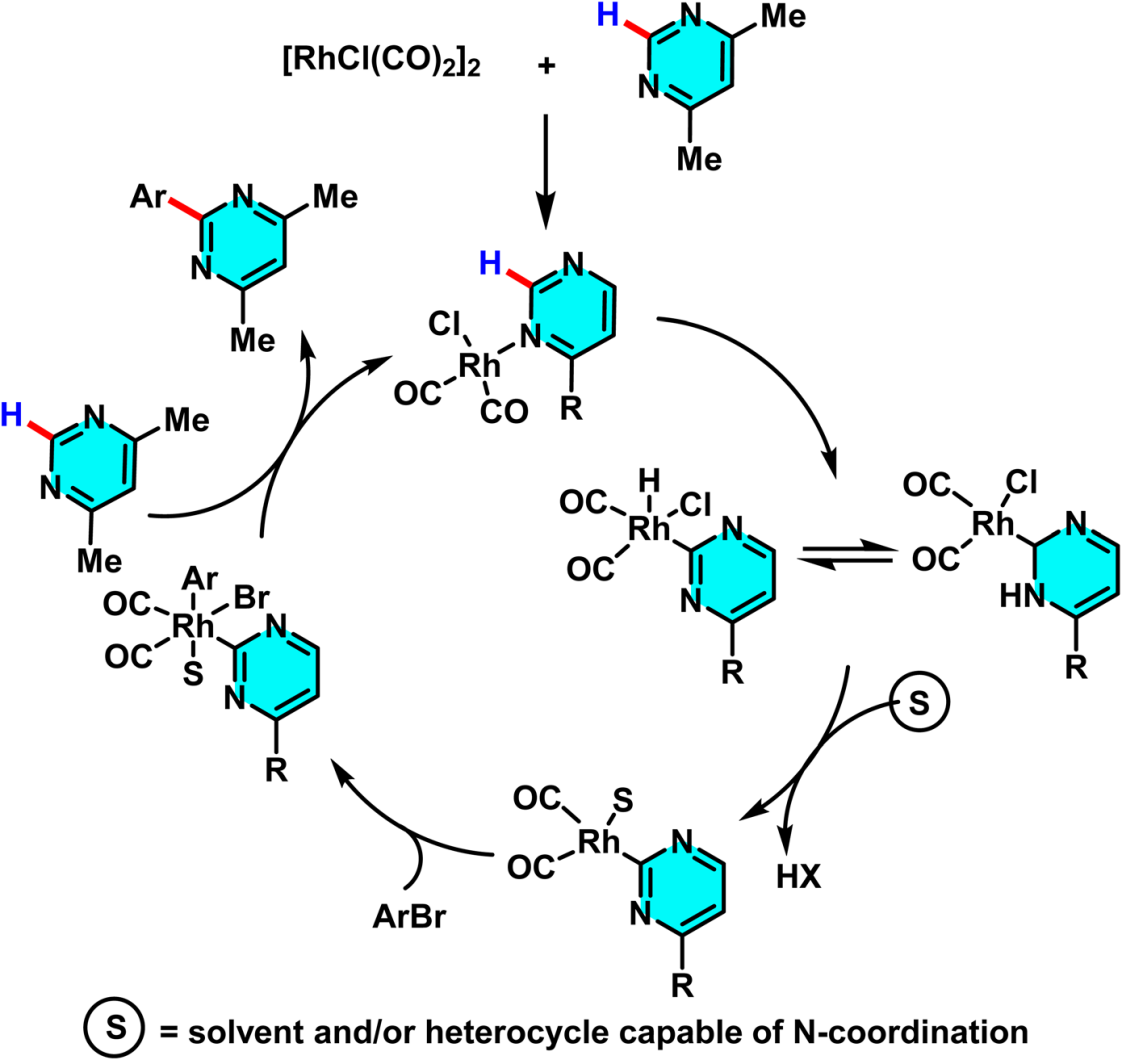
Suggested pathway for the catalytic arylation of pyrimidines using aryl bromides.

The reaction mechanism started with the pyrimidine nitrogen coordinating to the rhodium complex, followed by selective C–H activation at the C(2) position, forming an N-heterocyclic carbene (NHC) complex. This led to HX (*e.g.*, HBr) elimination, oxidative addition of aryl bromide or aroyl chloride, and, for aroyl chlorides, a decarbonylation step. The arylated product formed *via* reductive elimination, with the catalyst reduced and regenerated (Scheme 33).^82^

**Scheme 34:**
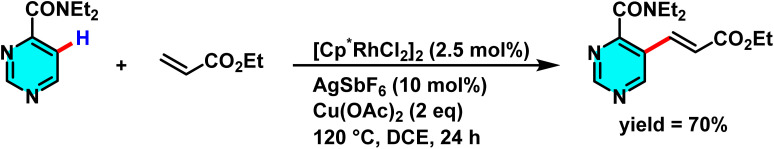
Oxidative olefination of pyrimidine catalyzed by Rh(iii) with Cu(OAc)_2_ as an oxidant.

In 2014, Zhou and co-workers reported a Rh(iii)-catalyzed oxidative olefination of pyrimidine and related N-heterocycles *via* direct C–H activation. The reaction uses [Cp*RhCl_2_]_2_ as the catalyst, AgSbF_6_ as an activator, and Cu(OAc)_2_ as an oxidant in 1,2-dichloroethane (DCE). The Rh(iii) complex selectively metalates the pyrimidine ring and couples with alkenes to form C=C bonds. AgSbF_6_ generates the active Rh species, while Cu(OAc)_2_ regenerates the catalyst (Scheme 34).

**Scheme 35:**
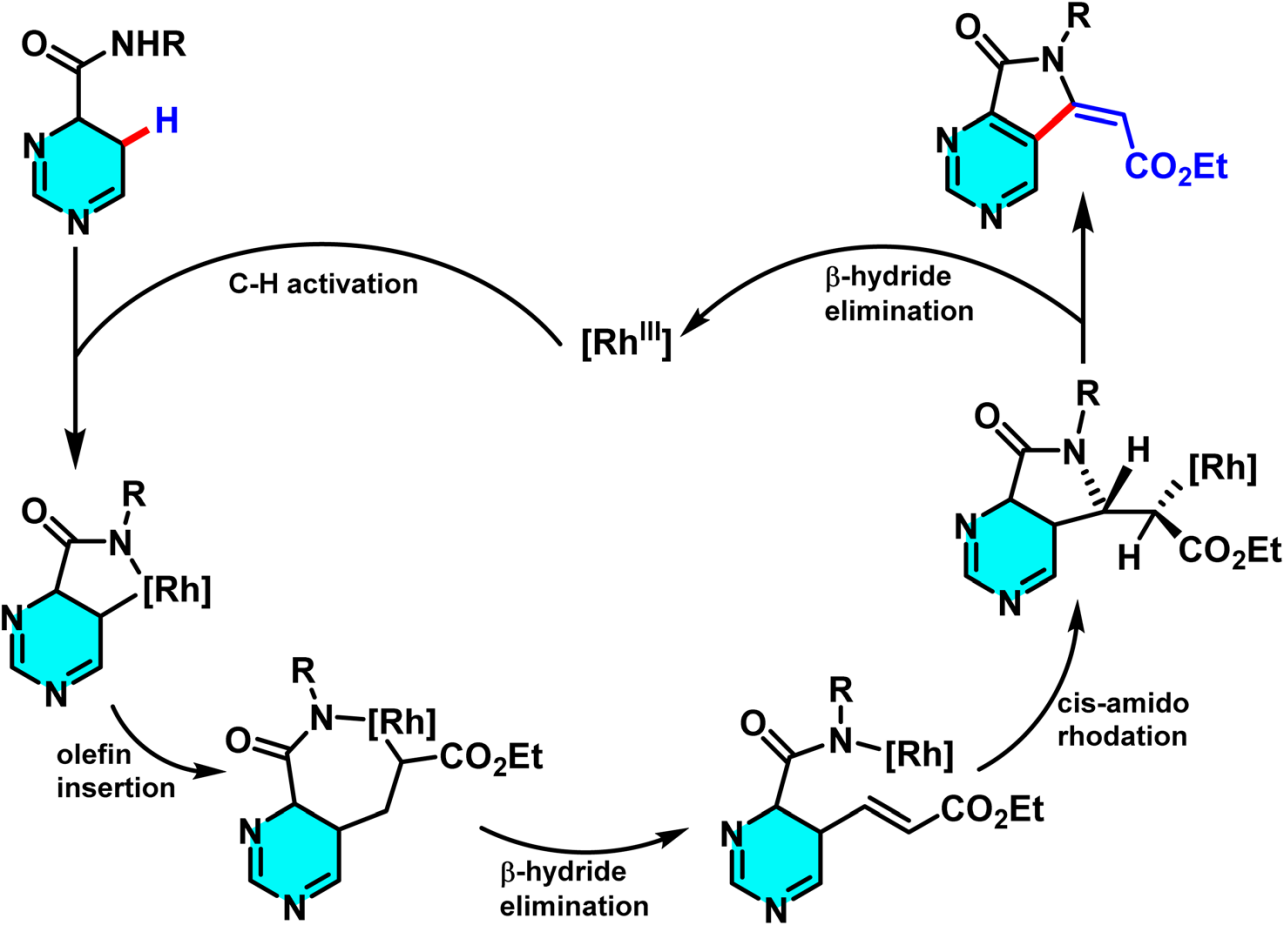
Olefination–cyclization reaction of secondary amides and the suggested mechanism.

The reaction mechanism involved several steps. The [Cp*RhCl_2_]_2_ complex activated with AgSbF_6_ formed a cationic Rh(iii) species for C–H metalation. This activated the pyrimidine C–H bond, creating a Rh–C metal intermediate that reacted with the alkene to form a rhodium–alkene complex. After electron transfer and C=C bond formation, Cu(OAc)_2_ oxidized the intermediate, causing pyrimidine ring rearomatization and yielding the product while regenerating the catalyst (Scheme 35).^83^

**Scheme 36:**
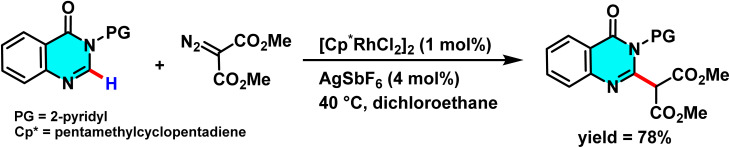
Rh(iii)-catalyzed alkylation of pyrimidine derivatives under mild condition.

Samanta *et al.* developed an effective Rh(iii)-catalyzed method for the direct and selective C-6 alkylation of pyrimidine derivatives under mild conditions. The transformation employs [Cp*RhCl_2_]_2_ (1 mol%) as the catalyst, with AgSbF_6_ (4 mol%) serving as a cocatalyst to generate the active cationic Rh(iii) species responsible for C–H activation. α-Diazocarbonyl compounds, such as diazomalonates, act as alkyl sources, while a nitrogen-containing aromatic directing group attached to the heterocycle governs the regioselectivity toward the C(6)-position. The reaction proceeds in solvents such as DCE or alcohols, at temperatures ranging from 40 to 100 °C over 6 to 24 hours, producing nitrogen (N_2_) as the sole byproduct (Scheme 36).

**Scheme 37:**
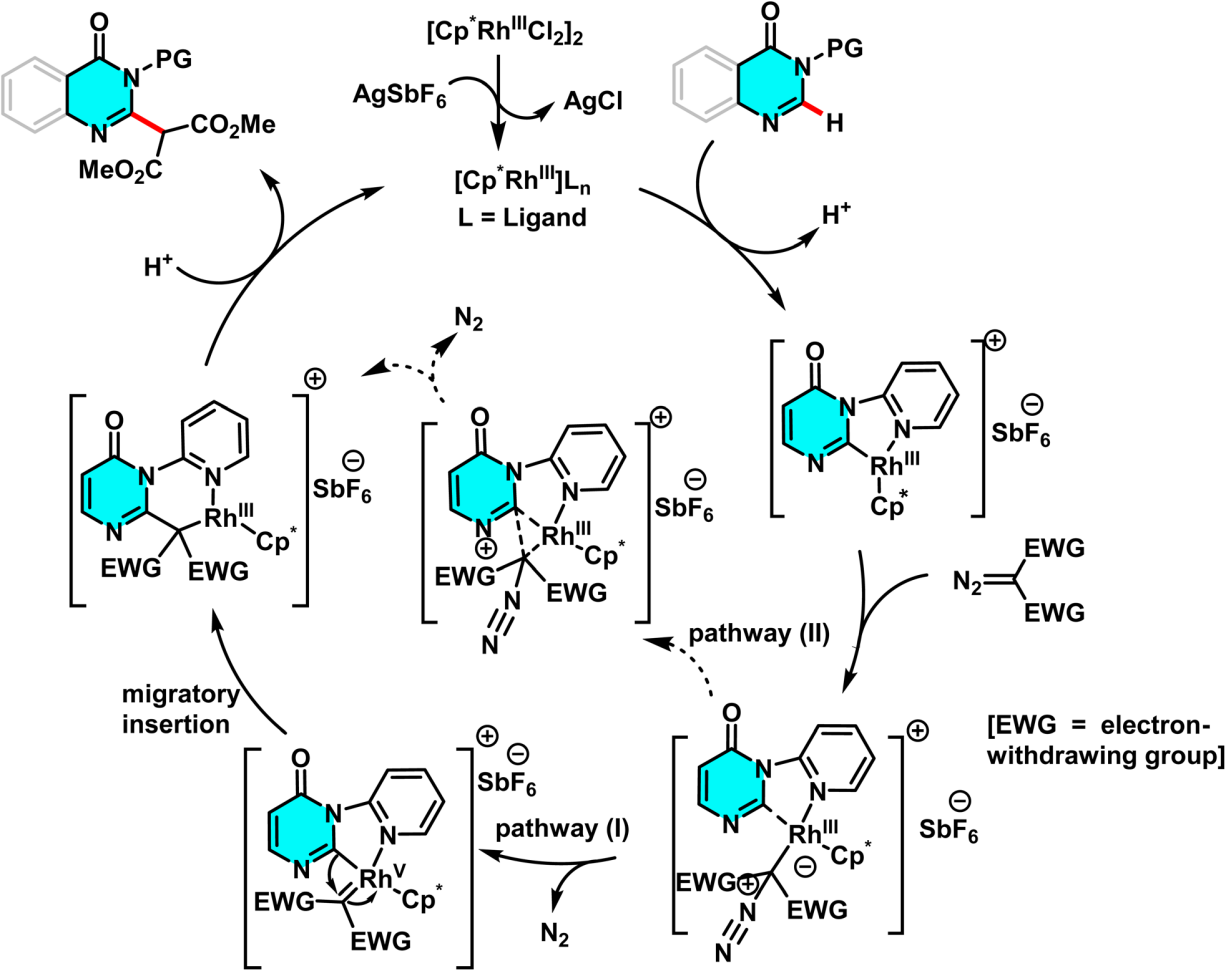
Proposed mechanism for alkylation of pyrimidine derivatives using Rh(iii) as catalyst.

The cationic Rh(iii) species coordinated with the nitrogen of the directing group, enabling electrophilic cleavage at the C(6)–H bond to form a cyclometalated intermediate. The diazo compound then coordinated to the metal center, initiating alkyl group incorporation *via* an oxidative carbene pathway or a non-redox insertion, both forming a C–C bond. Final protonolysis produced the C(6)-alkylated product and regenerated the Rh(iii) catalyst (Scheme 37), Control experiments showed C–H activation preceded carbene formation, and the directing group was crucial for regioselectivity.^84^

**Scheme 38:**
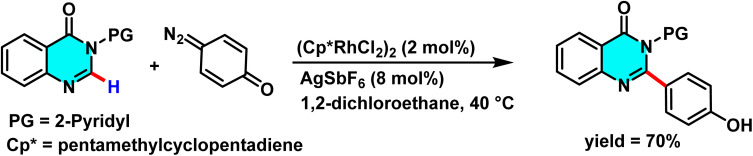
Rhodium(iii)-catalyzed selective C-6 arylation of pyrimidine in the presence of AgSbF_6_ as a co-catalyst.

In 2017, Das research group reported a novel method for the selective C-6 arylation of pyrimidine and other nitrogen-containing heterocycles, catalyzed by a Rh(iii) complex generated *in situ* from [CpRhCl_2_]_2_ in the presence of AgSbF_6_ as a co-catalyst, which abstracts chloride ions to form an active cationic species. The reaction utilizes quinone diazides as the arylating agent, with PivOH as an additive and DCE as the solvent, enhancing both yield and selectivity at the C-6 position of pyrimidine (Scheme 38). Studies revealed that a pyridyl directing group plays a critical role in guiding the reaction to the desired position and can be easily removed post-reaction.

**Scheme 39:**
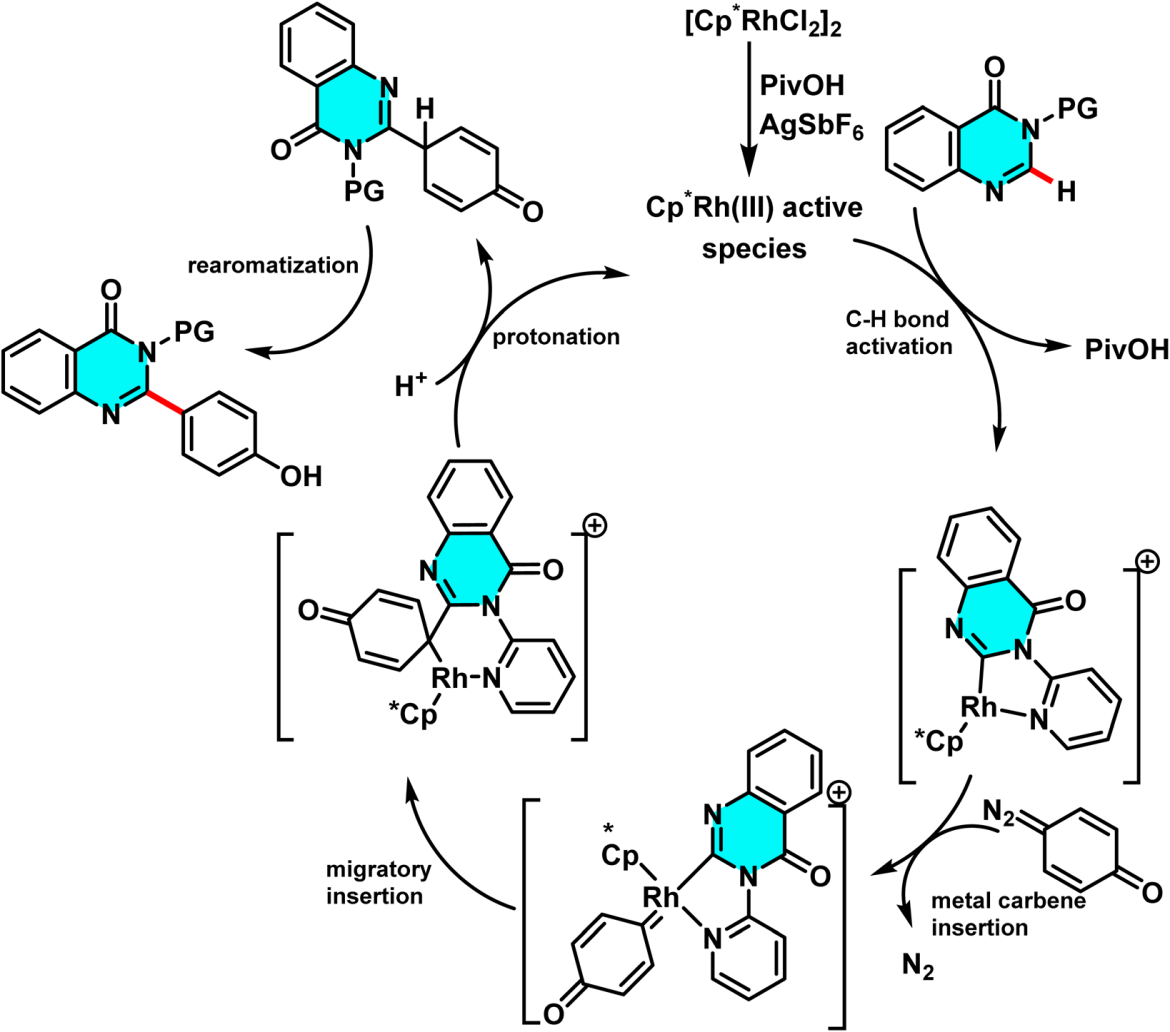
Plausible pathway for arylation of pyrimidine in the presence of AgSbF_6_ as a co-catalyst.

The reaction mechanism begins with the activation of the rhodium complex by AgSbF_6_, forming a cationic Rh(iii) species that coordinates with the directing group to activate the C(6)–H bond. Subsequently, the quinone diazide reacts with the metal species, releasing molecular nitrogen and generating a carbene–metal intermediate. Electron migration within the resulting six-membered intermediate leads to the formation of the C(6)-arylated pyrimidine product and rearomatization of the structure (Scheme 39).^85^

### 2.4 Other metal-catalyzed C–H activation strategies

Beyond widely utilized metals such as palladium, copper, and rhodium, other metals, including zinc, silver, and ruthenium, have contributed to C–H activation in heteroarenes, particularly pyrimidines. This section reviews a select number of studies that leverage these metals to develop diverse catalytic methodologies.

**Scheme 40:**
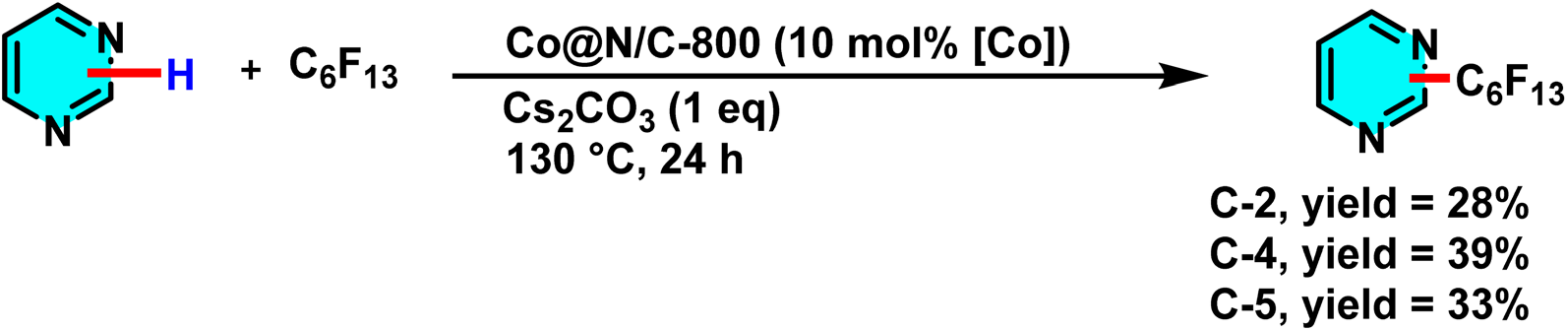
Direct perfluoroalkylation of pyrimidine using a cobalt-based nanocatalyst.

In 2020, Zhang *et al.* reported a new approach for direct perfluoroalkylation of pyrimidine and other nitrogenous heterocycles, using a cobalt-based nanocatalyst anchored on a nitrogen-doped carbon substrate (Co@N/C-800). Cobalt acetate (Co(OAc)_2_) was pyrolyzed at 800 °C with a phenanthroline (Phen) ligand and carbonaceous support to generate catalyst. The structural study revealed that including nitrogen into the carbon support significantly improves the stability and catalytic efficiency of cobalt nanoparticles by boosting electron transport and increasing catalyst durability (Scheme 40).

The mechanism proceeds through cobalt-catalyzed cleavage of the C–Br bond in perfluoroalkyl bromides, generating reactive perfluoroalkyl radicals. These radicals attack the activated C-4 and C-5 positions of pyrimidine to form a radical intermediate, which is then oxidized and rearomatized to yield the perfluoroalkylated product. Cs_2_CO_3_ serves as a base to suppress HBr accumulation and enhance efficiency. Comparative studies show that Co@N/C-800 exhibits superior activity and recyclability over Ni@N/C-800 and Mn@N/C-800.^86^

**Scheme 41:**
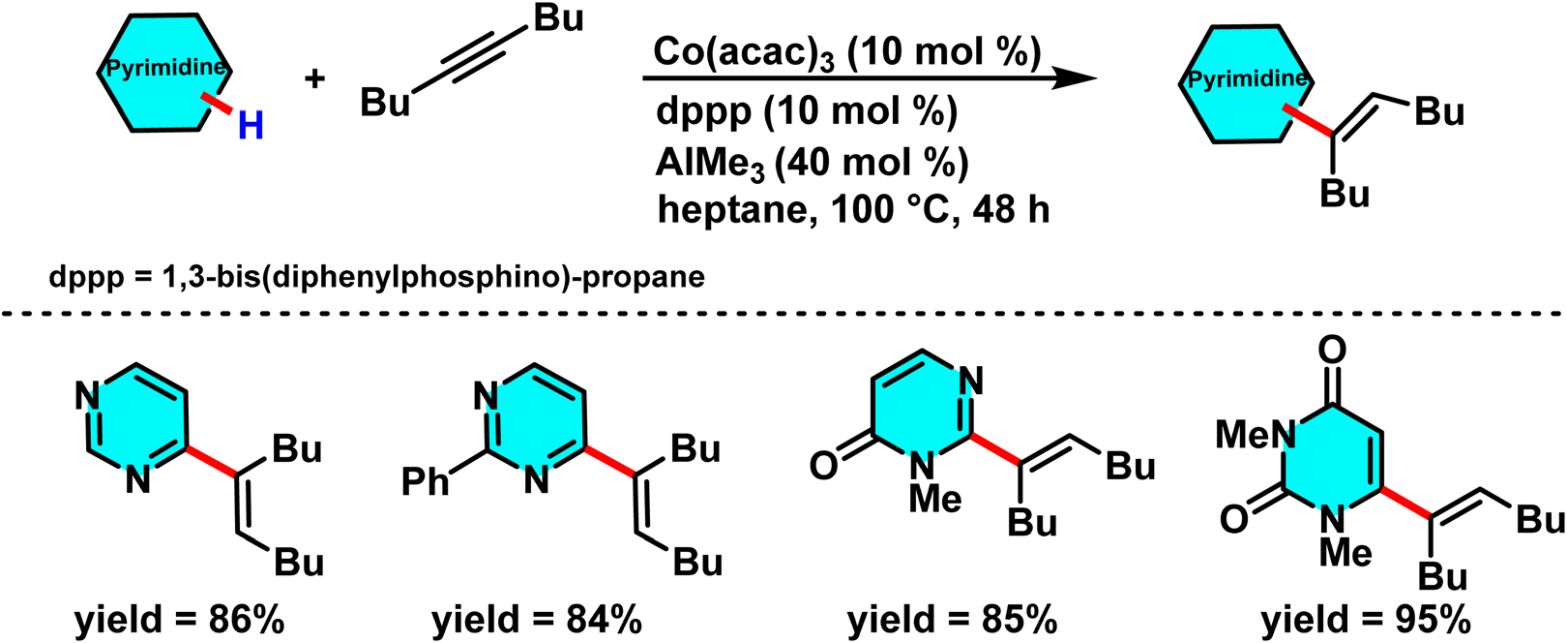
Co/Al-catalyzed alkenylation of pyrimidines derivative, affording the desired product in good yield.

In the other work, a new catalytic system based on cobalt was developed and synthesized. The catalyst is composed of a cobalt–diphosphine complex and trimethylaluminum (AlMe_3_) as a Lewis acid. In the supposed reaction mechanism, the generation of an intermediate complex between the substrate with the C–H bond, an alkyne, and the catalyst is proposed. The Lewis acid binds to this complex, which activates the C–H bond at the C-4 position of pyrimidine. The alkene binds to the complex, thus forming a new C–C bond between the alkyne and the C-4 position of pyrimidine (Scheme 41).

This cobalt-based catalytic system shows remarkable selectivity for C–H activation at the C-4 position of pyrimidine. The high regioselectivity arises from the diphosphine ligand incorporated in the catalyst and the optimized reaction conditions. The catalyst is easily prepared from accessible precursors, remains stable under reaction conditions, and delivers high yields with excellent site selectivity and product purity.^87^

**Scheme 42:**
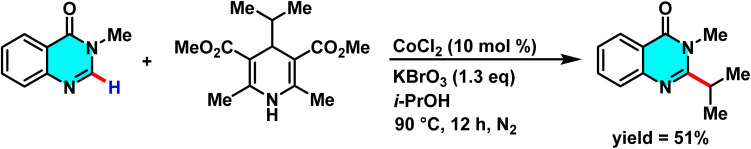
Direct alkylation of C–H bond in pyrimidines catalyzed by Co(ii).

In 2022, a new approach for direct alkylation of C–H bonds in nitrogen-containing heterocycles, especially pyrimidines, was introduced. This method utilizes cobalt(ii) as a catalyst and 1,4-dihydropyridines (DHPs) as an alkylating agent, enabling the incorporation of alkyl and acyl groups into heterocyclic frameworks. The reaction proceeds in the presence of potassium bromate (KBrO_3_) as an oxidant, which facilitates the generation of alkyl and acyl radicals from DHPs (Scheme 42). The reaction mechanism involves a SET between Co(ii) and DHPs, leading to the formation of highly reactive alkyl radicals. These radicals selectively react with the pyrimidine ring, followed by oxidation and rearomatization.^88^

**Scheme 43:**
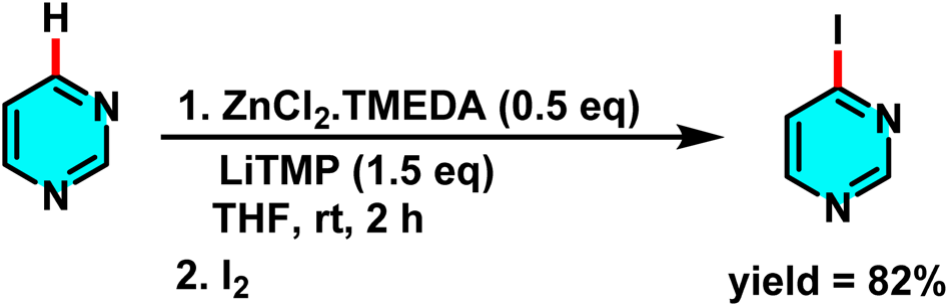
Deprotonation of pyrimidine with an *in situ* generated ZnCl_2_–TMEDA complex.

Seggio *et al.* developed a ZnCl_2_·TMEDA/LiTMP system for efficient zincation of pyrimidine and related N-heterocycles. Unlike conventional lithium-based protocols requiring low temperatures, this method achieves selective deprotonation at room temperature. ZnCl_2_·TMEDA provides the Zn^2+^ source, while LiTMP acts as a strong base to form an active Zn–Li complex that enables site-selective deprotonation at the C-4 and C-5 positions (Scheme 43). Additionally, studies have demonstrated that the presence of TMEDA or THF prevents the formation of side products such as dimers, thereby improving the efficiency of the process.

The mechanism begins with the interaction of ZnCl_2_·TMEDA and LiTMP to generate a stable zinc–amide complex. This complex deprotonates pyrimidine, forming a metalated intermediate that reacts with electrophiles such as I_2_ to afford the halogenated product.^89^

**Scheme 44:**
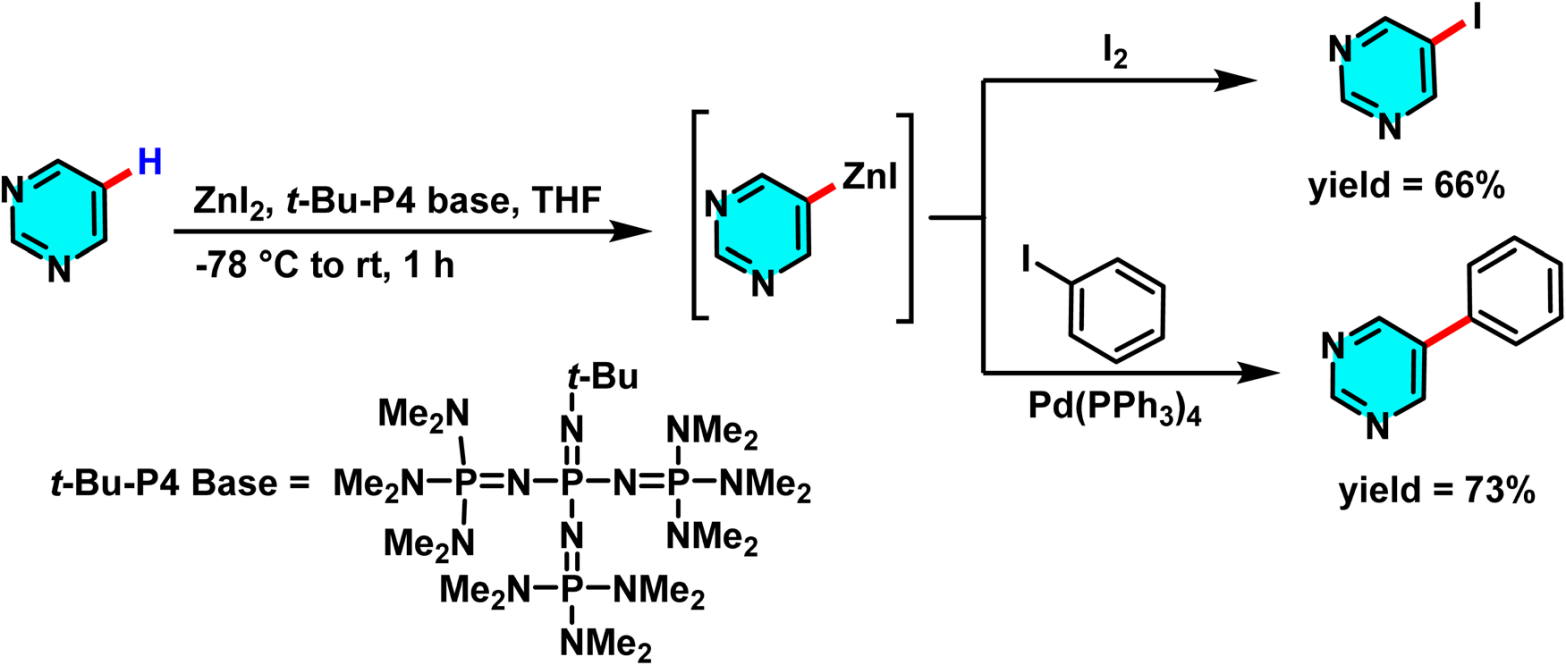
Sequential deprotonative zincation and electrophilic iodination of pyrimidine, leading to regioselective C–H functionalization.

In another study, a new approach for selective C–H bond activation in heteroaromatic compounds, especially at the C-5 position of pyrimidines, was developed. By utilizing the strong base *t*-Bu-P4 in combination with ZnI_2_ as a catalyst, the C–H bond at this specific position was efficiently and controllably converted into a C–Zn bond. This achievement represents a significant advancement in the development of new methodologies for the synthesis of complex organic molecules with broad applications in pharmaceuticals and materials science. Unlike traditional methods that employ lithium-based bases such as lithium tetramethylpiperidide (LiTMP), which exhibit lower reactivity at the C-5 position of pyrimidines, the use of *t*-Bu-P4 allowed for the selective deprotonation of the C-5 hydrogen. The resulting carbanion immediately reacted with Zn(ii) from ZnI_2_, leading to the formation of a stable pyrimidinyl zinc complex (Scheme 44).

The zinc-containing intermediate acts as a versatile precursor for further functionalization, particularly in cross-coupling reactions. The combination of *t*-Bu-P_4_ and ZnI_2_ forms an efficient and highly selective catalytic system for C–H activation in heteroaromatic compounds.^90^

**Scheme 45:**
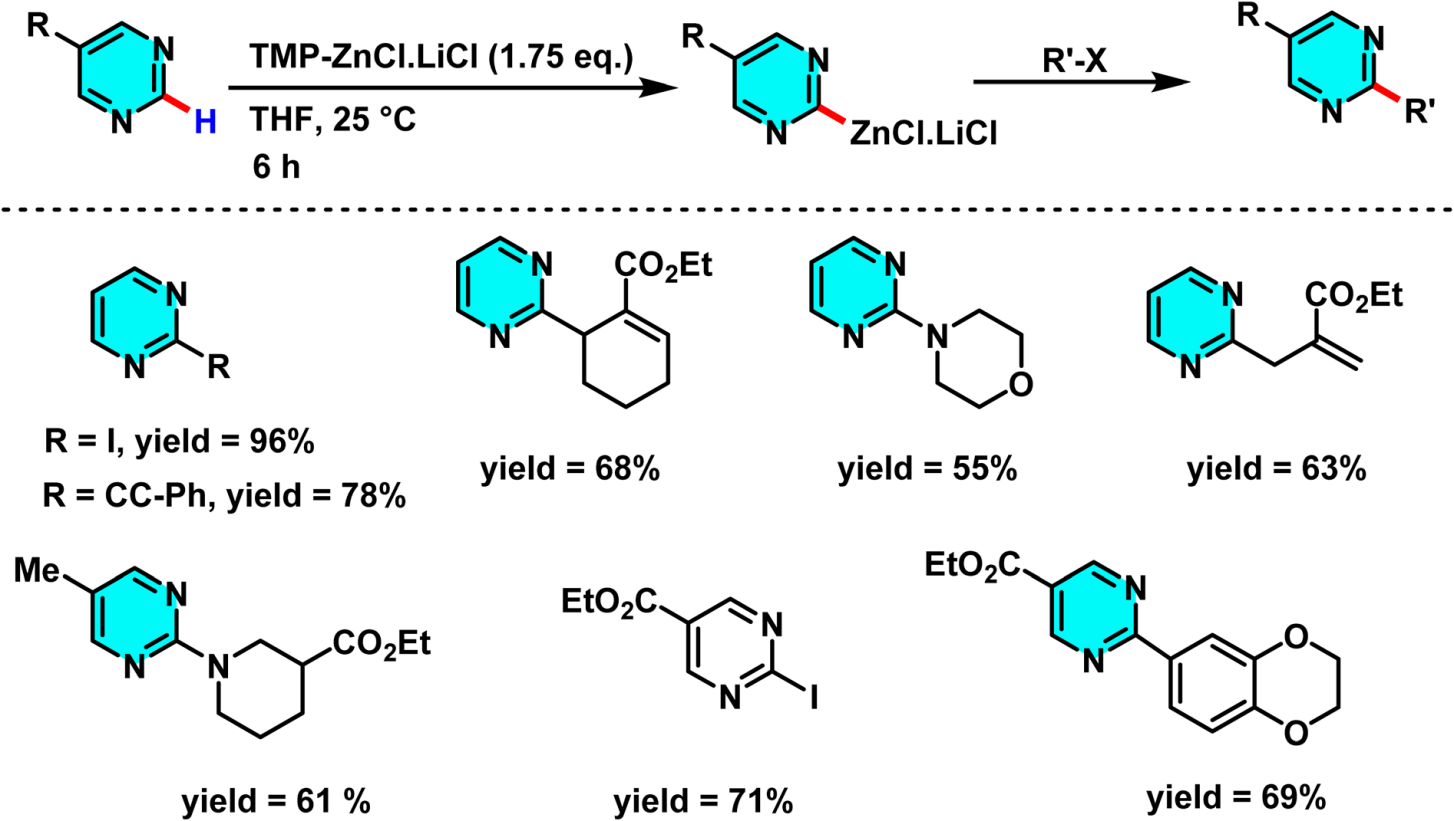
Regioselective zincation of pyrimidines using bimetallic bases TMP-ZnX·LiX and their application in various cross-coupling reactions.

Kremsmair *et al.* at in 2022 reported the regioselective zincation of pyrimidines using bimetallic bases TMP-ZnX·LiX (TMP = 2,2,6,6-tetramethylpiperidyl, X = Cl, Br) and their applications in various cross-coupling reactions. The reactions were conducted under mild conditions quenching with various electrophiles and affording the arylated products. A range of electron-rich and electron-poor substituted aryl halides containing sensitive functionalities including an ester, an amide, and a nitro group were well tolerated in these reactions (Scheme 45).

**Scheme 46:**
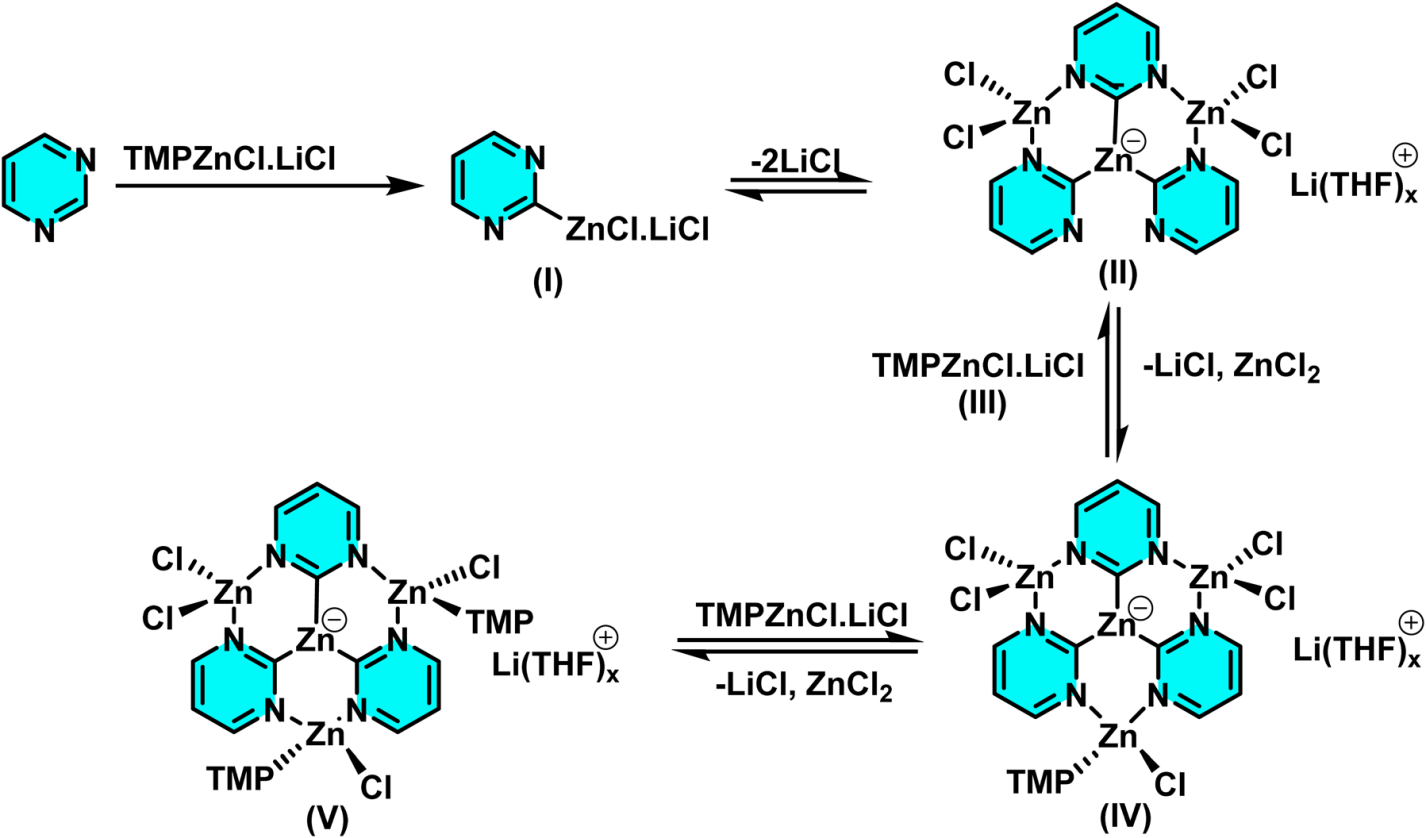
Plausible intermediates participating in the zincation of pyrimidine.

Bidentate ligands like 2,2′-bipyridine significantly reduce the yield of zincated pyrimidine (I) by competing with the pyrimidine for coordination with TMPZnCl·LiCl (III) (Scheme 46). The need for an excess base (III) arises from its partial consumption in side reactions with intermediates like complex (II), forming species such as (IV) and (V). The unique regioselectivity is facilitated by the coordination of the substrate to the organometallic reagent and the formation of thermally stable zincated intermediates.^91^

**Scheme 47:**
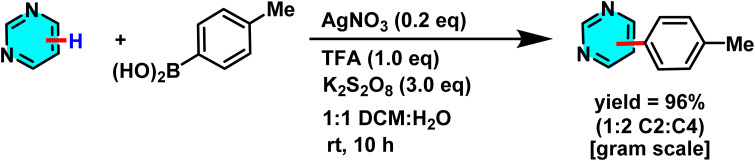
Direct arylation of pyrimidines using arylboronic acids.

In another study disclosed a new approach to the direct arylation of electron-deficient heterocycle using arylboronic acids. The reaction was conducted under mild conditions at room temperature, successfully performing the arylation of pyrimidines at its active sites. Silver nitrate (AgNO_3_) served as catalyst, and potassium persulfate (K_2_S_2_O_8_) was employed as the co-oxidant. Besides, trifluoracetic acid (TFA) was also added to protonate the pyrimidine to make it more reactive. A biphasic solvent system of water/dichloromethane was used, wherein water was important to stabilize silver nitrate and potassium persulfate (Scheme 47).

**Scheme 48:**
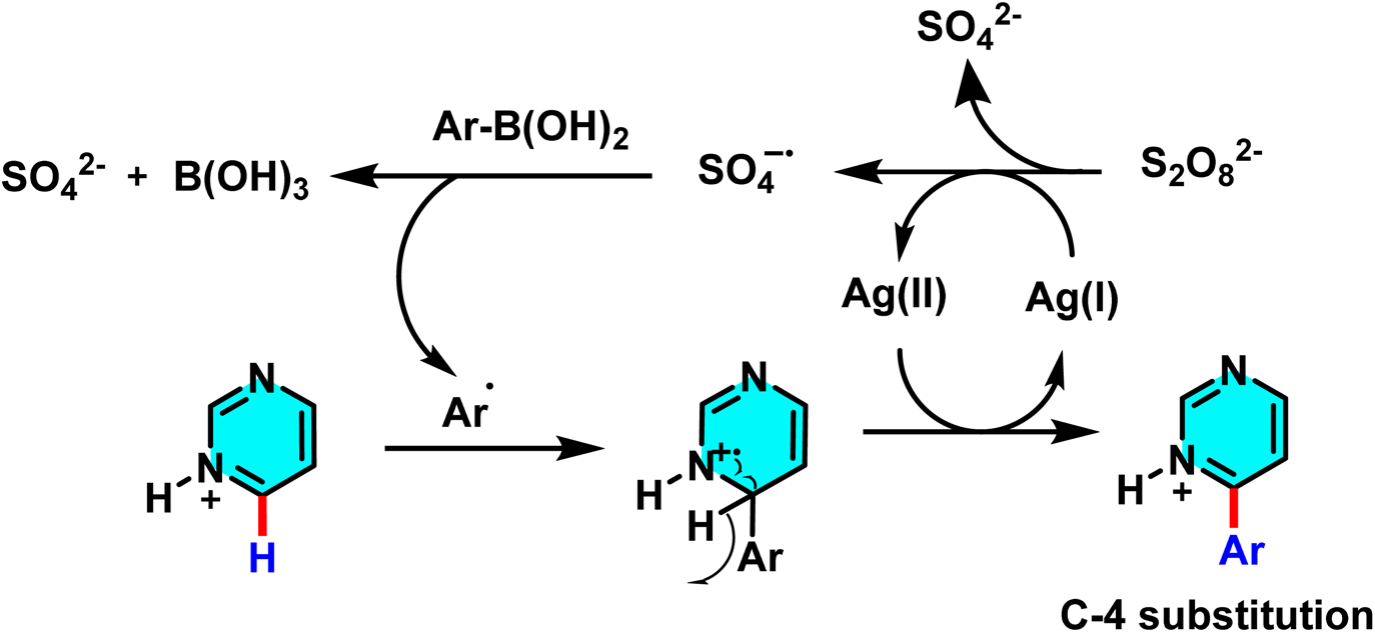
Suggested mechanism for arylation of pyrimidines *via* the Minisci pathway.

The proposed reaction mechanism starts with the oxidation of persulfate to form sulfate radical anions (SO_4_˙^−^) by Ag(i). The sulfate radicals, which are potent oxidizing agents, oxidize the arylboronic acid to form an aryl radical. The aryl radical then attacks the protonated pyrimidine to form a heterocyclic radical intermediate, which is then oxidized by Ag(ii) to form the final arylated product (Scheme 48).^92^

**Scheme 49:**
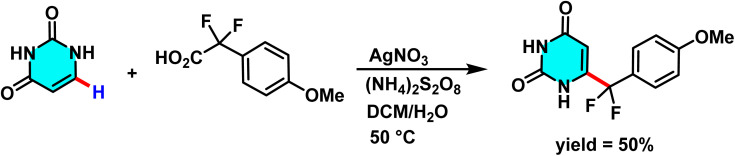
Silver nitrate (AgNO_3_)-catalyzed difluoromethylation of C–H bonds in pyrimidines.

In 2020, a study explored difluoromethylation of C–H bonds in N-heteroarenes, including pyrimidines. As electron-deficient heteroarenes, pyrimidines exhibit limited reactivity in classical electrophilic aromatic substitution reactions, such as the Friedel–Crafts reaction. However, radical-based approaches, such as the Minisci reaction, provide an alternative strategy for the direct functionalization of these compounds. In this study, silver nitrate (AgNO_3_) was employed as a catalyst, while ammonium persulfate ((NH_4_)_2_S_2_O_8_) served as the oxidant, with a biphasic dichloromethane/water system being utilized. The aqueous phase facilitates catalyst oxidation, whereas the organic phase enables efficient difluoromethyl radical transfer to pyrimidine (Scheme 49).

**Scheme 50:**
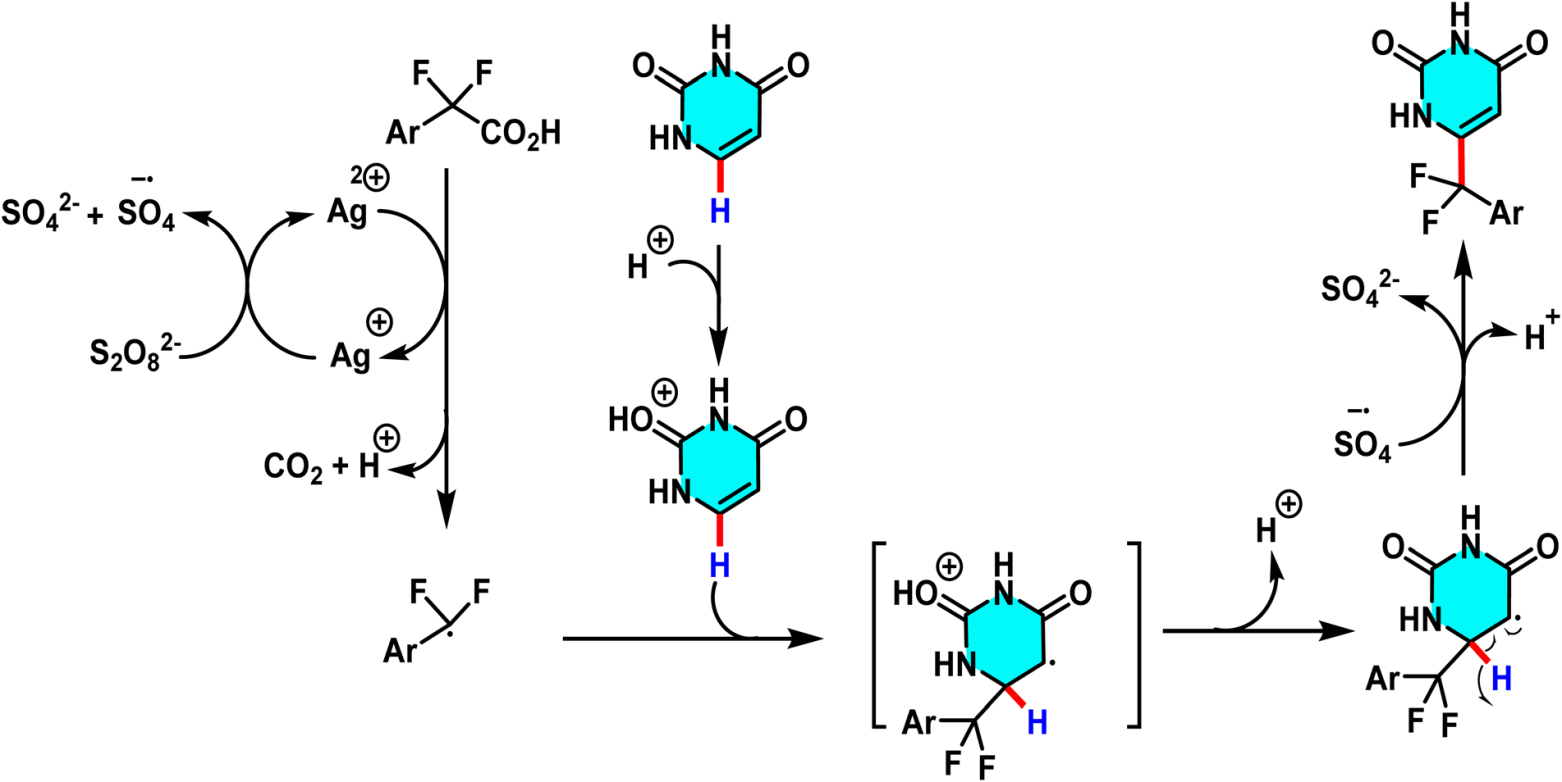
Suggested pathway for difluoromethylation of pyrimidines.

The proposed reaction mechanism begins with the oxidation of Ag(i) to Ag(ii) by ammonium persulfate. Subsequently, Ag(ii) abstracts an electron from aryl difluoroacetic acid, generating a nucleophilic difluoromethyl radical (˙CF_2_Ar) along with the release of CO_2_. This CF_2_ radical then attacks protonated pyrimidine, forming a cyclohexadienyl radical intermediate. The final difluoromethylated product is obtained after the deprotonation and oxidation *via* a single-electron transfer process (Scheme 50). The CF_2_ moiety is highly significant in pharmaceuticals, agrochemicals, and materials science due to its stability, isosteric similarity to ether or carbonyl oxygen, and ability to form hydrogen bonds.^93^

**Scheme 51:**
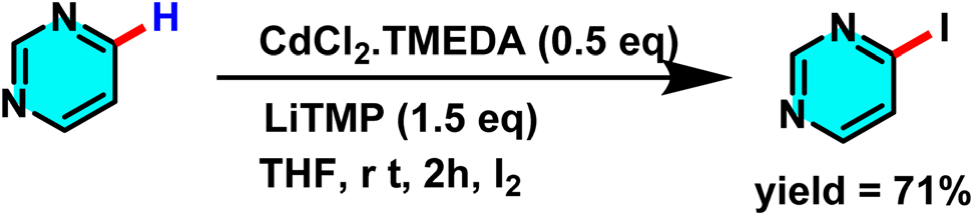
Deprotonation of pyrimidines with CdCl_2_ and subsequent iodination.

The Mongin group studied a mechanism involving deprotonation of heterocycles followed by electrophilic iodination. They used a bimetallic superbase of CdCl_2_·TMEDA (Lewis acid cadmium source) and LiTMP (Brønsted base) (Scheme 51). CdCl_2_·TMEDA and LiTMP interacted to form a reactive organometallic species, with cadmium as the coordination center and lithium stabilizing the anion. This complex abstracted a proton from pyrimidine, forming a stabilized pyrimidine anion. The anion then underwent electrophilic substitution with I_2_ to yield the iodinated product with a C–I bond.^94^

**Scheme 52:**
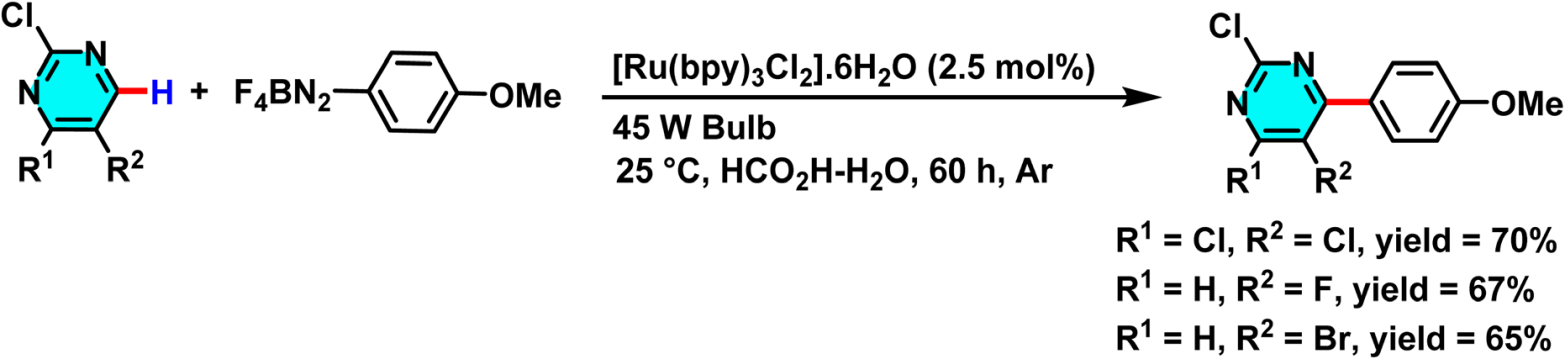
Photoredox-catalyzed radical arylation of pyrimidines in aqueous formic acid.

In 2014, Xue *et al.* reported a novel and sustainable method for the direct C–H arylation of pyrimidine and other nitrogen-containing heterocycles, leveraging photoredox catalysis and aryl diazonium salts. The reaction employs [Ru(bpy)_3_]Cl_2_·6H_2_O as a photocatalyst and visible light as the energy source, where a single electron transfer process reduces the aryl diazonium salt, generating an aryl radical. This radical subsequently attacks the pyrimidine, and following rearomatization, yields the arylated product. The use of water as a solvent not only enhances reaction sustainability but also minimizes chemical waste and improves process efficiency (Scheme 52).

**Scheme 53:**
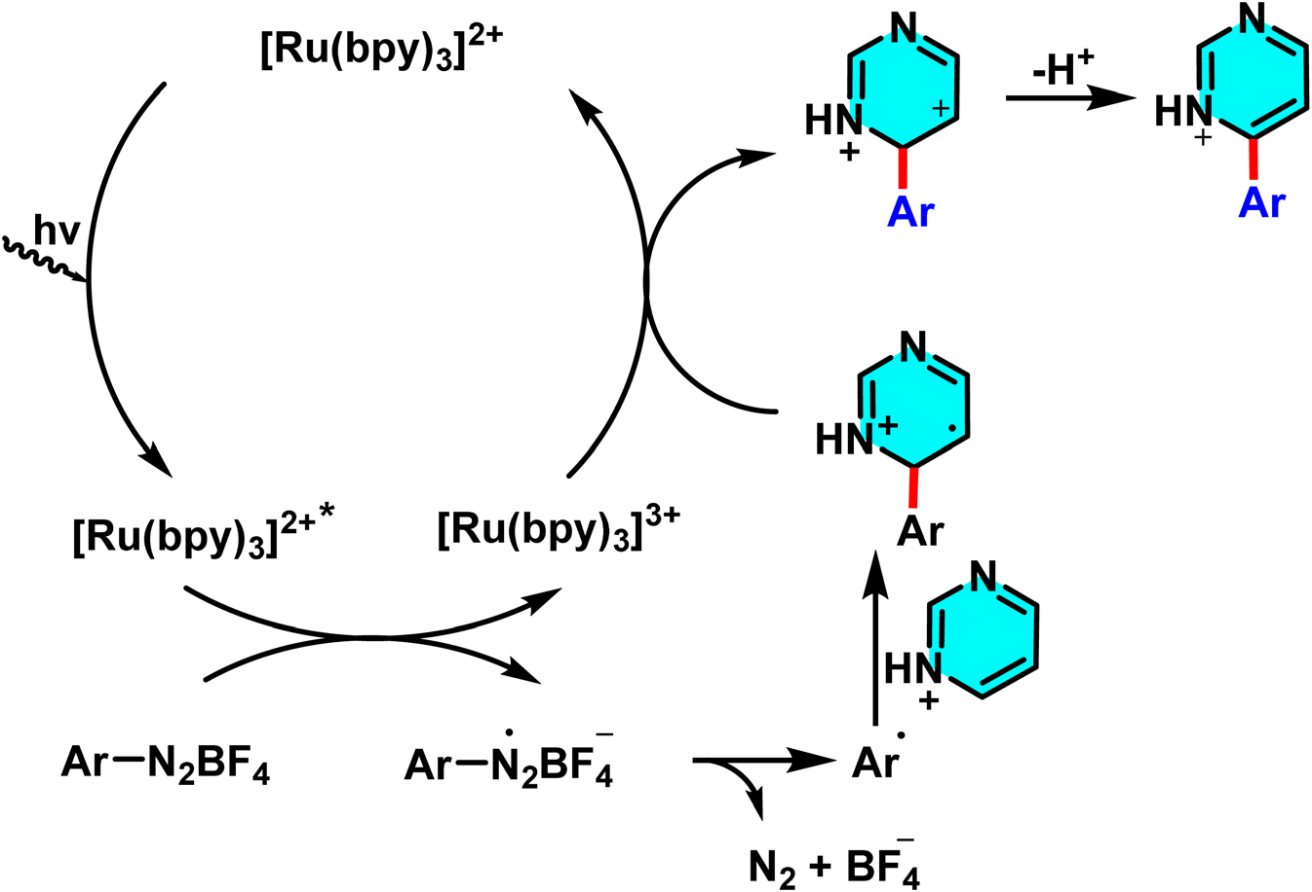
Suggested mechanistic pathway for the photoredox-catalyzed radical arylation of pyrimidines.

The reaction mechanism involves the visible light-excitation of the ruthenium photocatalyst, followed by electron transfer to the aryl diazonium salt to produce an aryl radical. This radical then adds to the pyrimidine, forming a radical intermediate, which undergoes oxidation and rearomatization to afford the final product. By eliminating the need for expensive transition metals such as palladium or nickel, minimizing the use of organic solvents, and demonstrating broad compatibility with diverse aryl diazonium salts, this approach provides a practical and environmentally friendly strategy for C–H arylation of electron-deficient heteroarenes (Scheme 53).^95^

**Scheme 54:**
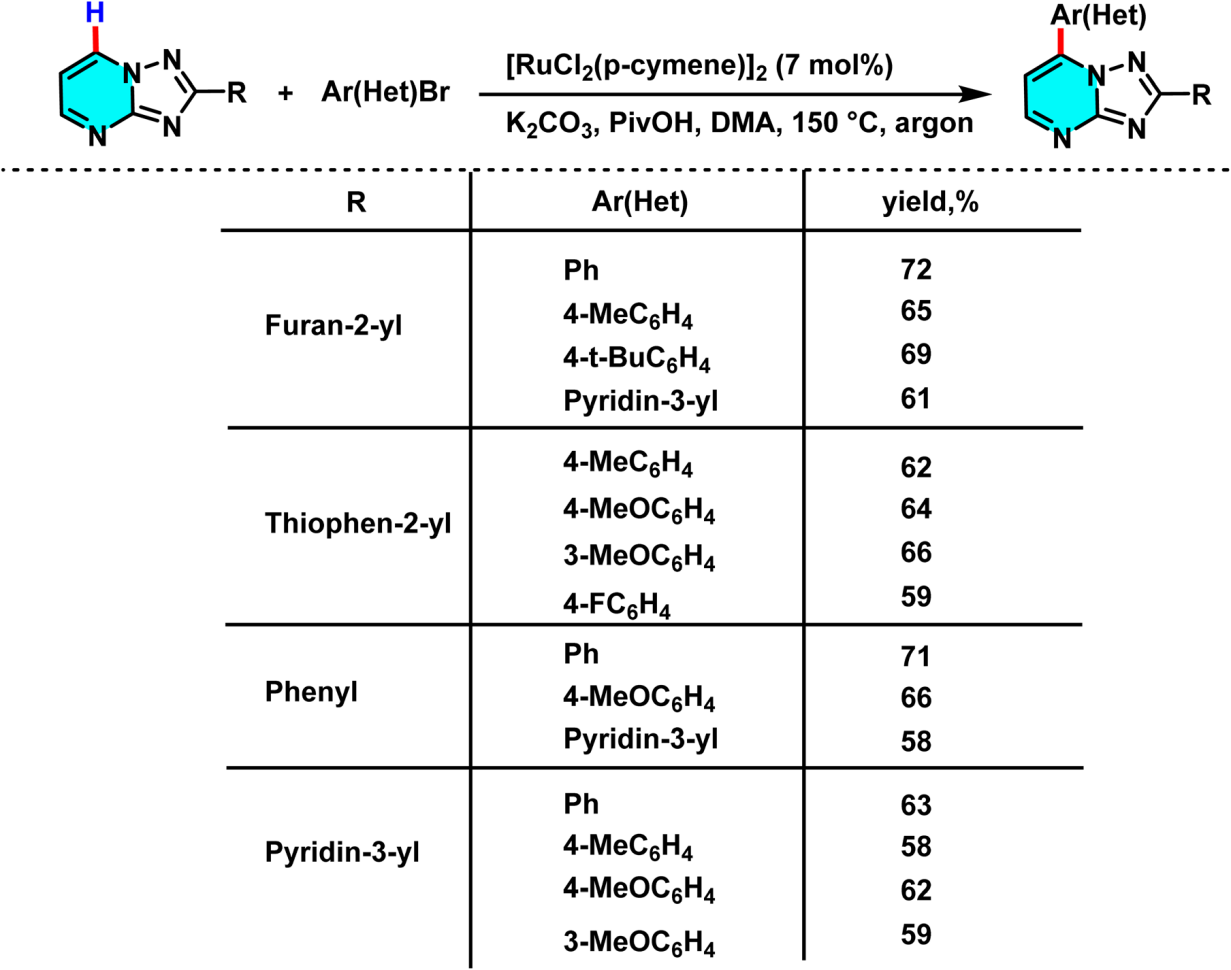
Targeted C–H arylation at the C-7 position of 2-(het)aryl[1,2,4]triazolo[1,5-*a*]pyrimidines using a Ru(ii) catalyst with aryl halides.

In 2024, Shepelenko and co-workers introduced an innovative method for the selective C–H arylation at the C-7 position of 2-(het)aryl[1,2,4]triazolo[1,5-*a*]pyrimidines, utilizing a Ru(ii) catalyst and aryl halides. Unlike conventional arylation pathways, which are typically limited to C–H activation at α-positions relative to the aryl group, this method achieves selective C-7 activation without requiring directing groups and under milder conditions. The catalytic system comprises [RuCl_2_(*p*-cymene)]_2_ as the catalyst, PivOH as a ligand, and K_2_CO_3_ as a base, performed in *N*,*N*-dimethylacetamide (DMA) solvent at 150 °C, yielding high amounts of arylated products (Scheme 54).

The reaction proceeds through a CMD-type C–H activation pathway. The Ru(ii) catalyst forms an active complex that coordinates with the heterocycle and generates a Ru–aryl intermediate, which couples with an aryl halide to form a C–C bond at the C-7 position. This route affords higher C-7 selectivity and yields than Pd-based methods, while PivOH stabilizes the Ru complex and facilitates C–H activation.^96^

**Scheme 55:**
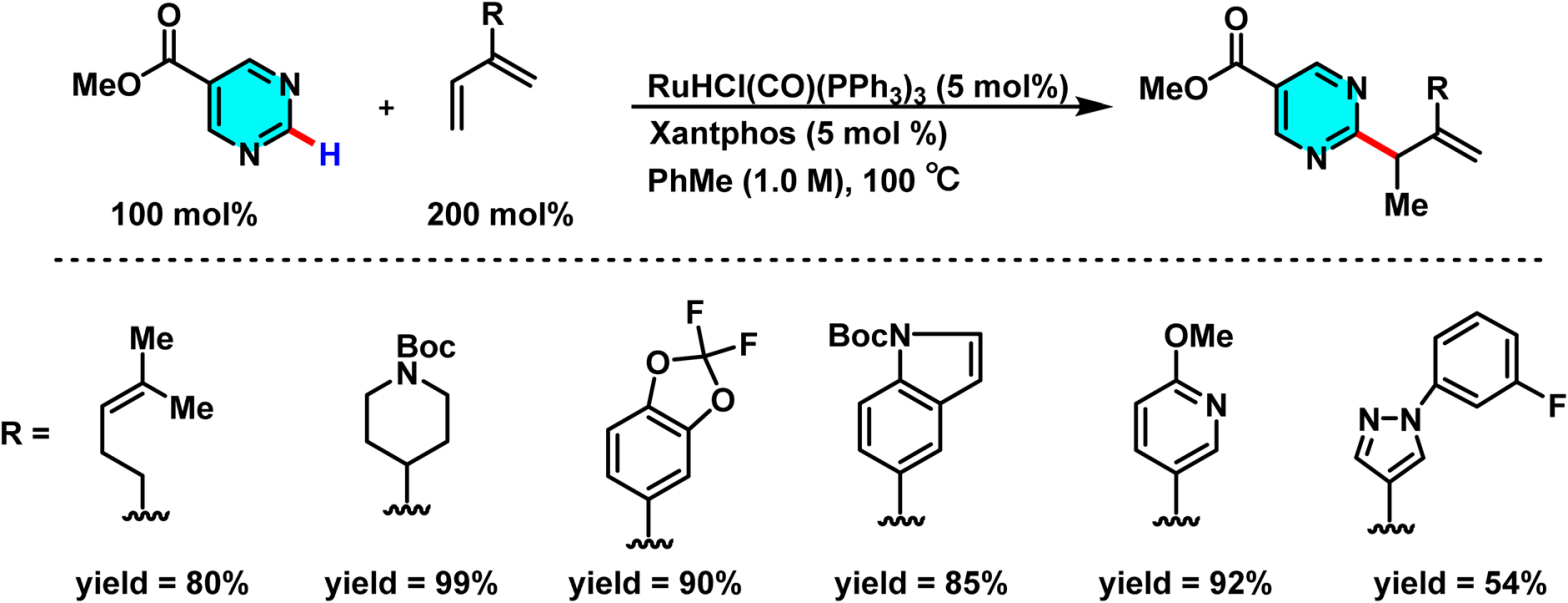
Ruthenium-catalyzed dearomative hydrofunctionalization of methyl pyrimidine-5-carboxylate with dienes *via* hydrogen auto-transfer strategy.

A notable advancement in branch-selective C–H functionalization of pyrimidines has been achieved through a ruthenium-catalyzed dearomative addition-hydrogen autotransfer (DA-HAT) protocol, recently reported by Shezaf and co-workers (2025). This strategy provides an efficient and selective route for C–C bond formation across a variety of N-heteroaromatics, including pyrimidines. The optimal catalytic system is based on RuHCl(CO)(PPh_3_)_3_ (5 mol%) in combination with Xantphos (5 mol%), which afforded high yields up to 93% in model substrates. Alternative ruthenium precursors, such as [RuCl_2_(cymene)]_2_, displayed significantly lower activity under comparable conditions. The method features high chemoselectivity and broad substrate compatibility, enabling direct late-stage functionalization of complex molecules such as voriconazole (Scheme 55). Although C(2)-selective functionalization is common in pyrimidines, regioselectivity in this system is dictated by the LUMO distribution of the heteroarene, leading to C-4 or C-6 alkylation depending on substrate electronics. The catalytic cycle proceeds *via* hydroruthenation of the diene to generate an allylruthenium nucleophile, which adds to the heteroaryl ring through a dearomative pathway. β-Hydride elimination regenerates Ru and affords the branched product. DFT studies confirm that the dearomative addition is the turnover-limiting step. This Ru-catalyzed DA-HAT platform thus provides a unique, efficient, and regioselective route to heteroaryl functionalization.^97^

**Scheme 56:**
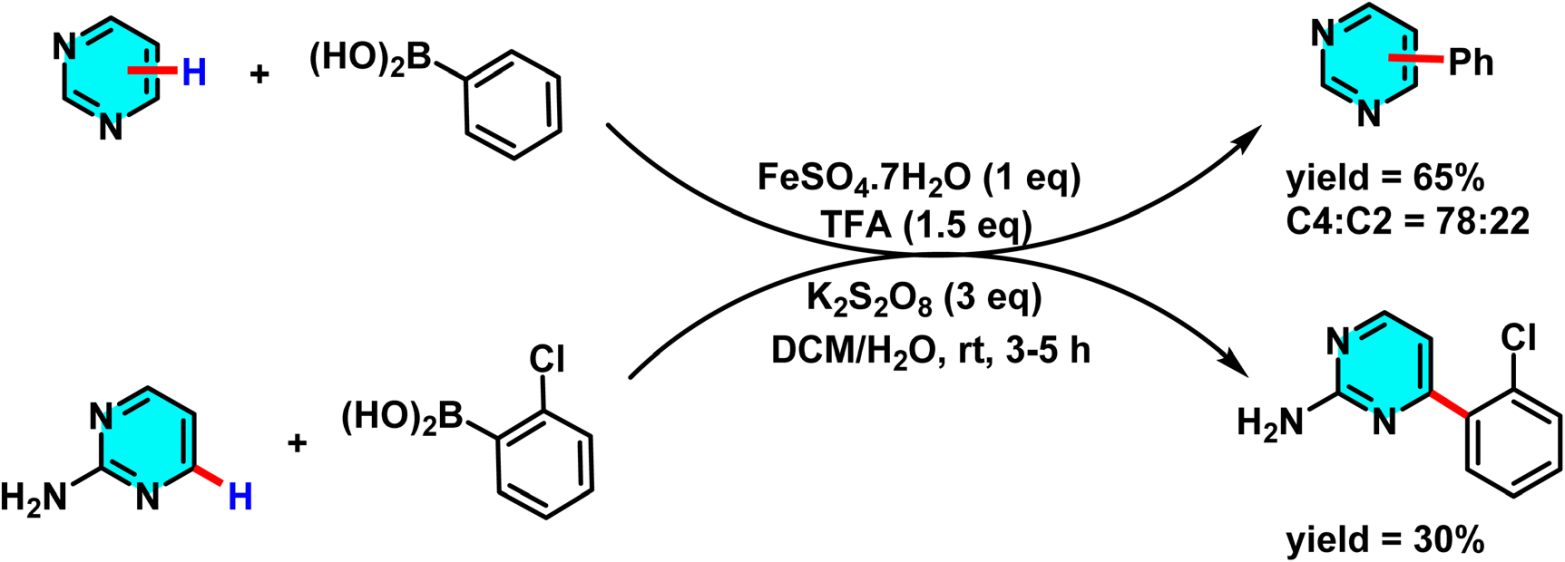
Direct arylation of pyrimidine using FeSO_4_·7H_2_O.

**Scheme 57:**
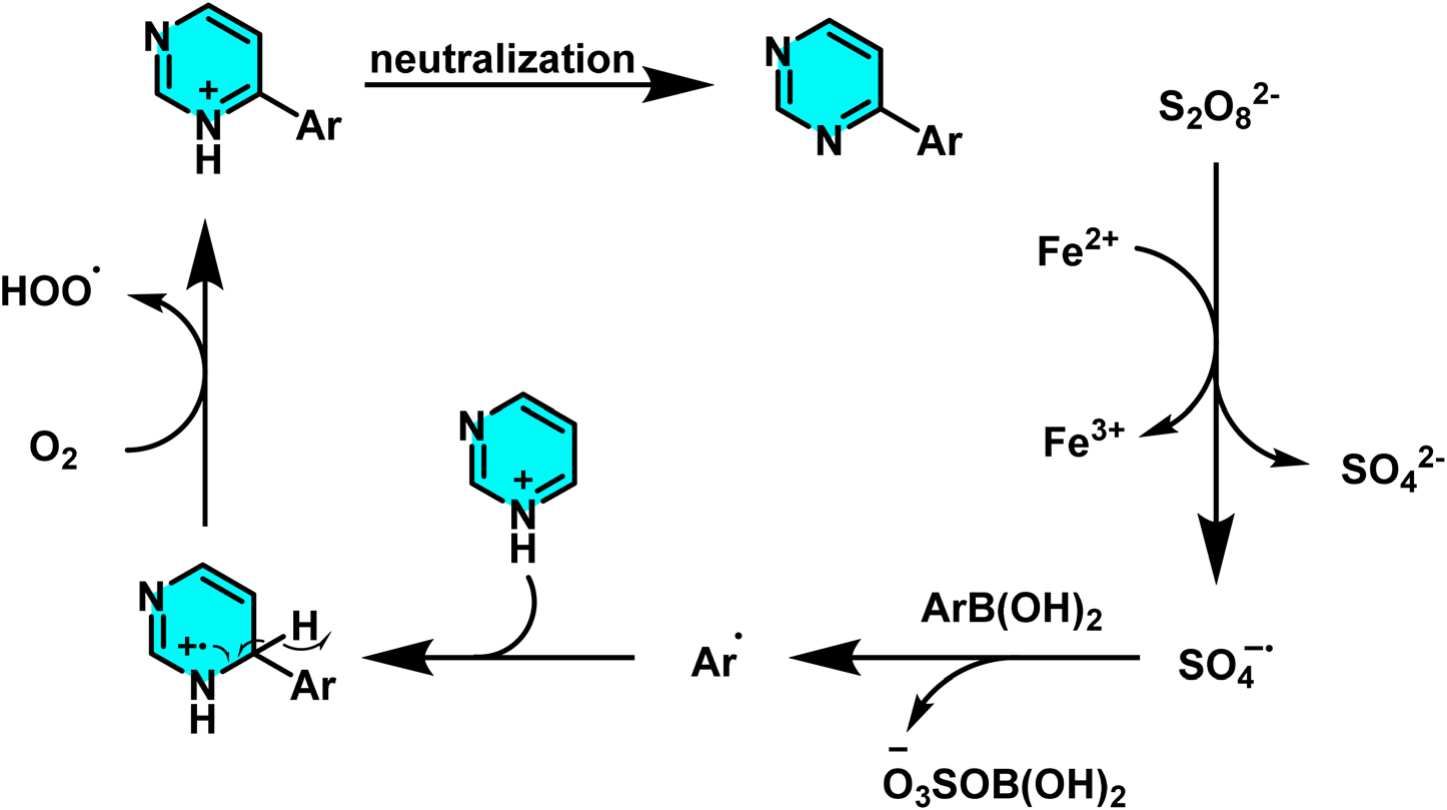
Proposed mechanism for the iron-catalyzed direct arylation of pyrimidines using arylboronic acids.

In a separate study, Komeyama *et al.* employed Fe(ii) sulfate heptahydrate (FeSO_4_·7H_2_O) as the primary reagent for the direct arylation of heteroarenes with other aromatic compounds. The key role of iron sulfate lies in its ability to generate radical species that facilitate the reaction (Scheme 56). In this process, iron ions react with K_2_S_2_O_8_ to produce sulfate radicals (SO_4_^−^˙). These radicals subsequently attack boronic acids, generating aryl radicals (Ar˙), which then couple with the heteroarene to achieve direct arylation. To enhance reactivity, TFA is utilized as a protonating agent for the heteroarenes (Scheme 57). Protonation reduces the electron density on the heteroaromatic ring, thereby increasing its susceptibility to attack by the aryl radical.^98^

**Scheme 58:**
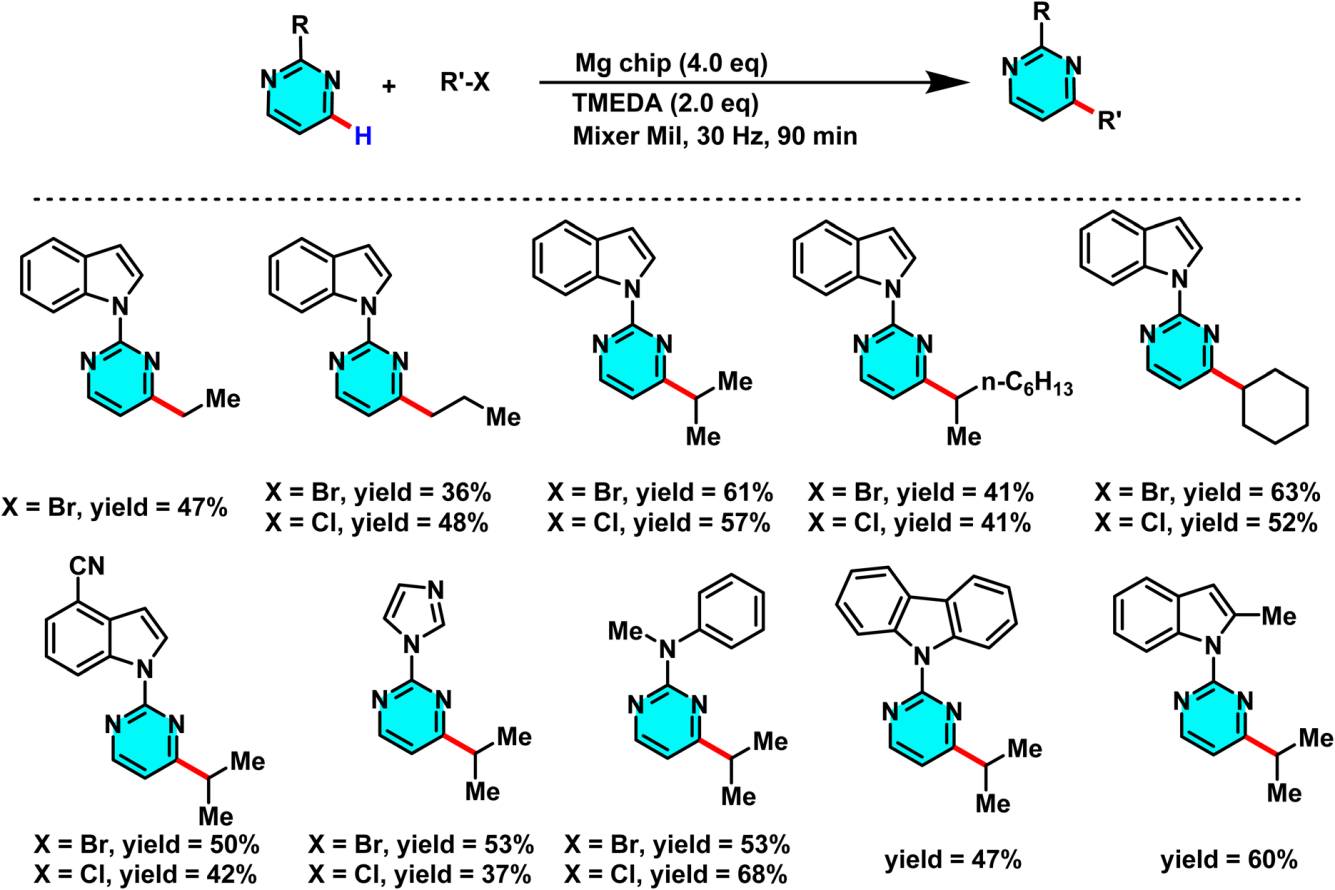
Mechanochemical Minisci reaction of pyrimidine derivatives with alkyl bromides and chlorides mediated by magnesium.

In 2021, Wu *et al.* developed a mechanochemical C–H alkylation of pyrimidines using magnesium chips and alkyl halides (bromides and chlorides). TMEDA served as a critical additive to boost efficiency and selectivity. Mechanical activation induced homolytic C–X bond cleavage, generating alkyl radicals (R˙) from primary, secondary, tertiary, and even unreactive chlorides. Pyrimidines acted as radical acceptors, yielding 4-alkylated products selectively. TMEDA facilitated Mg–halide interactions. The solvent-free process avoided inert gas and used chips (not powder) to prevent Grignard formation (Scheme 58).

The reaction proceeds through a radical chain process initiated by mechanical milling. Magnesium activates the alkyl halide *via* homolytic C–X cleavage, generating an alkyl radical that adds to the C-4 position of pyrimidine. Radical clock and TEMPO-inhibition experiments confirm this pathway, which differs from Grignard-type mechanisms. Mechanical milling also removes the MgO layer, enabling the use of less reactive alkyl chlorides in Minisci-type alkylation.^99^

**Scheme 59:**
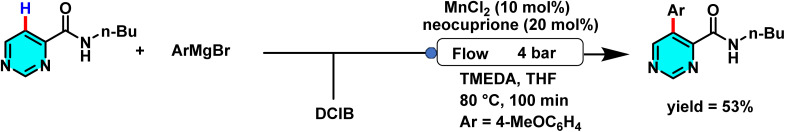
C-5 selective C–H arylation of pyrimidine catalyzed by Mn(ii).

In 2018, Zhu *et al.* showed a new manganese-catalyzed method for selective C–H arylation of pyrimidine under continuous flow conditions. This approach was introduced as an efficient and cost-effective alternative to conventional systems relying on precious metals such as palladium and ruthenium. In this study, MnCl_2_ was employed as the catalyst, with TMEDA and 2,9-dimethyl-1,10-phenanthroline (neocuproine) serving as ligands, and 1,2-dichloro-2-methylpropane (DCIB) as the oxidant, resulting in enhanced reaction yield and selectivity. The ligands played a crucial role in stabilizing the manganese complex and preventing its agglomeration and significantly improved the catalytic activity. Additionally, the use of THF as a solvent facilitated the creation of a suitable reaction environment and enabled precise process control within the continuous flow system (Scheme 59).

The mechanism proceeds through a SET process coupled with ligand-to-ligand hydrogen transfer (LLHT), enabling C–H activation and formation of a Mn–aryl intermediate. This species reacts with an aromatic substrate in the presence of an oxidant to afford the arylated product. DFT studies indicate that this pathway has a lower activation barrier than classical oxidative addition–reductive elimination, explaining its higher efficiency.^100^

**Scheme 60:**
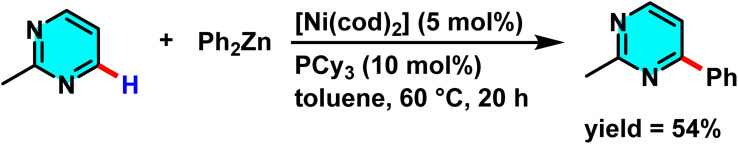
Nickel-catalyzed phenylation of pyrimidines with Ni catalyst.

Hyodo and co-authors introduced a new method for selective C–H arylation of pyrimidine and other nitrogen-containing heterocycles using a nickel catalyst and organozinc reagents. Unlike conventional palladium-based methods, which typically require aryl halides and strong oxidants, this approach utilizes an oxidative nucleophilic pathway for arylation. In this reaction, the [Ni(cod)_2_]/PCy_3_ complex serves as the catalyst, while an organozinc compound (ArZnX) acts as both the aryl donor and an internal oxidant to enhance the yield and provide the improved control over regioselectivity at specific positions on the pyrimidine ring. Compared to palladium-based systems, the nickel catalyst demonstrates greater compatibility with diverse functional groups and offers enhanced flexibility in targeting reactive sites (Scheme 60).

**Scheme 61:**
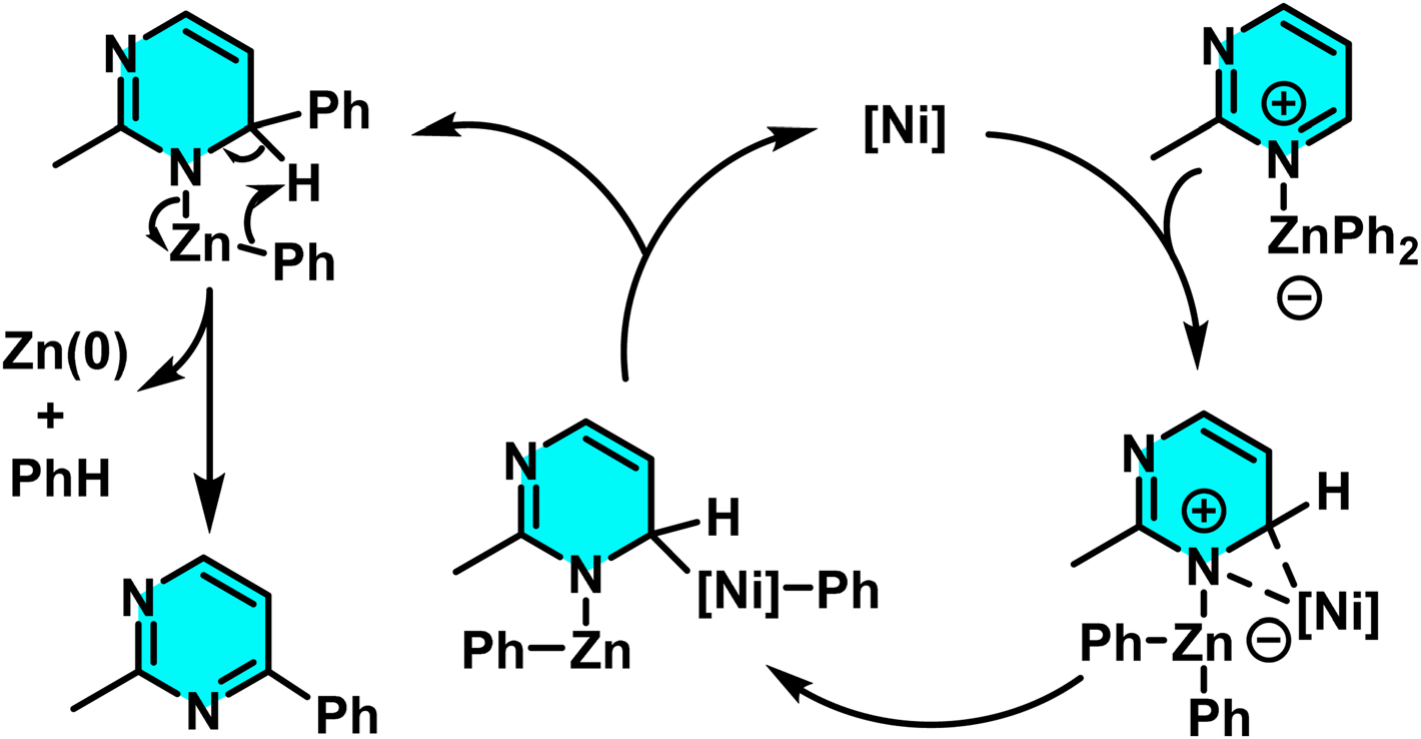
Proposed catalytic cycle for nickel-mediated phenylation of pyrimidines.

The reaction mechanism involves the formation of an active nickel(0) complex from Ni(cod)_2_ and the PCy_3_ ligand, followed by transmetalation of the aryl group from the organozinc compound to nickel, generating a nickel-aryl species. This species then attacks the pyrimidine, forming a 1,2-dihydropyrimidine intermediate. Subsequently, the zinc species, acting as an internal oxidant, facilitates rearomatization, yielding the final arylated product (Scheme 61).^101^

### 2.5 Critical analysis for transition-metal-catalyzed C–H activation

Transition-metal-catalyzed C–H activation in pyrimidines most notably using palladium, nickel, and copper catalysts as emerged as a powerful approach for achieving high levels of regioselectivity, exemplified by the C(5)-arylation of 2-aminopyrimidines and C(7)–H activation in pyrazolo[1,5-*a*]pyrimidines. These transformations provide efficient access to functionalized heterocycles of significant pharmaceutical relevance, enabling streamlined synthesis of bioactive molecules. Despite these advantages, several challenges constrain broader applicability. The reliance on precious metals such as palladium increases cost and raises concerns over toxicity and environmental burden, while the need for specific directing groups can limit substrate scope and functional-group tolerance. Moreover, the generation of metal-containing waste poses sustainability issues, particularly in large-scale operations.

Future progress will depend on the development of catalysts based on earth-abundant metals such as nickel or copper, as well as the implementation of recyclable catalytic systems to minimize environmental impact. The integration of computational modeling to predict catalyst–substrate interactions and transition-state energetics offers a promising strategy to further refine regioselectivity and expand substrate diversity. Such advances could transform transition-metal-catalyzed C–H activation from a highly selective laboratory tool into a robust, scalable, and environmentally responsible methodology for the synthesis of medicinal agents and functional materials.

## 3 Metal-free strategies for direct C–H activation of pyrimidines and related heteroarenes

The metal-free activation of C–H bonds in heteroarenes, particularly pyrimidines, has garnered significant attention due to its simplicity, stability, and alignment with green chemistry principles. This section reviews metal-free strategies, encompassing photochemical and oxidative approaches, for such transformations. These methods offer efficient alternatives for synthesizing bioactive compounds.

**Scheme 62:**
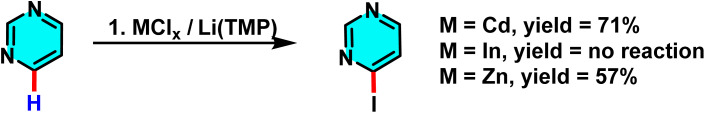
Metalation of pyrimidines with various mixtures and subsequent trapping with I_2_.

The Mongin research group successfully developed a new cadmium-based strong base. This base exhibits a unique ability to abstract hydrogen atoms from aromatic and heteroaromatic rings, particularly pyrimidines, under ambient conditions in tetrahydrofuran. The base was synthesized through the combination of cadmium chloride, TMEDA, and a lithium compound (Scheme 62).

**Scheme 63:**
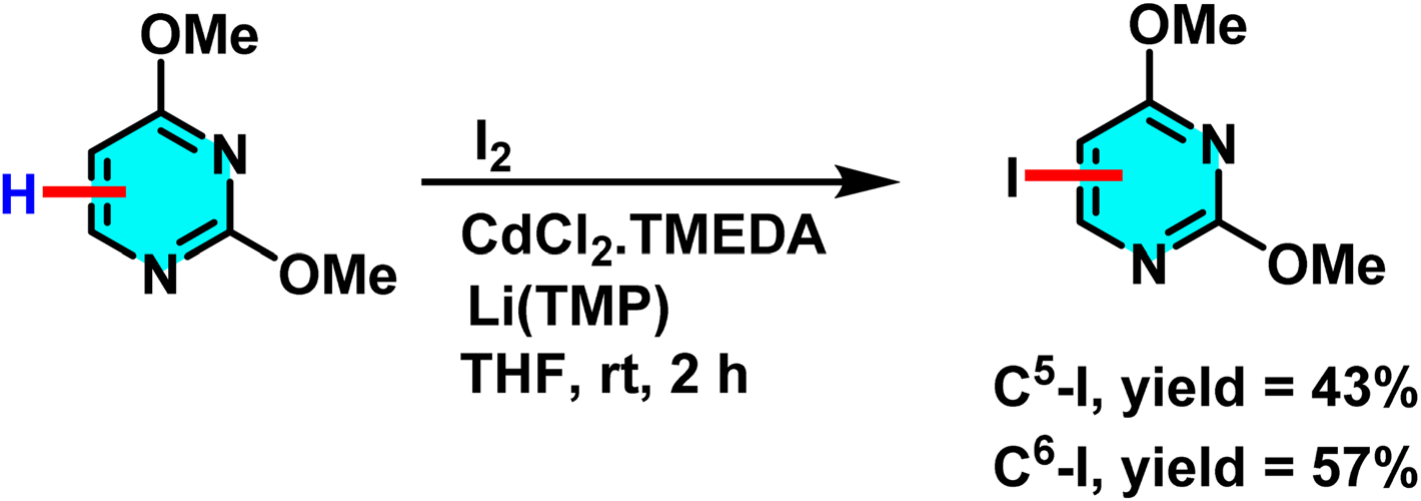
Metalation of pyrimidines with CdCl_2_·TMEDA and subsequent trapping with I_2_.

A prominent feature of this base is its high selectivity, which enables the precise abstraction of hydrogen from various compounds. In comparison to zinc and indium metals, cadmium demonstrated superior performance in this context (Scheme 63).

**Scheme 64:**
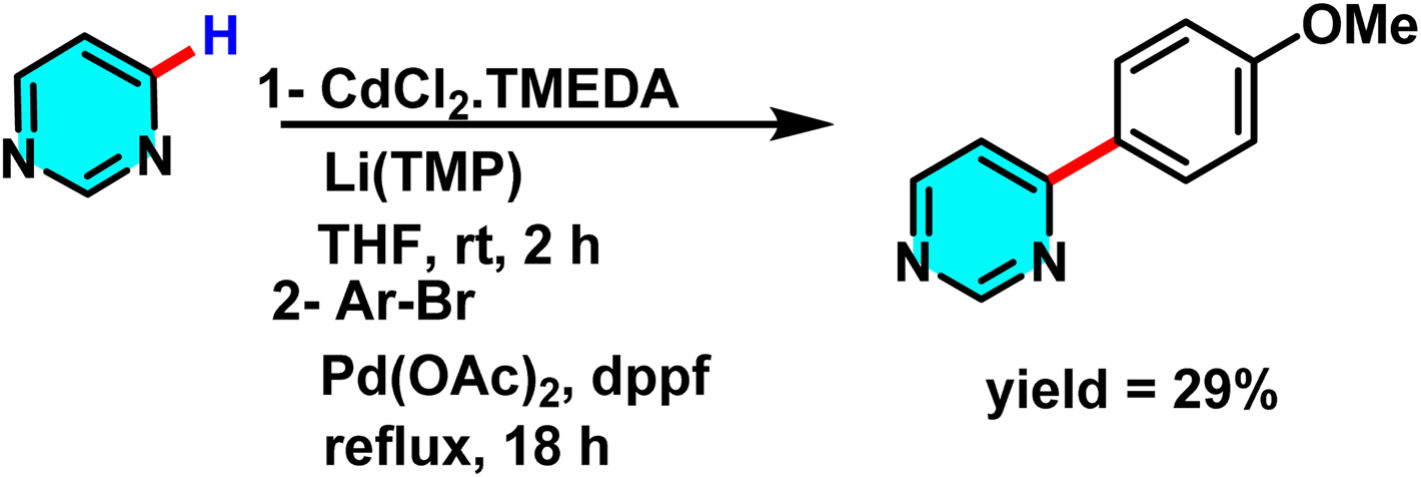
Metalation of pyrimidines with [(tmp)_3_CdLi] and subsequent cross-coupling.

The products resulting from this reaction hold significant potential for utilization in coupling reactions (Scheme 64).^102^

**Scheme 65:**
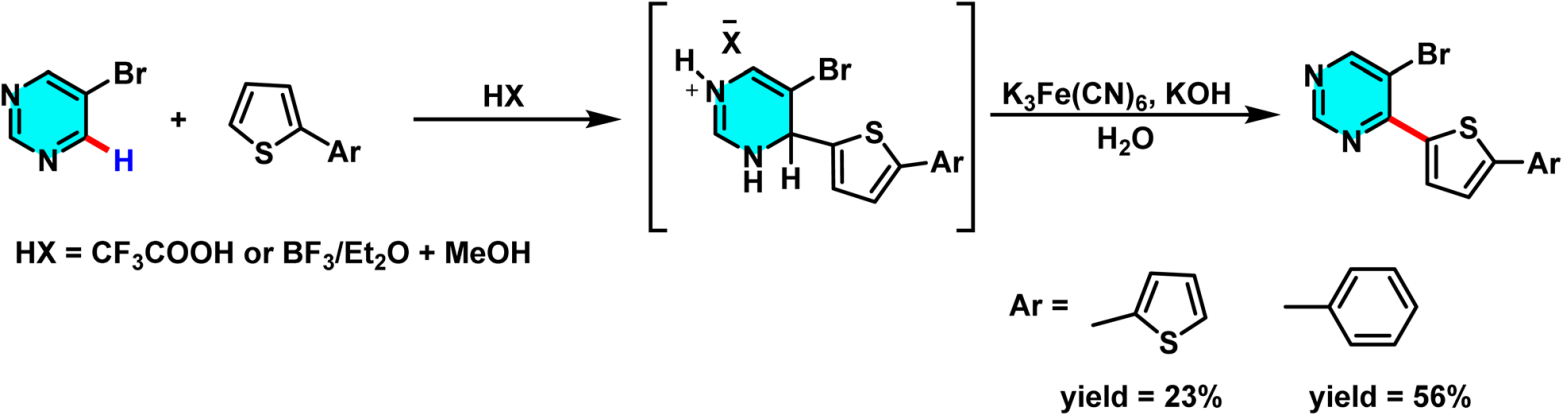
Transition-metal-free C(4)-functionalization of pyrimidine *via* nucleophilic aromatic substitution (S_N_Ar) of hydrogen, promoted by Lewis acids and followed by oxidative rearomatization using K_3_Fe(CN)_6_–KOH.

Verbitskiy and co-workers in 2013 reported a metal-free nucleophilic aromatic substitution of hydrogen (S_N_Ar) strategy for direct C–C bond formation at the C-4 position of halogenated pyrimidines. In this transformation, heteroaryl nucleophiles such as thiophenes are added to the pyrimidine core in the presence of Lewis acids (*e.g.*, CF_3_COOH or BF_3_·Et_2_O/MeOH), which promote ring activation and facilitate formation of a σ-complex intermediate. The reaction proceeds *via* oxidative rearomatization, using K_3_Fe(CN)_6_/KOH as the oxidant, to deliver arylated products in moderate to good yields (Scheme 65).^103^

**Scheme 66:**
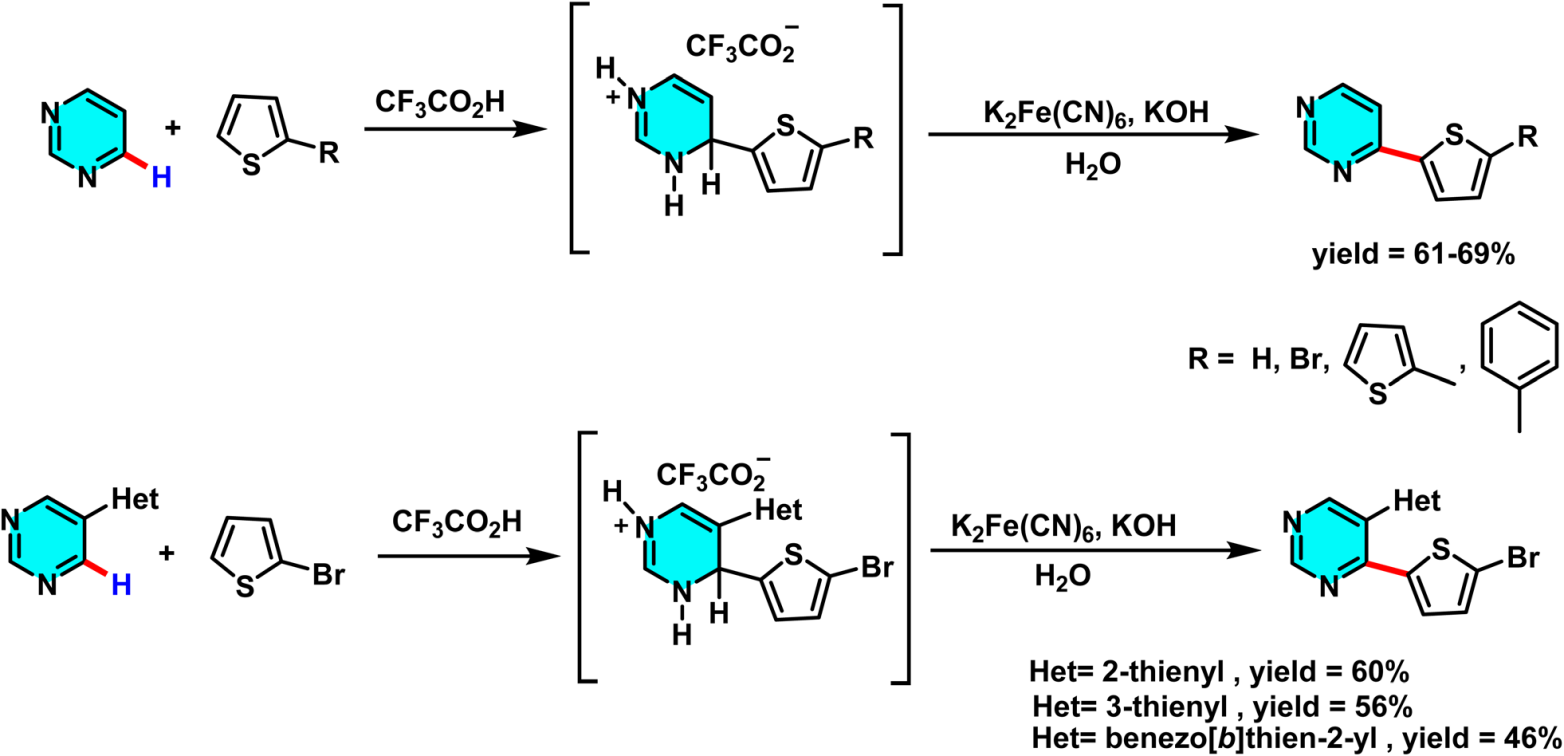
Synthesis of 5-bromo-4-(hetero)arylpyrimidine derivatives *via* nucleophilic substitution of hydrogen (S_N_H) reactions.

The same research group developed a versatile strategy to prepare C(4)-thienyl-substituted pyrimidines with promising antimycobacterial activity. The approach relies on nucleophilic aromatic substitution of hydrogen (S_N_H) to enable direct C–H activation at the C-4 position of the pyrimidine ring. In one pathway, pyrimidine was treated with thiophene derivatives such as 2,2′-bithiophene or 5-phenylthiophene in the presence of acidic media (CF_3_COOH or BF_3_·Et_2_O/MeOH), followed by oxidative rearomatization using K_3_[Fe(CN)_6_] to afford 4-(hetero)arylpyrimidines. A parallel route involved 5-bromopyrimidine, which underwent analogous S_N_H reactions to produce 5-bromo-4-(hetero)arylpyrimidines (Scheme 66).^104^

**Scheme 67:**
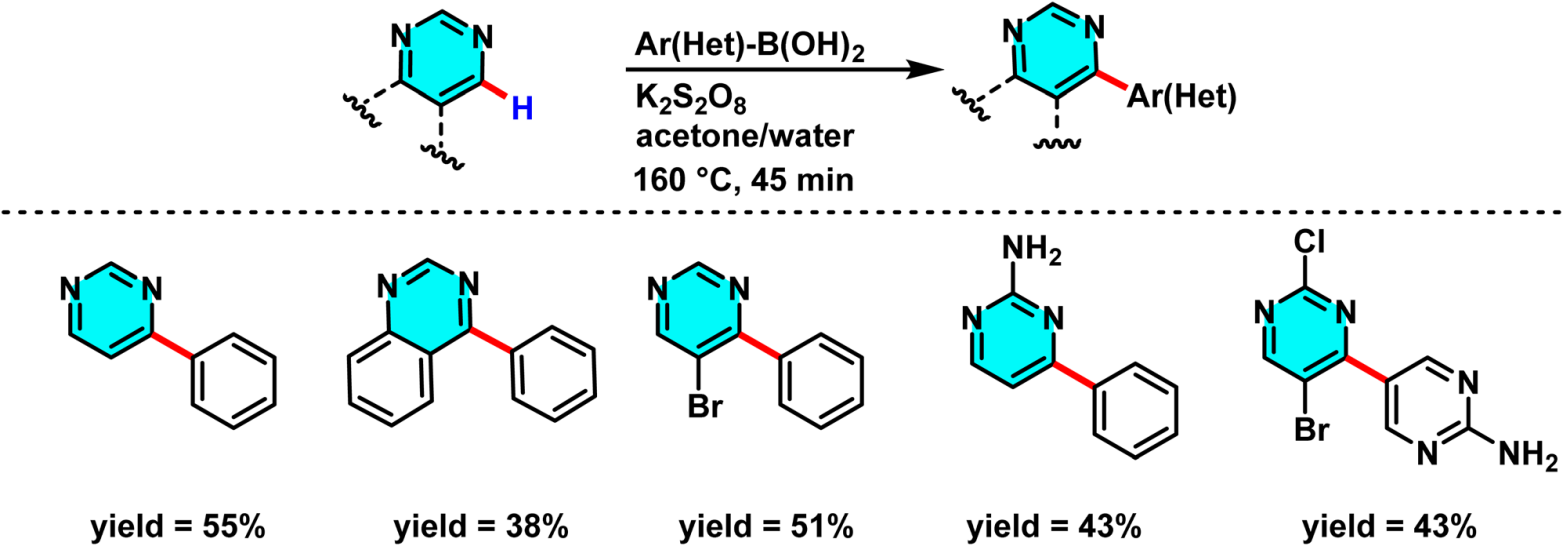
Selective C–H activation at the C-4 position of halogenated pyrimidines *via* radical addition of (hetero)arylboronic acids under metal-free oxidative conditions.

In the ongoing pursuit of metal-free C–H activation methodologies, Singh *et al.* in 2016 developed an innovative metal-free radical approach for coupling halogenated pyrimidines with boronic acids or heteroaryls using potassium persulfate. This reaction proceeds *via* the generation of free radicals and their subsequent addition to the C-4 position of pyrimidine. The findings of this study demonstrate that the methodology is not only applicable to pyrimidines but also extends to other electron-deficient heteroarenes. This metal-free approach represents a key advance in green chemistry, enabling highly selective C–H activation in pyrimidines. The optimized biphasic water/acetone system provided the best efficiency. Its utility was demonstrated in the synthesis of bioactive meriolin-1 *via* coupling of 2-aminopyrimidine with 7-azaindole boronic ester (Scheme 67).

**Scheme 68:**
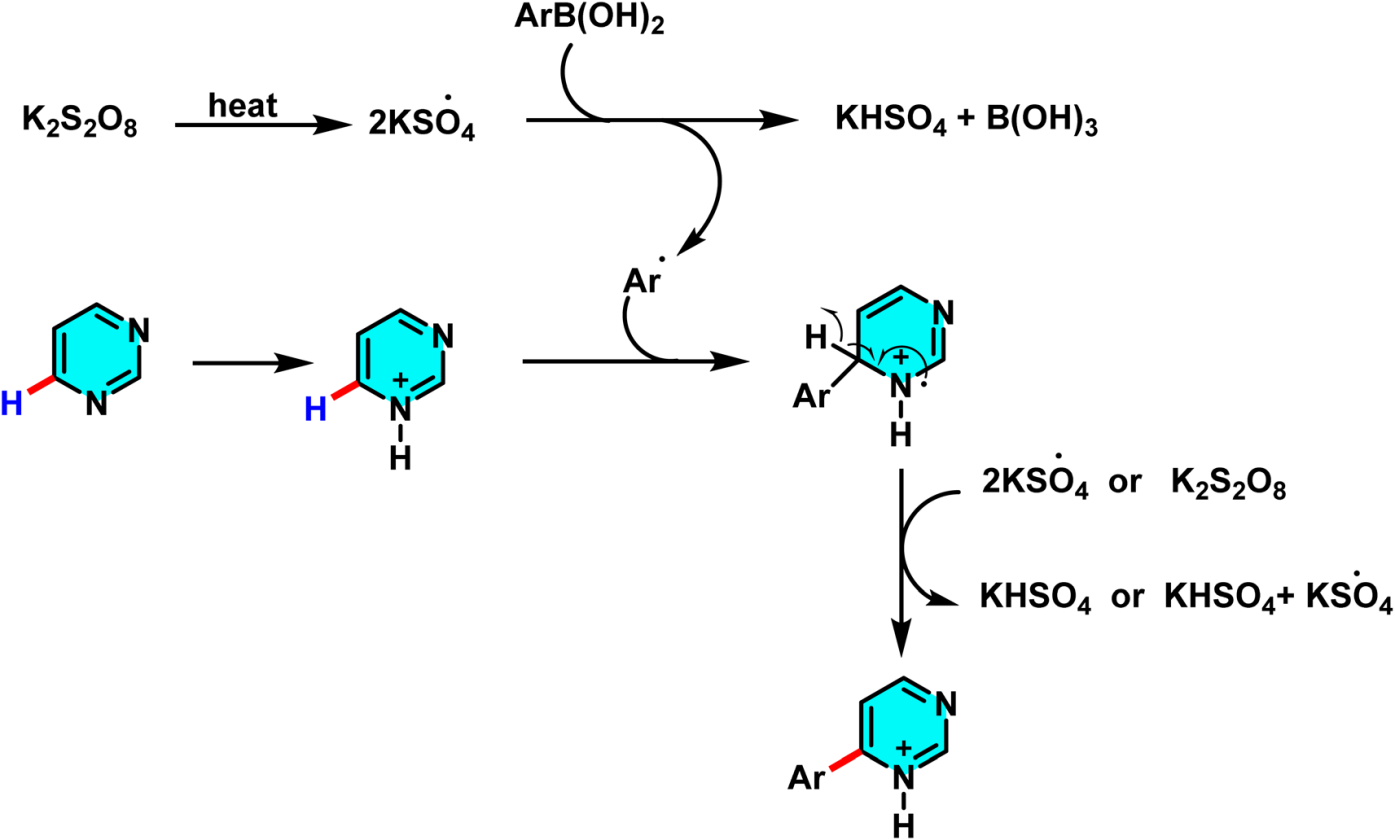
Proposed radical mechanism for the C(4)-selective coupling of halogenated pyrimidines with (hetero)arylboronic acids under metal-free oxidative conditions using potassium persulfate.

The proposed mechanism follows a radical pathway: potassium persulfate initially decomposes into sulfate radicals, which then generate the heteroaryl radical. This radical subsequently couples with pyrimidine, yielding the desired product (Scheme 68). The radical nature of the reaction was substantiated through radical scavenger experiments.^105^

**Scheme 69:**
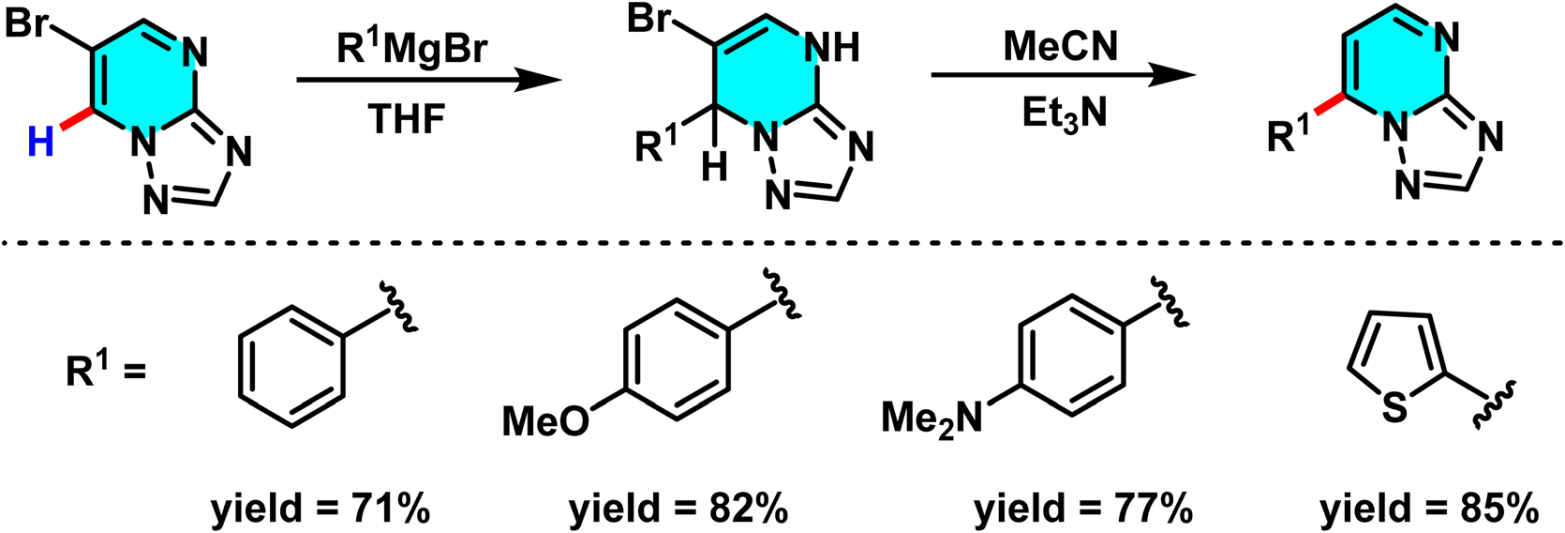
Regioselective C-7 addition of Grignard reagents to [1,2,4]triazolo[1,5-*a*]pyrimidine scaffolds.

A new approach for direct arylation of [1,2,4]triazolo[1,5-*a*]pyrimidine *via* C–H activation was introduced. This method employs Grignard reagents as nucleophilic agents, enabling selective substitution at the C-7 and C-5 positions of [1,2,4]triazolo[1,5-*a*]pyrimidine. In the first step, 6-bromo-[1,2,4]triazolo[1,5-*a*]pyrimidine reacts with a Grignard reagent, leading to the formation of 7-aryl derivatives, which are subsequently stabilized into aromatic products using triethylamine. The second Grignard reaction allows selective substitution at the C-5 position to form σ^H^-adduct intermediates that undergo oxidation *via* molecular oxygen to yield the corresponding final 5,7-diaryl [1,2,4]triazolo[1,5-*a*]pyrimidine products (Scheme 69).

**Scheme 70:**
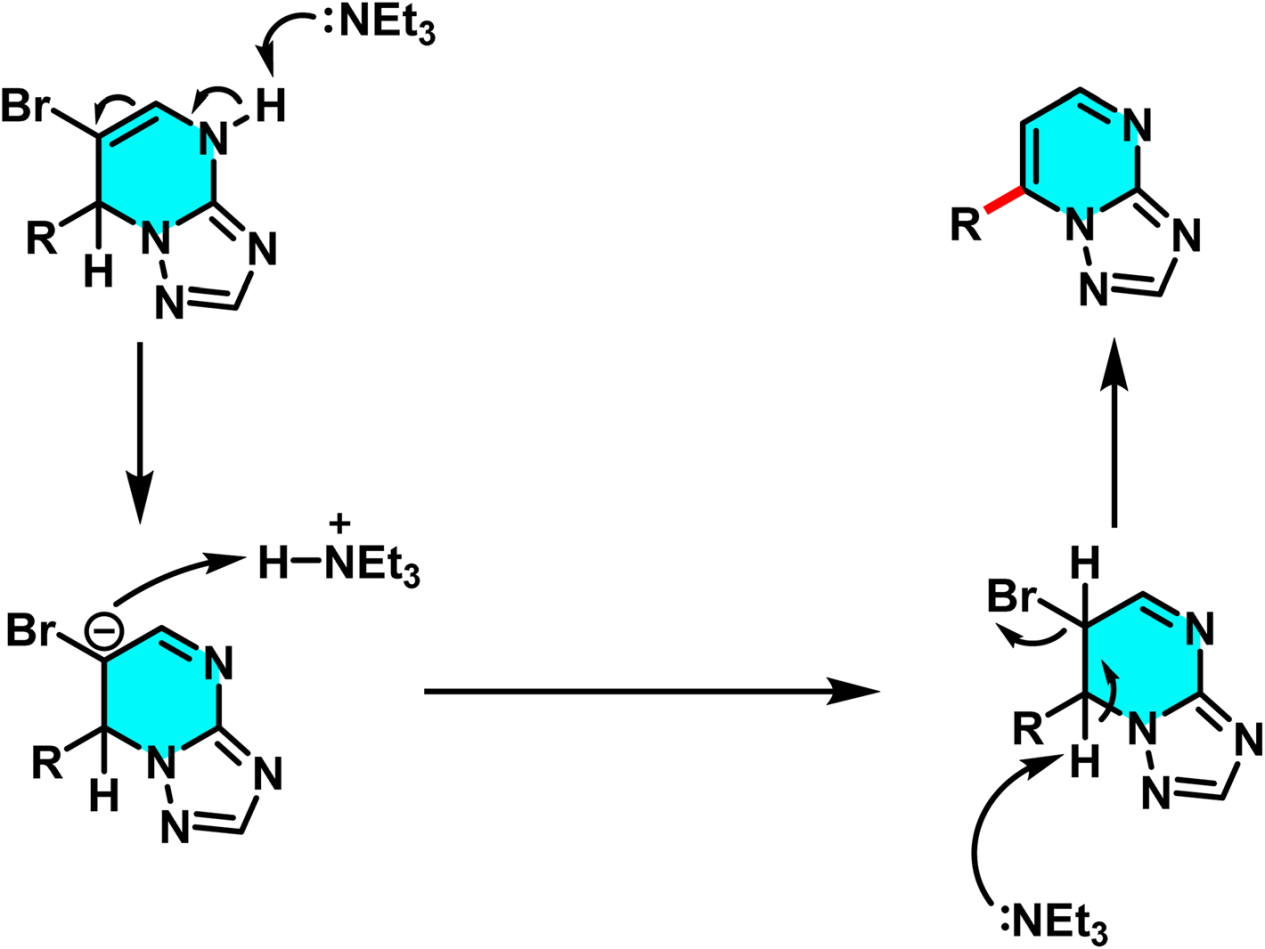
Proposed mechanism for the sequential addition of Grignard reagents to 6-bromo-[1,2,4]triazolo[1,5-*a*]pyrimidine, involving initial C-7 functionalization followed by C-5 addition and oxidative aromatization under aerobic conditions.

The reaction mechanism comprises several key steps. Initially, the Grignard reagent adds to 6-bromo-[1,2,4]triazolo[1,5-*a*]pyrimidine at low temperature (−78 °C), generating an intermediate at the C-7 position. This intermediate then undergoes an elimination process, affording the final aromatic compound at C-7. In the subsequent stage, a second Grignard reaction at the C-5 position forms a 4,5-dihydro intermediate, which is converted into a stable aromatic product upon oxidation in the presence of oxygen (Scheme 70).^106^

**Scheme 71:**
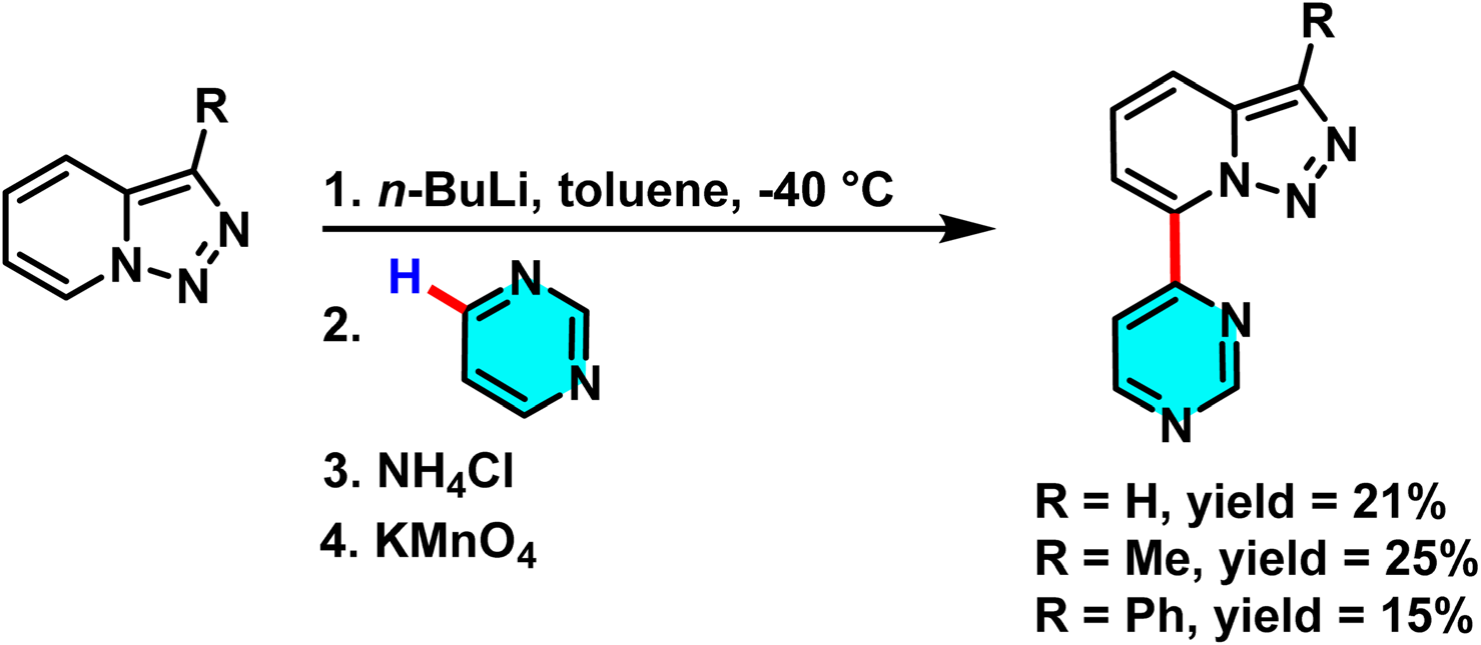
Regioselective formation of 7-(pyrimidinyl)-triazolopyrimidines through nucleophilic attack of 7-lithiotriazolopyrimidines on electrophilic C–H sites of pyrimidine under metal-free conditions.

In 2017, Adam and colleagues reported a metal-free C–H activation method to synthesize triazolopyrimidine derivatives, using pyrimidine as the electrophilic core. 7-Lithiotriazolopyrimidine derivatives reacted with pyrimidines, enabling nucleophilic attack on nitrogen-adjacent carbons. This formed new bonds, yielding 7-(pyrimidinyl)-triazolopyrimidine products (Scheme 71). The reaction selectivity is governed by the electron-withdrawing nature of pyrimidine and the regioselectivity of nucleophilic attack.^107^

**Scheme 72:**
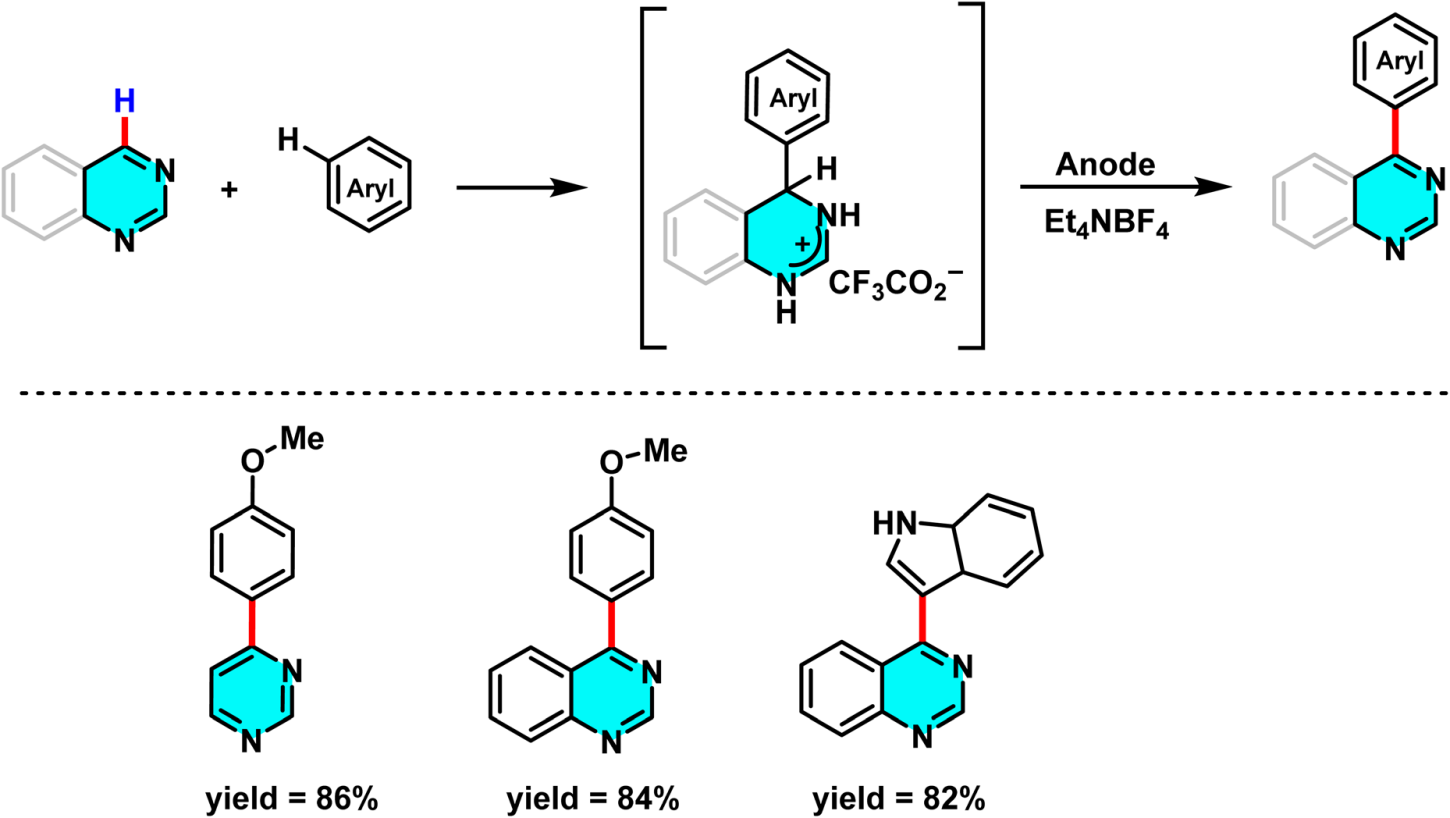
Electrochemical S_N_H reactions of pyrimidines with electron-rich arenes performed under potentiostatic electrolysis.

The current research introduces a novel electrochemical method for nucleophilic hydrogen substitution in pyrimidines and other heteroarenes, setting it apart from previous studies. This approach enables C–C bond formation without requiring leaving groups such as halogens or metal catalysts. The key components of this system include an electrophilic aza-aromatic ring, an aromatic nucleophile, and an electrochemical setup with a supporting electrolyte (Et_4_NBF_4_), operating in a solvent mixture of CH_3_CN and MeOH. The method allows for the selective substitution of aryl or heteroaryl groups at positions such as C-4 or C-5 of pyrimidine (Scheme 72).

The mechanism initiates with nucleophilic attack at the electrophilic C–H site of pyrimidine, forming a σ-complex. Voltammetric studies confirm the equilibrium of this intermediate. Subsequent electrolysis oxidizes the complex, resulting in the loss of two electrons and one proton to restore aromaticity and yield the S_N_H product.^108^

**Scheme 73:**
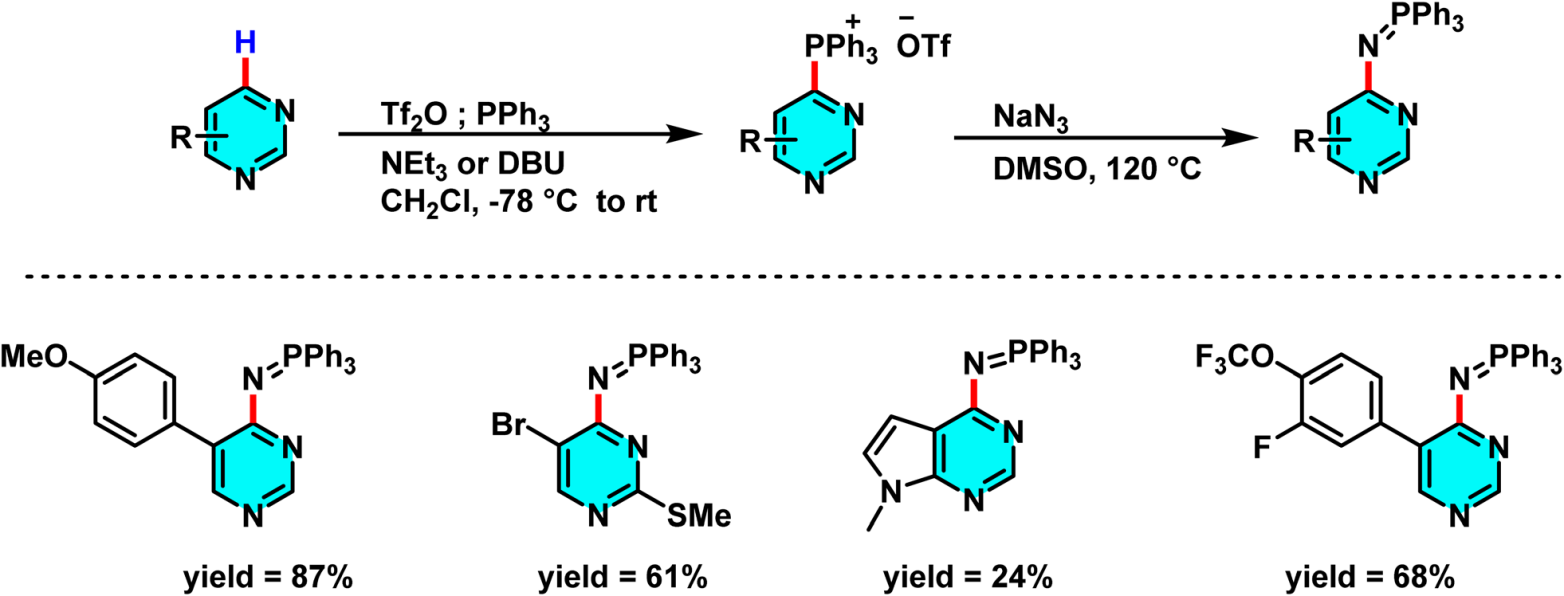
Metal-free C–H amination of pyrimidines *via* phosphonium intermediates and NaN_3_ substitution.

In the sustained pursuit of metal-free methodologies for heterocycle activation, a novel approach for the amination of nitrogen-containing heterocycles, particularly pyridines and diazines, has been developed using phosphonium salts. This method involves the initial formation of a phosphonium salt from the target heterocycle, followed by its reaction with sodium azide (NaN_3_) to generate iminophosphoranes. Unlike conventional methodologies relying on transition metals or aryl halides, this process enables direct amination *via* nucleophilic substitution of phosphonium salts. Experimental results demonstrated that employing DMSO as the solvent and conducting the reaction at 120 °C provided optimal reaction conditions (Scheme 73).

**Scheme 74:**
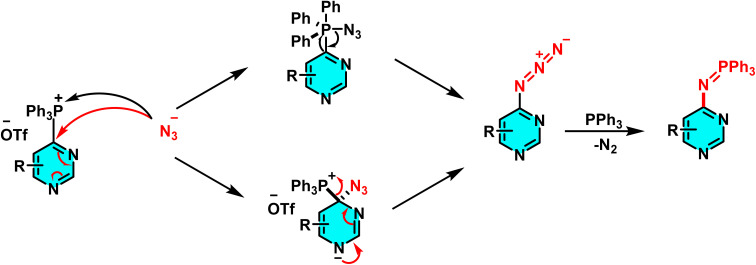
Mechanistic pathways of C–H amination of pyrimidines *via* phosphonium intermediates and NaN_3_ substitution.

The reaction mechanism featured nucleophilic substitution of sodium azide at the phosphonium salt, forming an iminophosphorane intermediate *via* the Staudinger reaction. This intermediate underwent further transformations to produce aniline derivatives, isothiocyanates, and other nitrogen-containing compounds. Evaluation showed high selectivity for substituted pyridines and diazines (Scheme 74).^109^

**Scheme 75:**
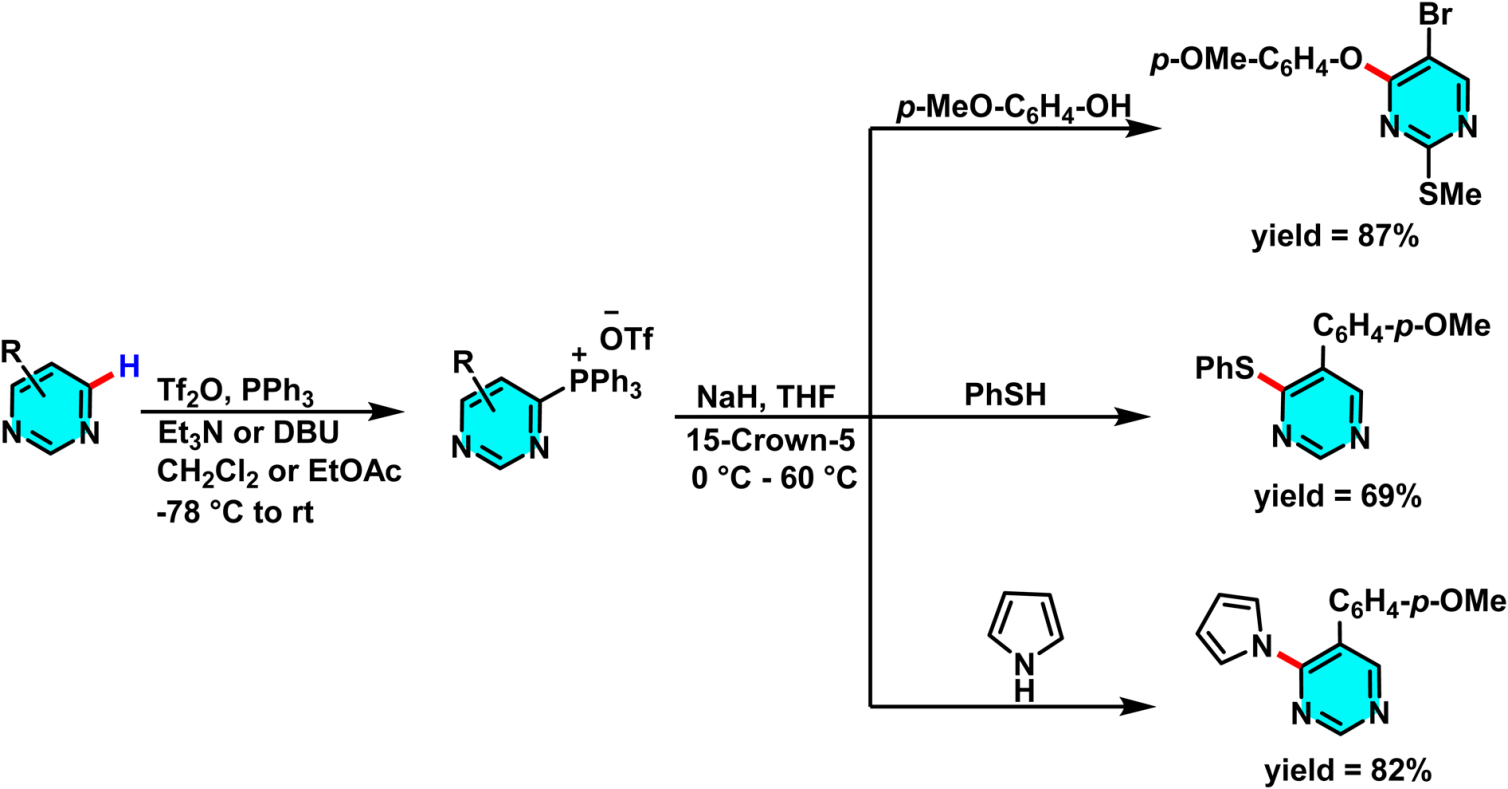
Metal-free activation of pyrimidine C–H bonds through *in situ* formation of phosphonium salts and subsequent azide-mediated transformation to iminophosphoranes.

Patel *et al.* in 2018 presented, a novel method for C–H activation in pyrimidine *via* heterocyclic phosphonium intermediates. Unlike conventional strategies that typically require transition metals and halogenated pyrimidine derivatives, this approach enables direct C–H activation at specific positions of the pyrimidine ring. The process involves the initial reaction of pyrimidine with a phosphonium reagent, leading to the formation of an activated phosphonium salt. This salt subsequently undergoes nucleophilic substitution with sodium azide (NaN_3_), yielding a stable iminophosphorane intermediate (Scheme 75).

The reaction mechanism consists of three key stages. First, the C–H bond in pyrimidine is activated through its interaction with a phosphonium reagent, resulting in the formation of a heterocyclic phosphonium salt. This salt, acting as a reactive intermediate, then undergoes nucleophilic substitution with sodium azide (NaN_3_) to generate a stable iminophosphorane. Under appropriate conditions, this intermediate can be further transformed into a variety of pyrimidine derivatives, facilitating the formation of C–N or C–C bonds.^110^

**Scheme 76:**
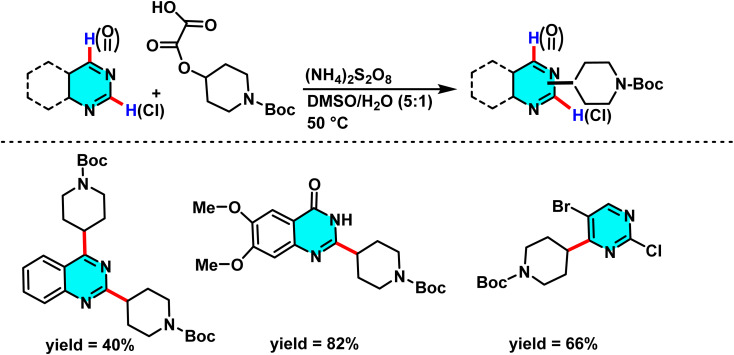
Alkylation of pyrimidine C–H bonds through a radical Minisci pathway.

A new approach for direct alkylation of pyrimidine C–H bonds was introduced. This study utilized the Minisci reaction, which involves C–C bond formation through free radical pathways. Ammonium persulfate was employed as a sulfate radical generator, playing a key role in hydrogen abstraction from pyrimidine and the formation of a radical intermediate. Various alkylating agents were explored in this reaction (Scheme 76).

**Scheme 77:**
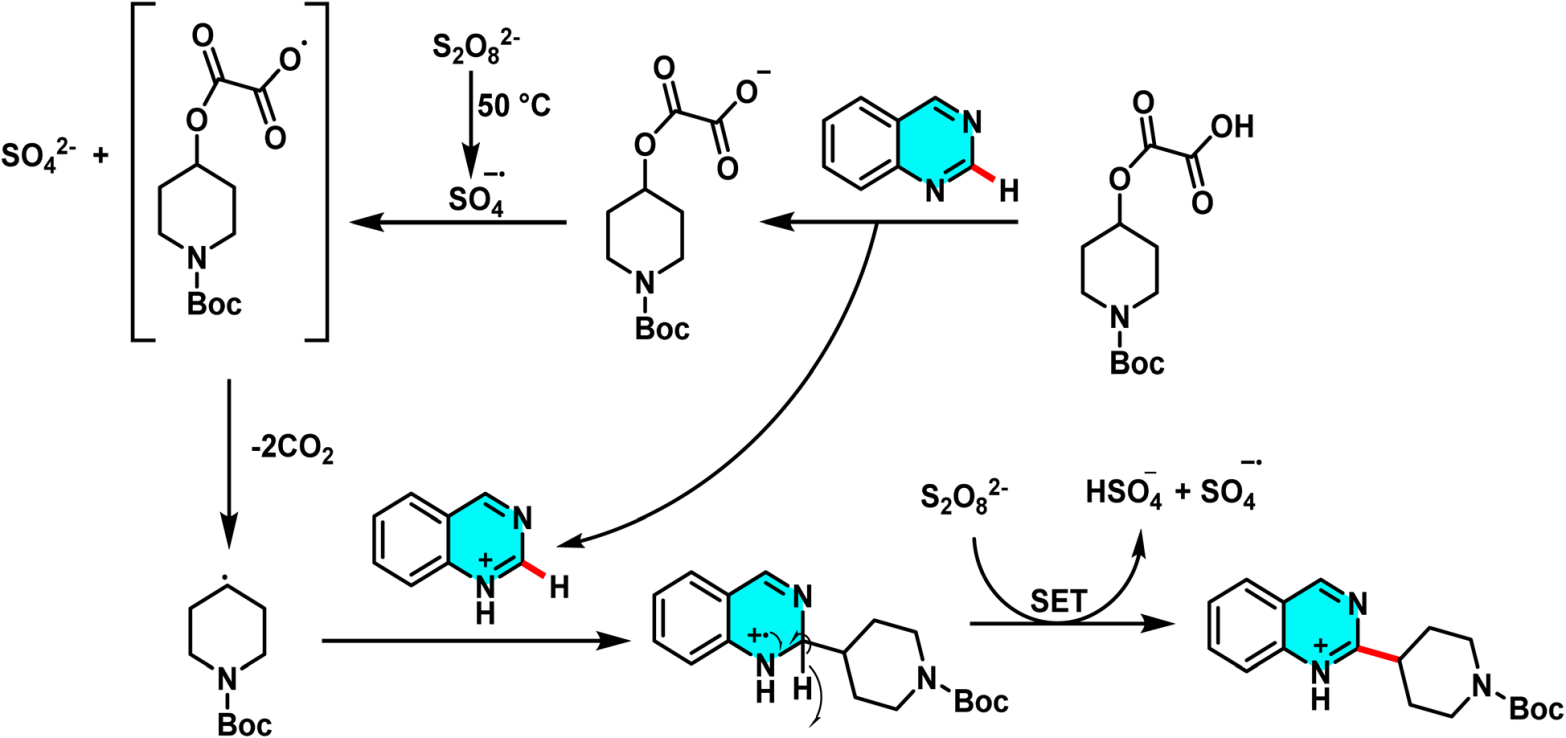
Proposed mechanism for the radical-mediated C–H alkylation of pyrimidine *via* the Minisci reaction.

Proposed mechanistic studies indicate that the entire transformation proceeds through a radical-mediated pathway. This hypothesis was confirmed by the addition of radical scavengers such as TEMPO and BHT, which completely inhibited the reaction. Furthermore, the use of cyclic compounds as alkyl sources resulted in ring-opening products under the reaction conditions, further supporting the radical nature of the process (Scheme 77).^111^

**Scheme 78:**
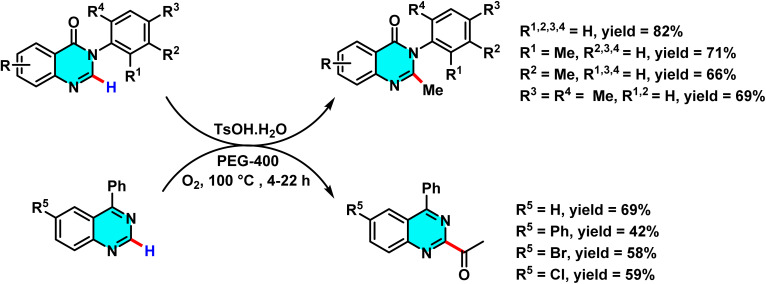
Methylation and acetylation of pyrimidine derivatives using PEG-400 as a sustainable carbon source.

Reactions for acetylation and methylation of N-heteroarenes were conducted using PEG-400 as a new carbon source. This method proved efficient and green for activating C–H bonds in pyrimidines. PEG-400 enabled metal-free C–H acetylation and methylation of N-heteroarenes by acting as both solvent and radical precursor. Under oxidative and acidic conditions, PEG-400 underwent C–O and C–C bond cleavage to generate methyl and acetyl radicals. TsOH·H_2_O protonated pyrimidine derivatives and facilitated PEG-400 degradation, producing radical intermediates (Scheme 78).

**Scheme 79:**
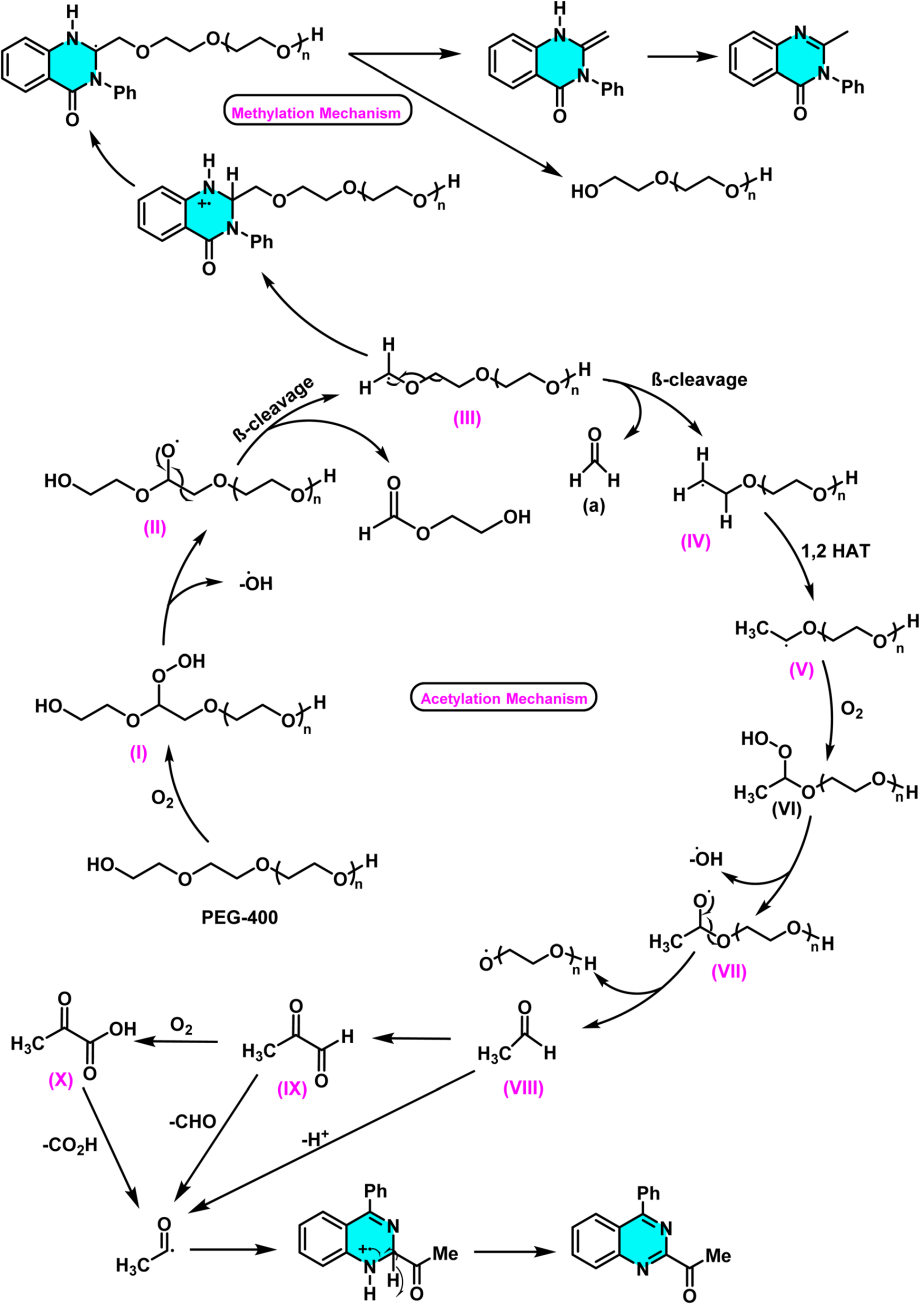
Environmentally benign mechanism for C–H functionalization of pyrimidines using PEG-400 as a sustainable carbon source.

The proposed mechanism for the methylation and acetylation of heteroarenes is illustrated in Scheme 79. Initially, PEG-400 reacts with oxygen to form α-hydroperoxide (I). The thermal decomposition of this compound then generates an alkoxy radical with an oxygen centre (II), which undergoes two sequential steps, β-cleavage and 1,2-HAT, to yield intermediate (V). This intermediate further reacts with O_2_, undergoing a series of consecutive decompositions to produce acetaldehyde. Under oxidative conditions, the acetyl radical, generated *in situ* from intermediates (IX) or (X) (which themselves derive from compound (VIII)), reacts with the protonated electrophilic heteroarene, such as quinoline, following a pathway similar to the Minisci reaction. This leads to the formation of an aminium radical cation. Given the significant acidity of the α-C–H bond, this intermediate undergoes deprotonation to form an α-amino radical, which finally undergoes oxidation to yield acetylated quinoline.^112^

A metal-free approach for heteroarylation of pyrimidine and its derivatives *via* the Minisci reaction was developed. This method utilizes simple alkanes such as cyclohexane as a carbon source, along with TsNHMe and CF_3_CH_2_OH as radical initiators. In this reaction, pyrimidine and its derivatives act as radical acceptors, while alkanes serve as alkyl radical donors.

**Scheme 80:**
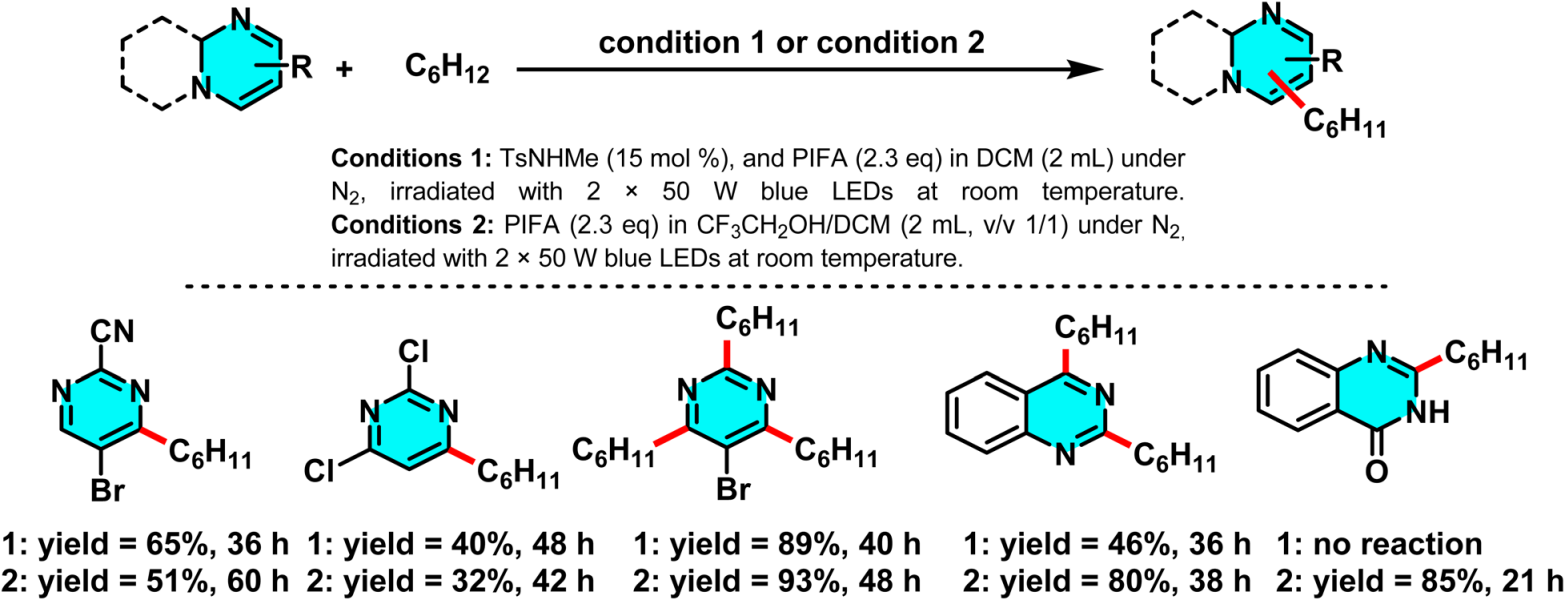
Metal-free Minisci-type heteroarylation of pyrimidines under visible light using alkanes as radical donors.

The radical generation process is facilitated by (phenyliodine(iii) bis(trifluoroacetate)) (PIFA) under visible light irradiation. TsNHMe and CF_3_CH_2_OH, in the presence of PIFA, generate radicals that initiate the hydrogen atom transfer (HAT) process. A key advantage of this reaction is that it does not require additional acid, as TFA, which is produced *in situ* from PIFA, activates pyrimidine by protonation. This feature distinguishes the method from conventional Minisci reactions because of the significantly improving it (Scheme 80).

**Scheme 81:**
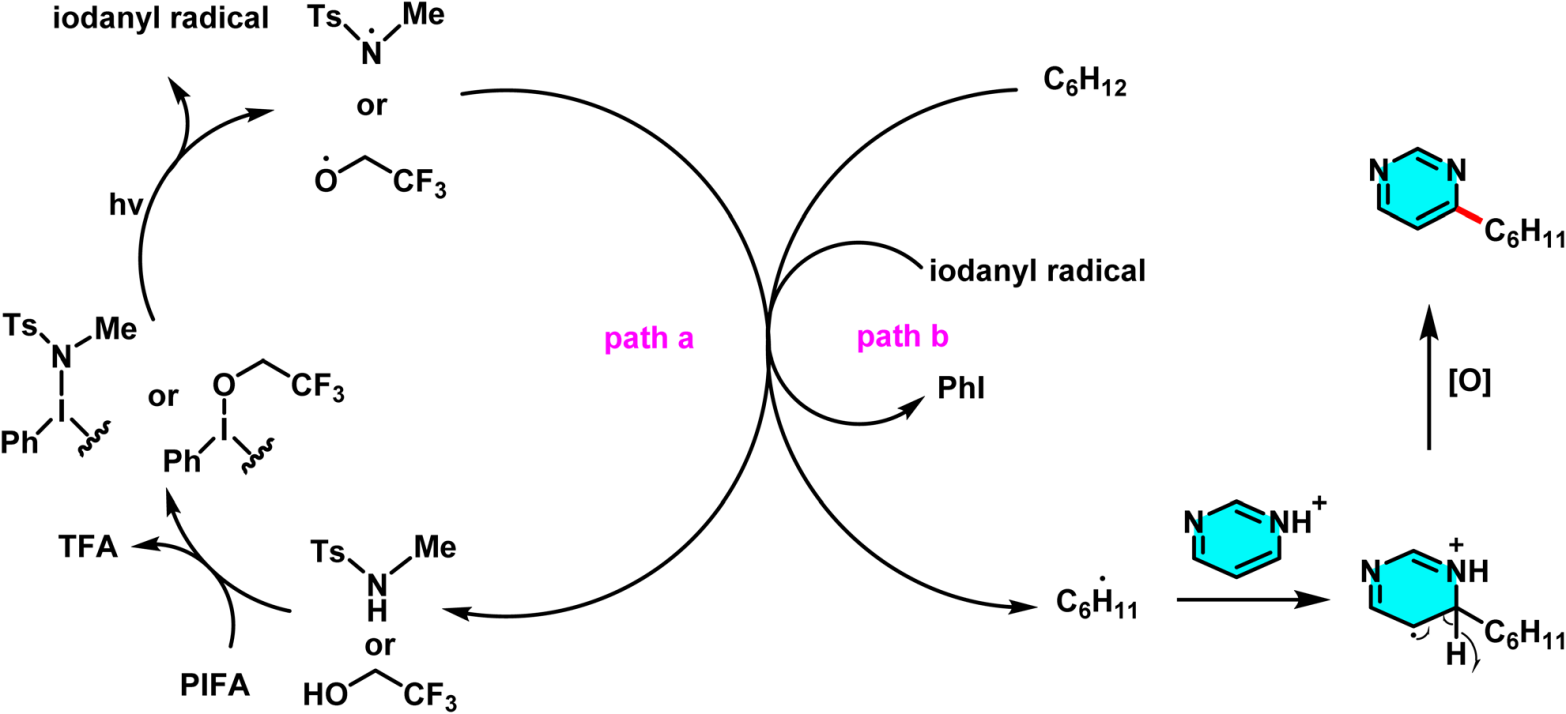
Mechanistic pathway for the visible-light-induced, metal-free Minisci-type alkylation of pyrimidines using alkanes as radical donors.

The proposed reaction mechanism involves the initial interaction of PIFA with the amide or alcohol, producing active radicals (such as amide or alkoxy radicals) under visible light irradiation (Scheme 81). These radicals abstract a hydrogen atom from the alkane, generating an alkyl radical that subsequently attacks the activated pyrimidine. Finally, an oxidation step leads to the formation of the heteroarylated product.^113^

**Scheme 82:**
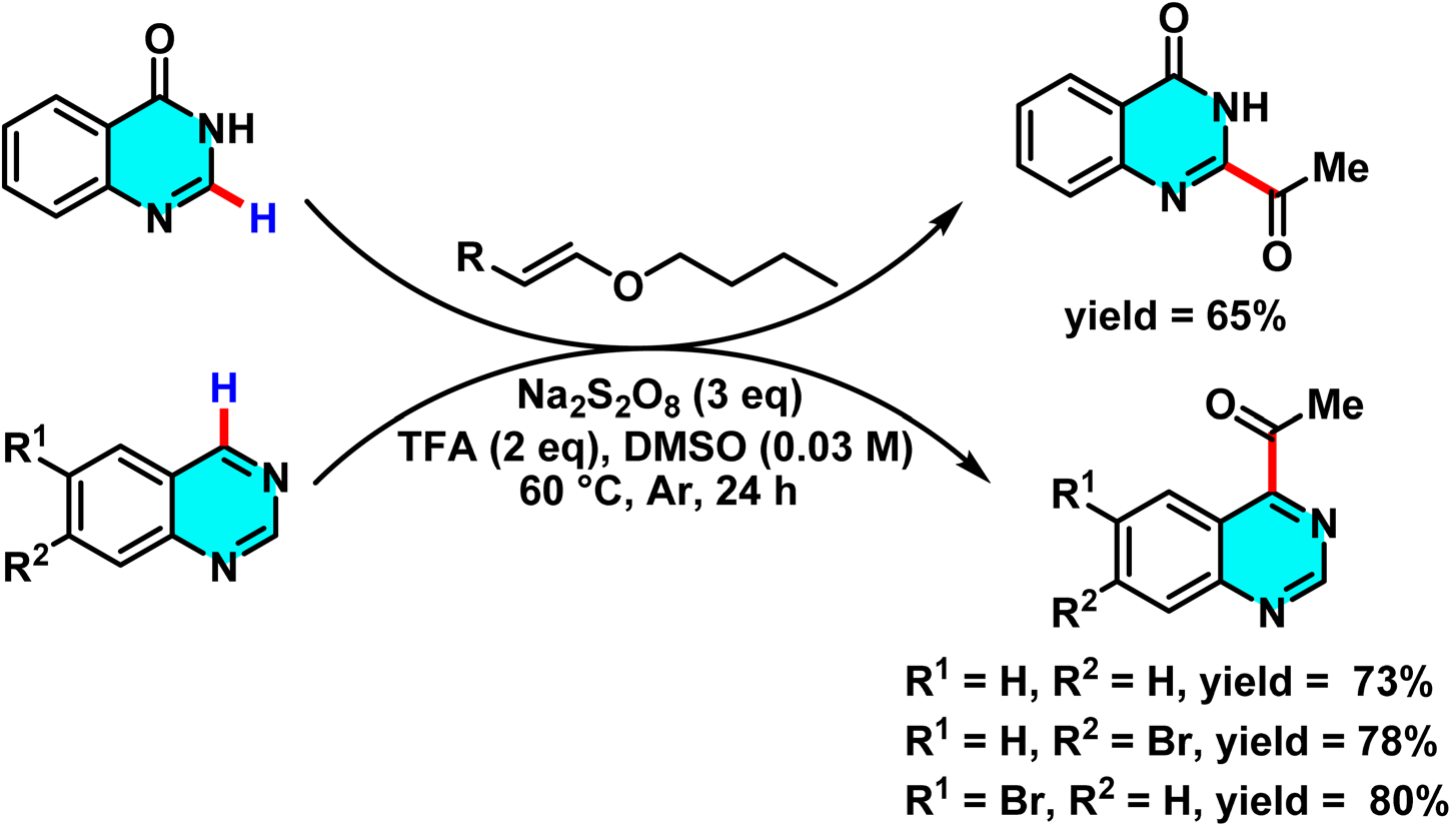
Acylation of pyrimidine derivatives under metal-free conditions.

With continued advancements in C–H bond activation devoid of metal catalysts, in 2021, a study explored a reaction system comprising vinyl ether, sodium persulfate, and DMSO. This investigation specifically targeted the direct acetylation of C–H bonds in N-heteroarenes, including pyrimidines. In this approach, vinyl ether was employed as an economical and readily available precursor for generating acetyl radicals, operating efficiently without the necessity of metal catalysts, light irradiation, or elevated temperatures. Sodium persulfate as an oxidant undergoes homolytic cleavage in DMSO, generating sulfate radicals that subsequently facilitate the formation of acetyl radicals. These radicals selectively attack the C–H bonds at the C-2 and C-4 positions of pyrimidines, followed by rearomatization, leading to the formation of acetylated products with high yields (Scheme 82).

**Scheme 83:**
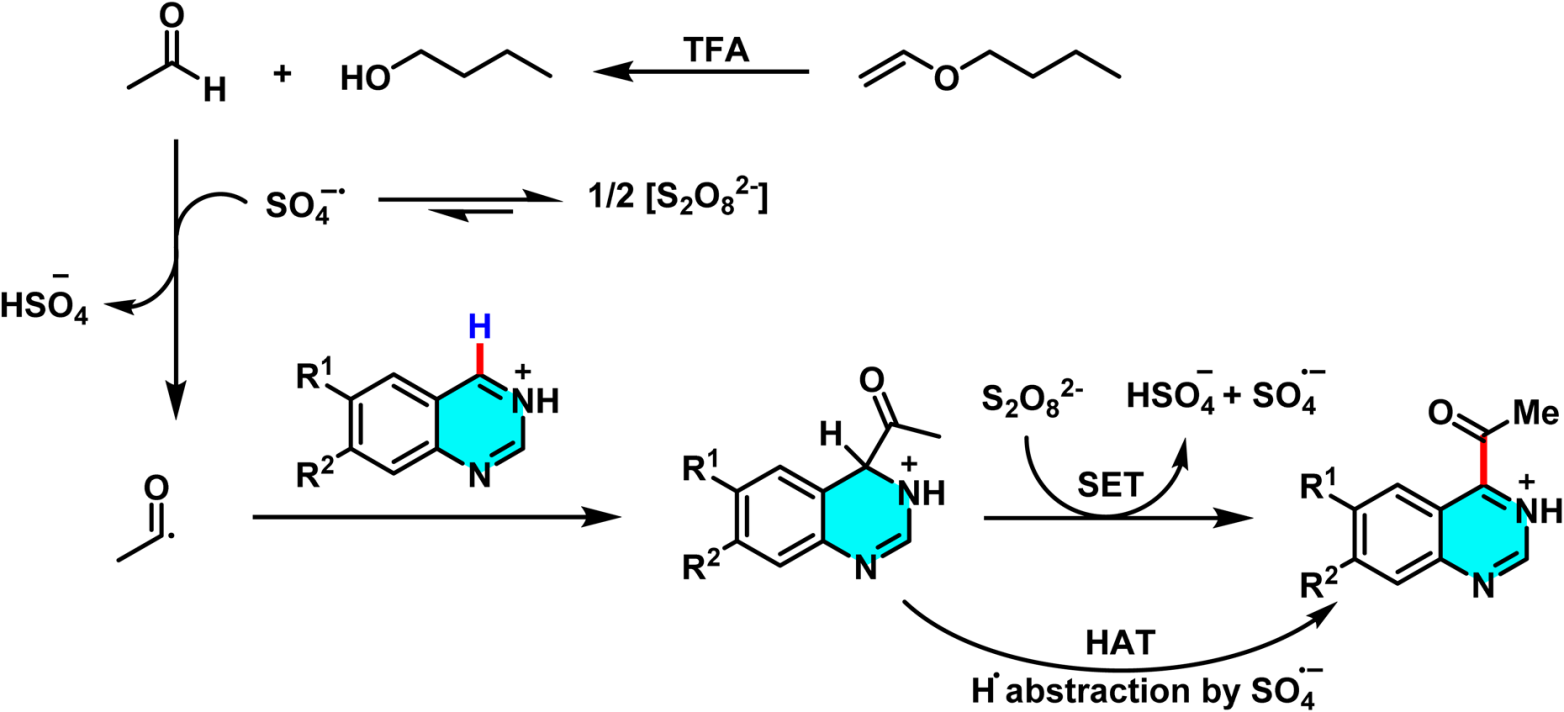
Plausible reaction mechanism for the acetylation of pyrimidines under metal-free conditions.

Mechanistically, DMSO accelerated persulfate decomposition and promoted radical generation. TFA aided vinyl ether breakdown into acetaldehyde and protonated pyrimidine, boosting its reactivity to acetyl radical attack (Scheme 83). The method showed compatibility with pyrimidines, quinolines, and quinazolines, maintaining efficiency on a gram scale.^114^

### 3.1 Critical analysis for metal-free C–H activation

Metal-free C–H activation methods in pyrimidines, primarily based on Minisci-type radical reactions using persulfates or phosphonium intermediates, offer sustainable and mild synthetic routes well-suited for pharmaceutical applications. By avoiding metal catalysts, these approaches reduce metal-associated toxicity, contamination, and purification challenges, improving compatibility with sensitive functional groups.

However, these methods face significant limitations. Regioselectivity is generally lower compared to metal-catalyzed protocols, often resulting in regioisomeric mixtures and decreased yields. The use of stoichiometric radical initiators compromises atom economy and generates chemical waste. Additionally, the presence of multiple reactive sites in complex pyrimidines increases side reactions, limiting substrate scope. The lack of efficient catalytic radical initiators further impacts process efficiency and scalability.

Addressing these challenges through the development of catalytic radical systems and integrating photochemical activation represents a promising direction. Such advances could enhance selectivity, reduce reagent load, and expand the applicability of metal-free C–H functionalization, establishing these methods as green and practical alternatives for synthesizing pyrimidine-based heterocycles.

## 4 Photochemical strategies for direct C–H activation of pyrimidines and related heteroarenes

Visible-light-mediated photochemical methods have emerged as powerful tools for the direct C–H activation of pyrimidines and related heteroarenes, offering mild conditions, environmental sustainability, and the elimination of harsh oxidants or high temperatures traditionally required in such transformations. These approaches, often leveraging photoredox catalysis or photocatalyst-free systems, have facilitated the selective functionalization of pyrimidine scaffolds, yielding derivatives with significant potential in pharmaceutical and bioactive compound synthesis. This section reviews key advancements in photochemical C–H activation strategies, highlighting their mechanistic insights, synthetic utility, and green chemistry attributes.

**Scheme 84:**
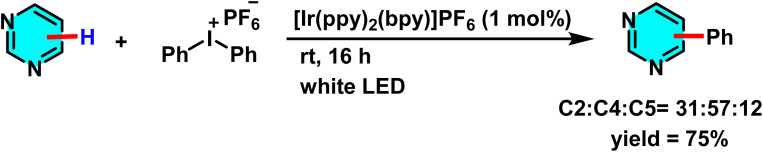
Photocatalyzed arylation of pyrimidine using [Ir(ppy)_2_(bpy)]PF_6_ as photocatalyst.

In 2013, Tobisu and co-workers introduced a protocol employing diaryliodonium salts (Ar_2_I^+^) as a source of aryl radicals under white LED irradiation (400–750 nm) to achieve the arylation of pyrimidine in the presence of the photocatalyst [Ir(ppy)_2_(bpy)]PF_6_ in moderate yields (approximately 40%). Conducted in acetonitrile, this process involves the selective addition of aryl radicals derived from Ar_2_I^+^ to the pyrimidine ring, indicative of a homolytic aromatic substitution rather than an ionic or metal-mediated mechanism (Scheme 84).

**Scheme 85:**
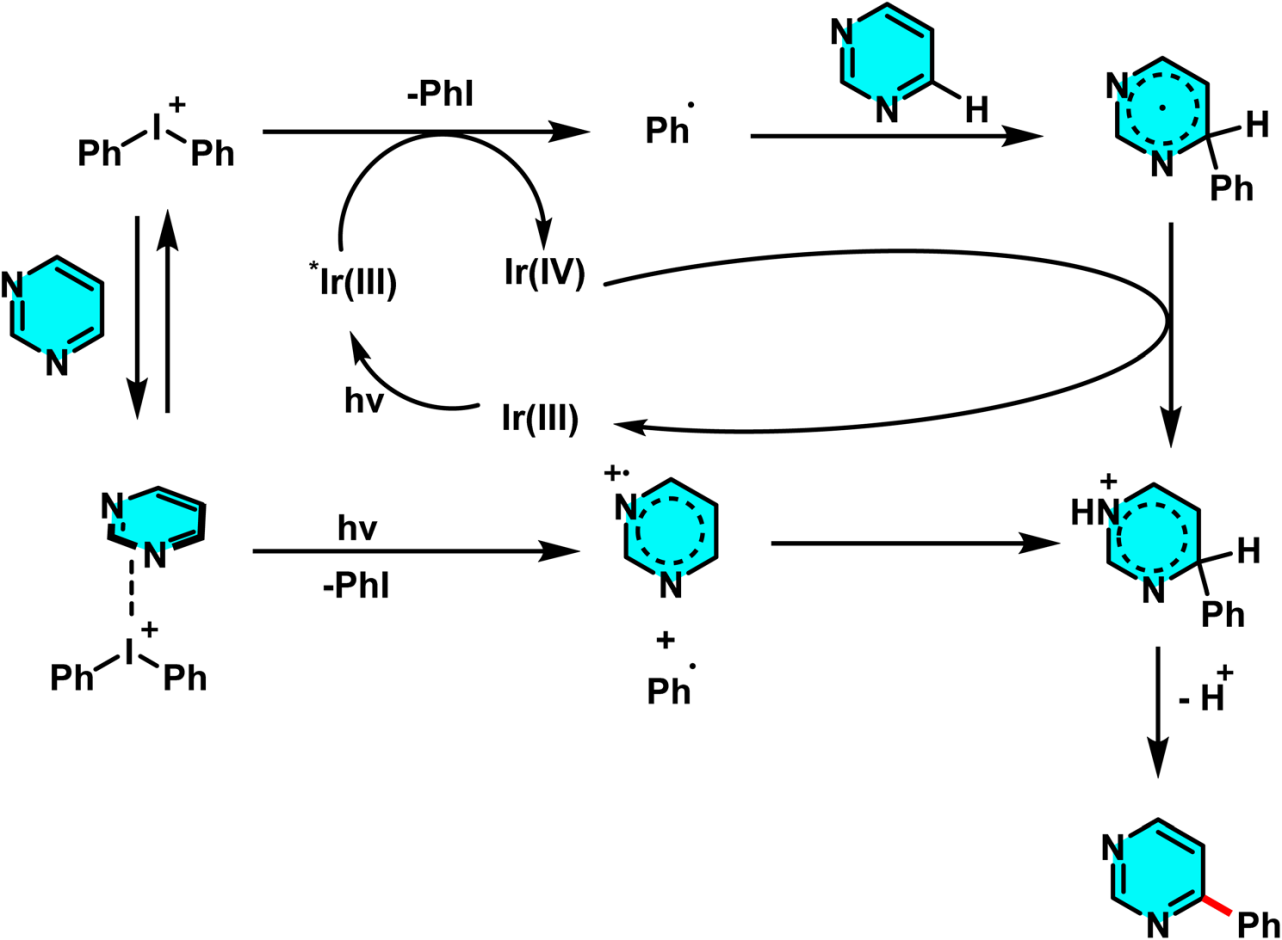
Mechanistic proposal for phenyl group addition to the pyrimidine core.

The mechanism began with a SET from the photoexcited *Ir(iii) state of the photocatalyst to Ar_2_I^+^, breaking it into an aryl radical and Ir(iv). The aryl radical attacked the pyrimidine ring, forming an unstable intermediate that oxidized with Ir(iv) and lost a proton to restore aromaticity. Stern–Volmer experiments confirmed this photoredox cycle, showing Ar_2_I^+^ quenched *Ir(iii) luminescence, highlighting its role in starting the reaction (Scheme 85).^115^

**Scheme 86:**
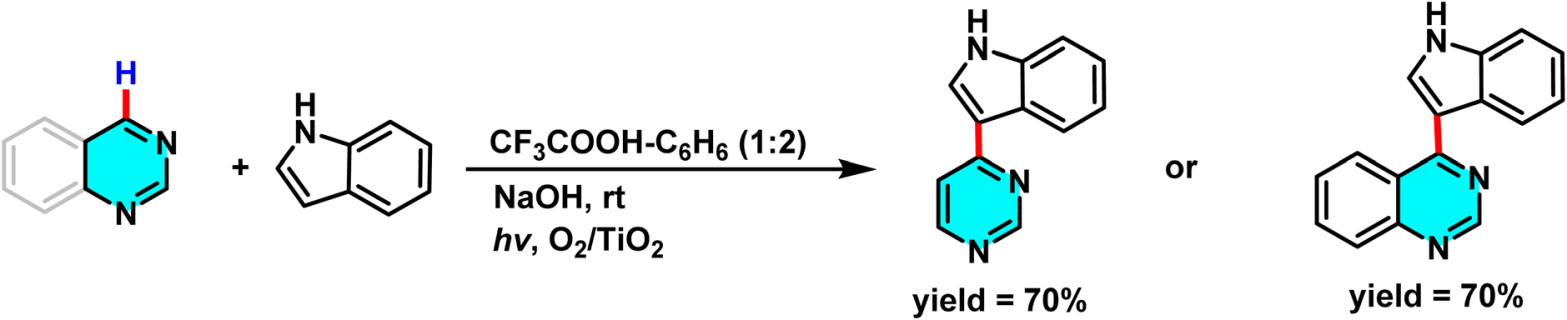
C–H/C–H coupling of pyrimidines with indoles and pyrrole catalyzed by TiO_2_ under O_2_ atmosphere.

In 2015, Utepova *et al.* achieved metal-free C–H/C–H coupling of pyrimidines with indoles and pyrroles using atmospheric O_2_ and nanosized TiO_2_ under Xe-lamp irradiation. Reactions proceeded in TFA/benzene (1 : 2) or HCl/MeOH (1 : 2) over 5–10 h with air bubbling, delivering arylated products in satisfactory yields (Scheme 86). This protocol exhibits high selectivity, avoiding byproducts such as homo coupling, while TiO_2_ remains recyclable for up to five cycles, producing only water as a waste product.

**Scheme 87:**
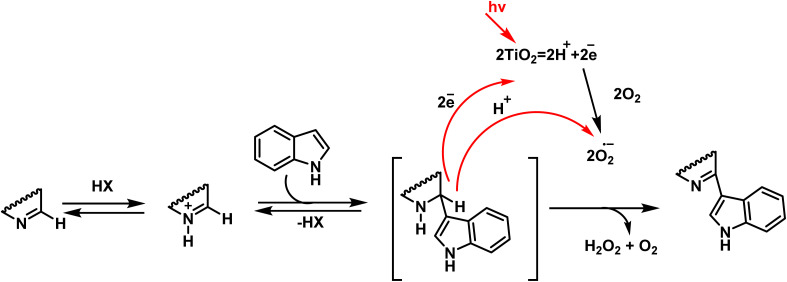
Plausible mechanism for the metal-free S_N_H transformation involving radical intermediates under visible light.

The mechanism involved hydrogen nucleophilic substitution (S_N_H), where pyrimidine protonation in an acidic medium increased its electrophilicity, enabling indole to attack the C-4 position and form a σ^H^-adduct intermediate. Xe lamp irradiation activated TiO_2_, generating electron–hole pairs (e^−^/h^+^); electrons reacted with O_2_ to form superoxide radicals (O^2−^). These radicals removed an electron from the intermediate, and proton loss oxidized it into the aromatic product. Pyrimidine-indole intermediates were quantitatively oxidized in aqueous alcohol with NaOH (Scheme 87).^116^

**Scheme 88:**
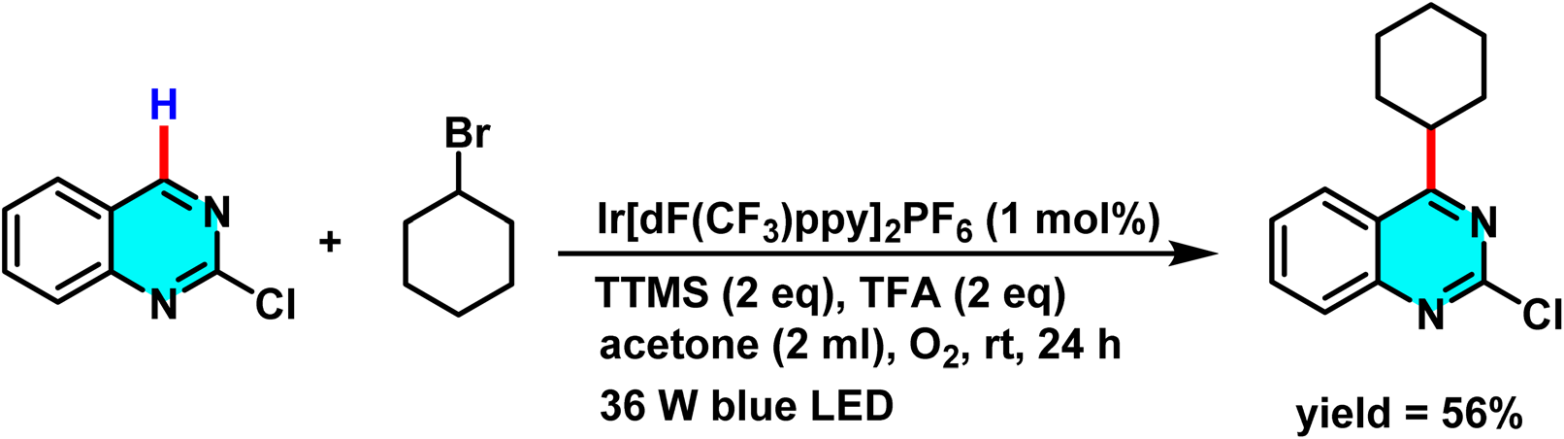
Visible-light-driven C–H alkylation of quinazoline using unactivated alkyl bromides, Ir[dF(CF_3_)ppy]_2_(dtbbpy)PF_6_ as the photocatalyst, and TTMS/O_2_ as the radical system under mild conditions.

Dong and co-workers (2019) introduced a method to alkylate heteroarenes, including pyrimidine derivatives, with inactivated alkyl halides. They used the photocatalyst Ir[dF(CF_3_)ppy]_2_(dtbbpy)PF_6_ and 36 W blue LED irradiation as the energy source, with molecular oxygen (O_2_) and tris(trimethylsilyl)silane (TTMS) present. Conducted in acetone with TFA as a co-reagent, this reaction delivers commendable yields for heteroarenes such as quinazoline (Scheme 88). The adoption of O_2_ as an oxidant, alongside the avoidance of harsh conditions like elevated temperatures or high-energy UV light, renders this approach particularly suitable for pharmaceutical applications and late-stage functionalization.

**Scheme 89:**
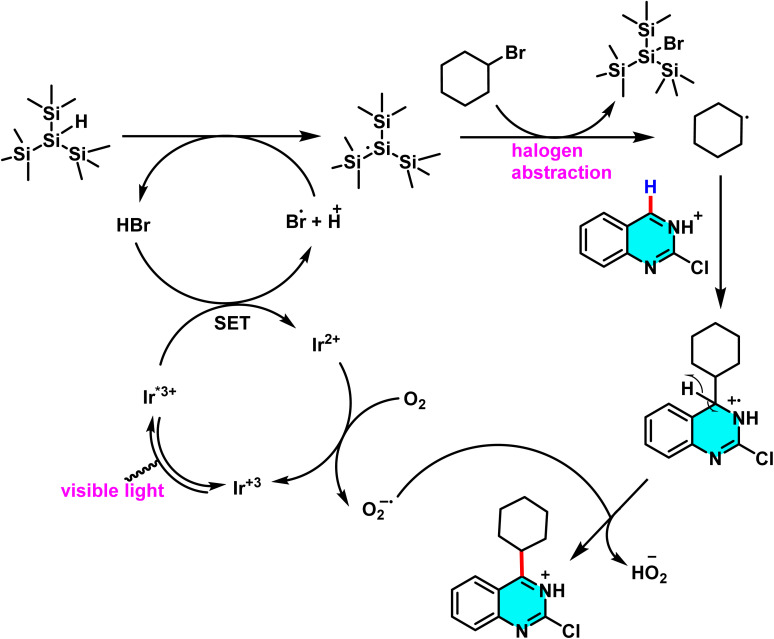
Proposed mechanism for Ir[dF(CF_3_)ppy]_2_(dtbbpy)PF_6_-photocatalyzed C–H alkylation of pyrimidine derivatives using unactivated alkyl halides under visible-light irradiation and aerobic oxidative conditions.

The mechanism began with photoexcitation of the photocatalyst under visible light, creating an excited *Ir(iii) state. This reacted with TTMS to form a silyl radical (TMS_3_Si˙), which removed bromine from the alkyl bromide to produce an alkyl radical (R˙). The radical added to the TFA-protonated heteroarene, forming an intermediate oxidized by O_2_, followed by proton loss to yield the aromatic product. Control experiments confirmed each component was essential, as removing any stopped the reaction (Scheme 89).^117^

**Scheme 90:**
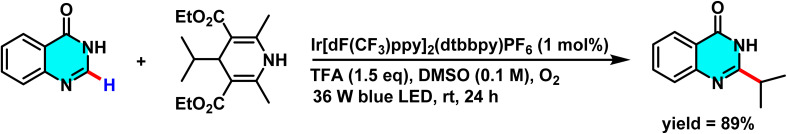
Iridium-photocatalyzed C–H alkylation of pyrimidine using 4-alkyl-1,4-dihydropyridines as radical precursors under blue LED irradiation.

In 2020, a photochemical strategy was developed. 4-Alkyl-1,4-dihydropyridines acted as alkyl radical precursors under (470) nm blue LED irradiation with the photocatalyst Ir[dF(CF_3_)ppy]_2_(dtbbpy)PF_6_, enabling pyrimidine alkylation in the presence of O_2_ and TFA. This reaction, performed in DMSO, proceeds in moderate to high yields and displays notable selectivity (Scheme 90). A key advantage of this approach lies in its utilization of molecular oxygen as an oxidant instead of harsh chemical alternatives, rendering it environmentally sustainable.

The mechanism started with photoexcitation of the Ir(iii) photocatalyst under visible light, undergoing SET to O_2_ to form a superoxide radical (O^2−^˙) and Ir(iv). Simultaneously, 4-alkyl-1,4-dihydropyridine oxidized by O^2−^˙ or TFA produced an alkyl radical. This radical added to the pyrimidine ring, forming a transient intermediate oxidized by O_2_ or Ir(iv), followed by proton loss to yield the product. Radical trapping with TEMPO and BHT confirmed the radical pathway.^118^

**Scheme 91:**
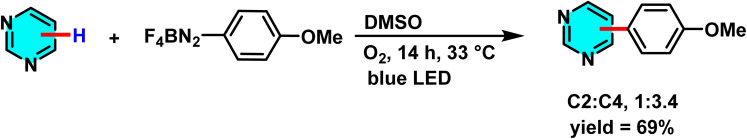
Photocatalyst-free, visible-light-induced C–H arylation of pyrimidine using aryl diazonium salts.

In 2020, Silva and co-workers reported a photocatalyst-free C–H arylation of diazines, including pyrimidine, using aryldiazonium salts under blue LED irradiation (120 W, 33 °C, 14 h) in DMSO with 15-fold excess diazine. Pyrimidine yielded C-2/C-4 arylated products (1 : 3.4 ratio, 69% total). Aryl diazonium salts with electron-donating or -withdrawing groups were tolerated, with C-4 substitution favored due to reduced steric hindrance at C-2 (Scheme 91).

**Scheme 92:**
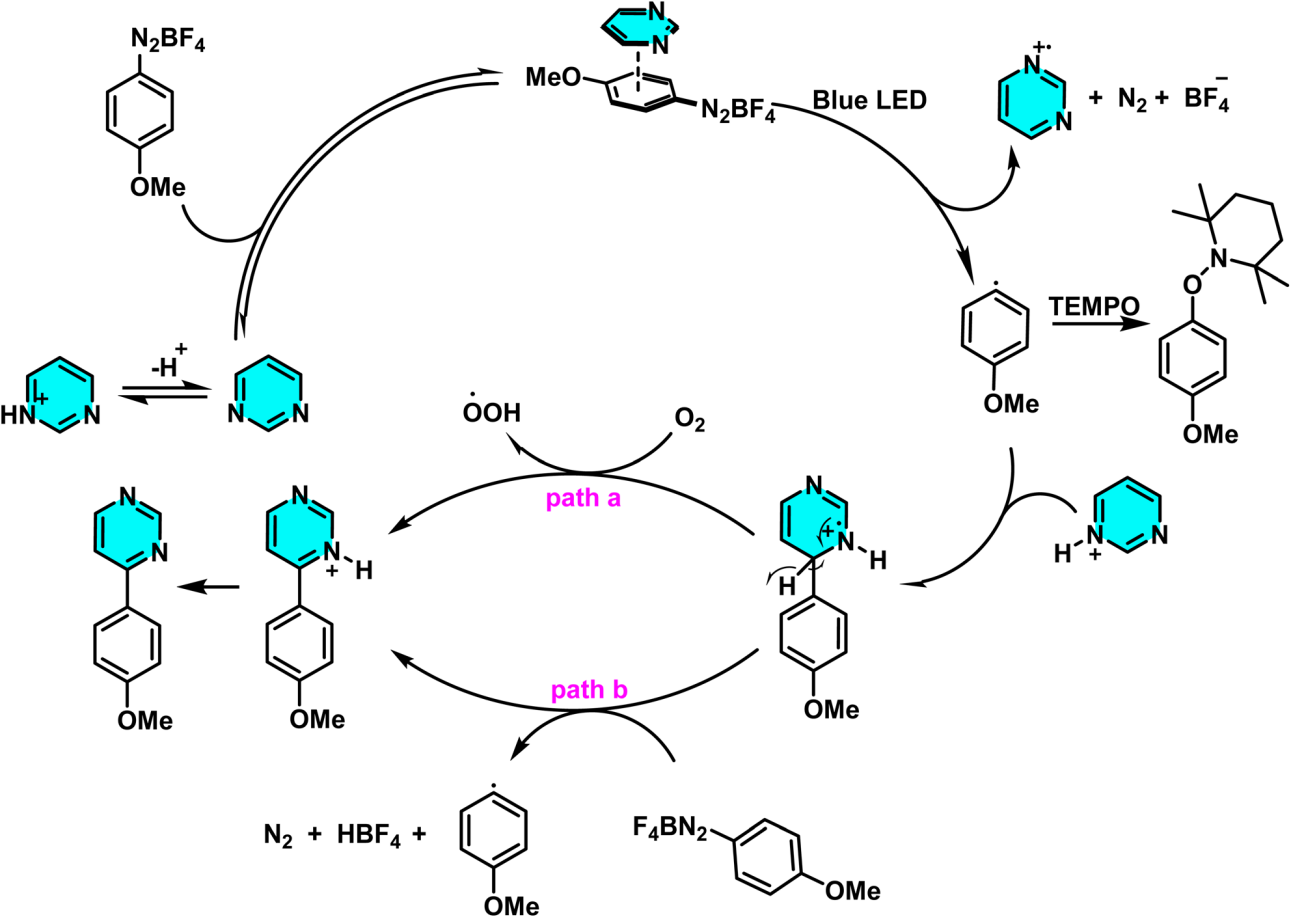
Mechanistic pathway illustrating the visible-light-driven direct arylation of pyrimidine C–H bonds *via* aryl diazonium salts under catalyst-free conditions.

The mechanism depended on forming an EDA complex between pyrimidine and the diazonium salt, activated by visible light to induce SET and generate an aryl radical. This radical attacked the pyrimidine C–H bond and oxidized by O_2_ or another diazonium molecule to yield the product. UV-Vis spectroscopy in DMSO showed a charge-transfer band at 428 nm, confirming the EDA complex, while no band appeared in water, linking to lower yields (Scheme 92).^119^

**Scheme 93:**
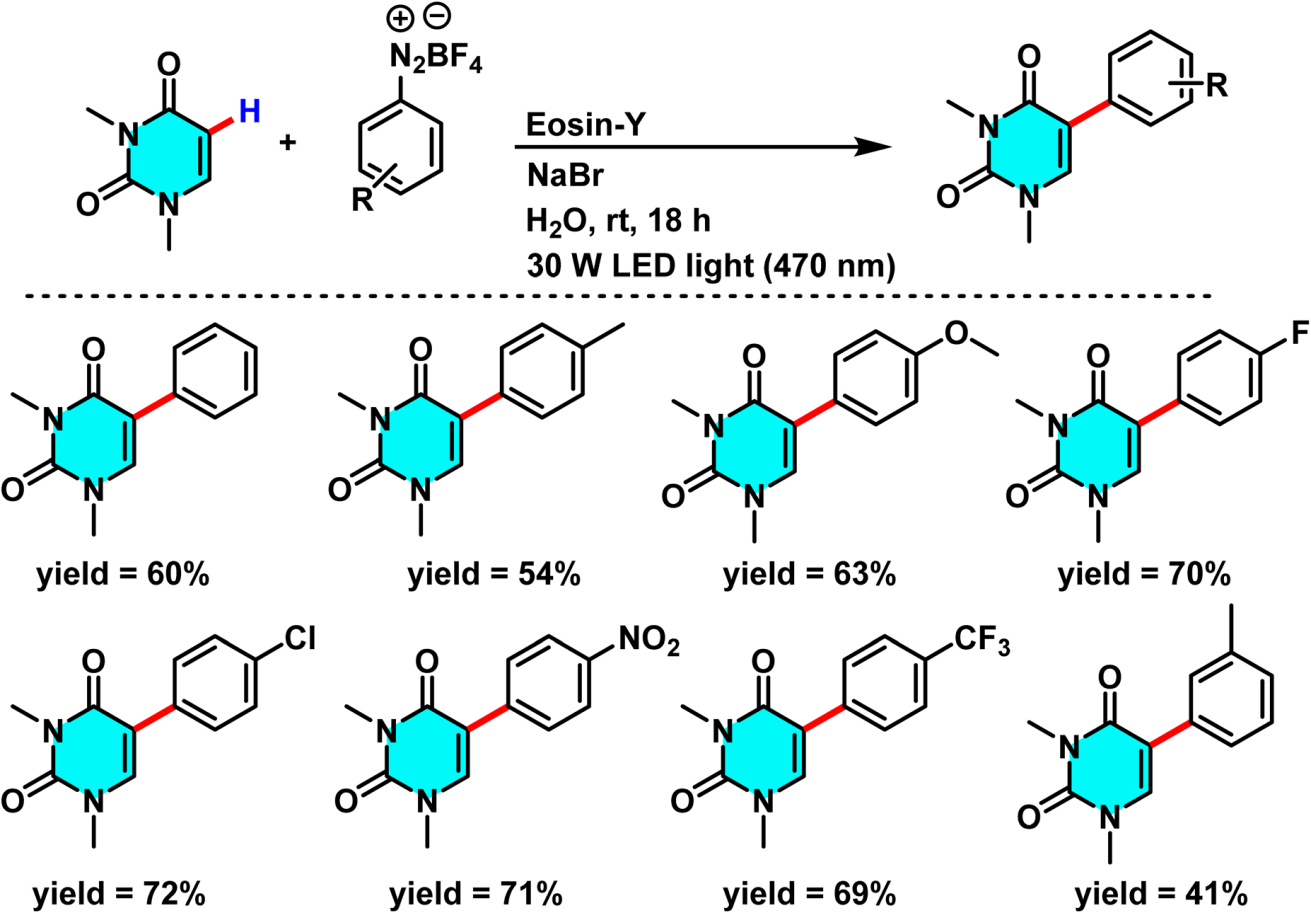
Photocatalytic C(5)-arylation of 1,3-dimethyluracil under 30 W blue LED irradiation.

**Scheme 94:**
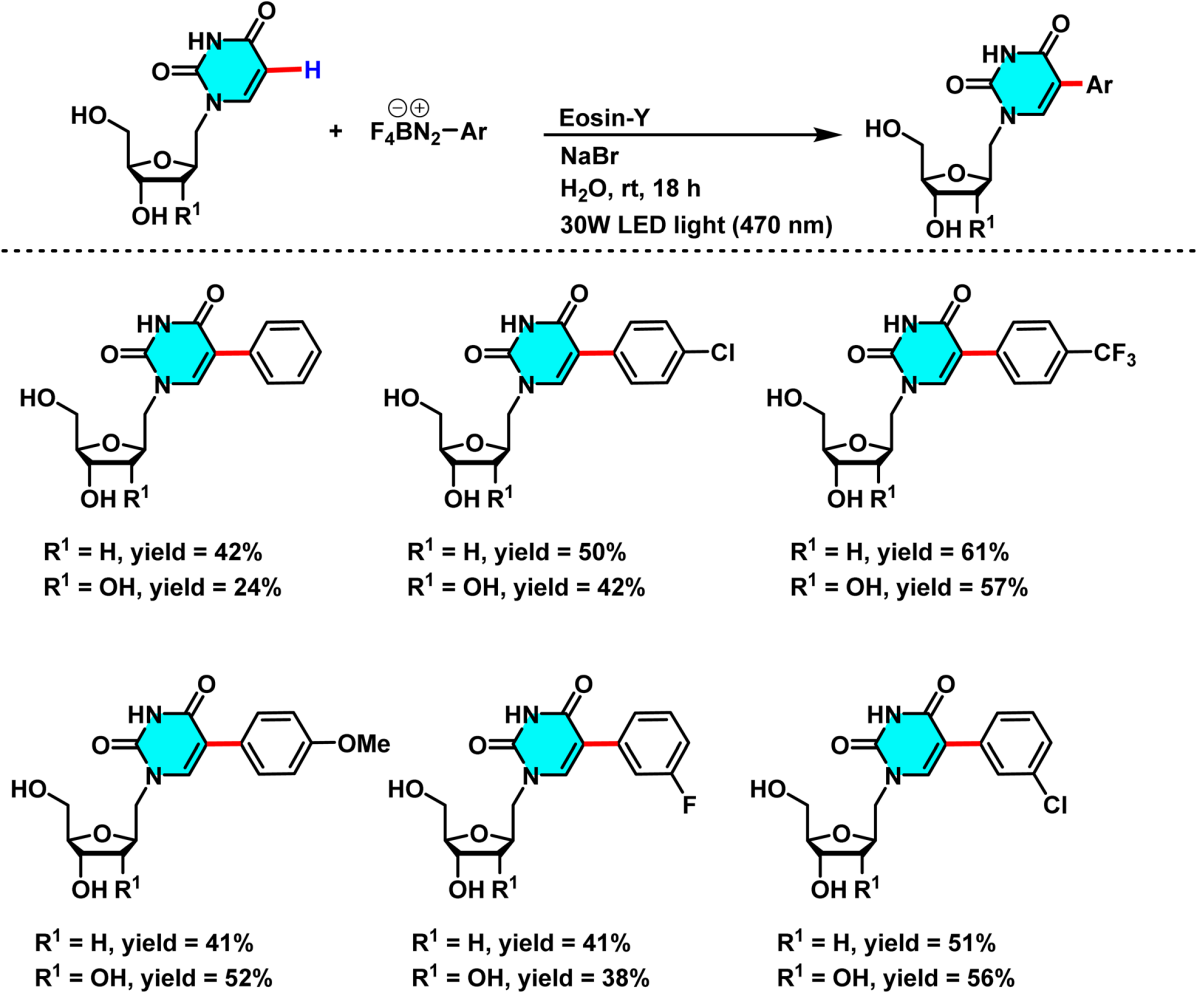
Photocatalytic C(5)-arylation of uridine and deoxyuridine derivatives using blue LED light (30 W) under metal-free conditions.

In addition to pyrimidine activation, studies on the C–H functionalization of uracils were conducted by Liu and co-workers in 2021. This efficient methodology enabled the direct C(5)-arylation of 1,3-dimethyluracil and related derivatives through a visible-light-driven photochemical protocol. The reaction was carried out using aryl diazonium salts and 2.5 mol% eosin-Y as the organic photocatalyst, under blue LED irradiation (30 W) in pure water at ambient temperature for 18 hours, providing the desired products in moderate to excellent yields (Scheme 93). The methodology was subsequently extended to the arylation of unprotected nucleosides, including uridine and deoxyuridine, which proceeded efficiently under the same mild, metal-free, and aqueous conditions, affording the corresponding C(5)-arylated products in good yields (Scheme 94).

**Scheme 95:**
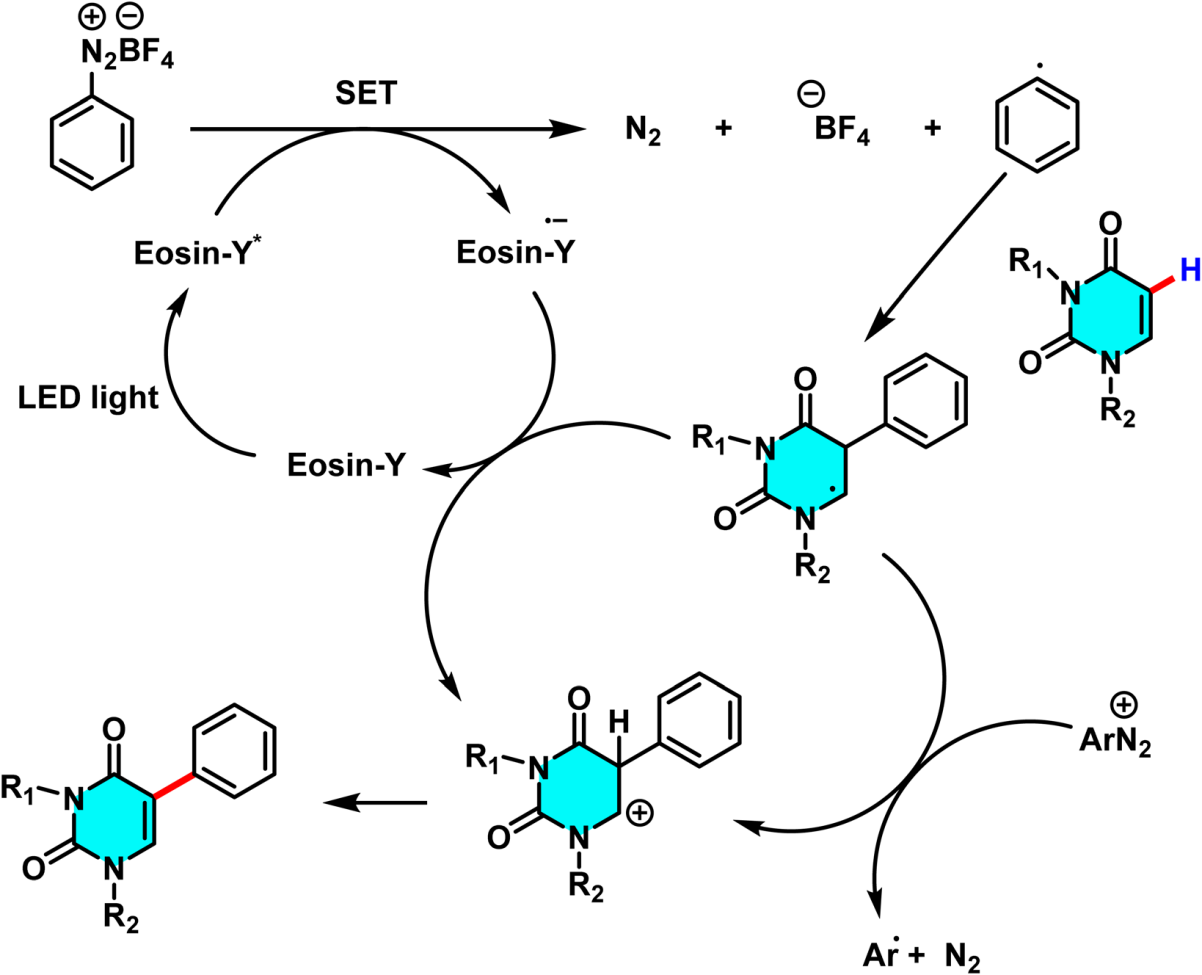
Photocatalytic mechanism suggested for the C–H functionalization of uracil derivatives.

The mechanism relied on a radical pathway, with visible light exciting eosin-Y to trigger electron transfer to the diazonium salt, releasing N_2_ and forming an aryl radical. This radical added to the C-5 position of uracil, followed by oxidation to produce the product. Control experiments with TEMPO trapping and light on–off tests confirmed the photocatalyst's role and radical nature (Scheme 95).^120^

**Scheme 96:**
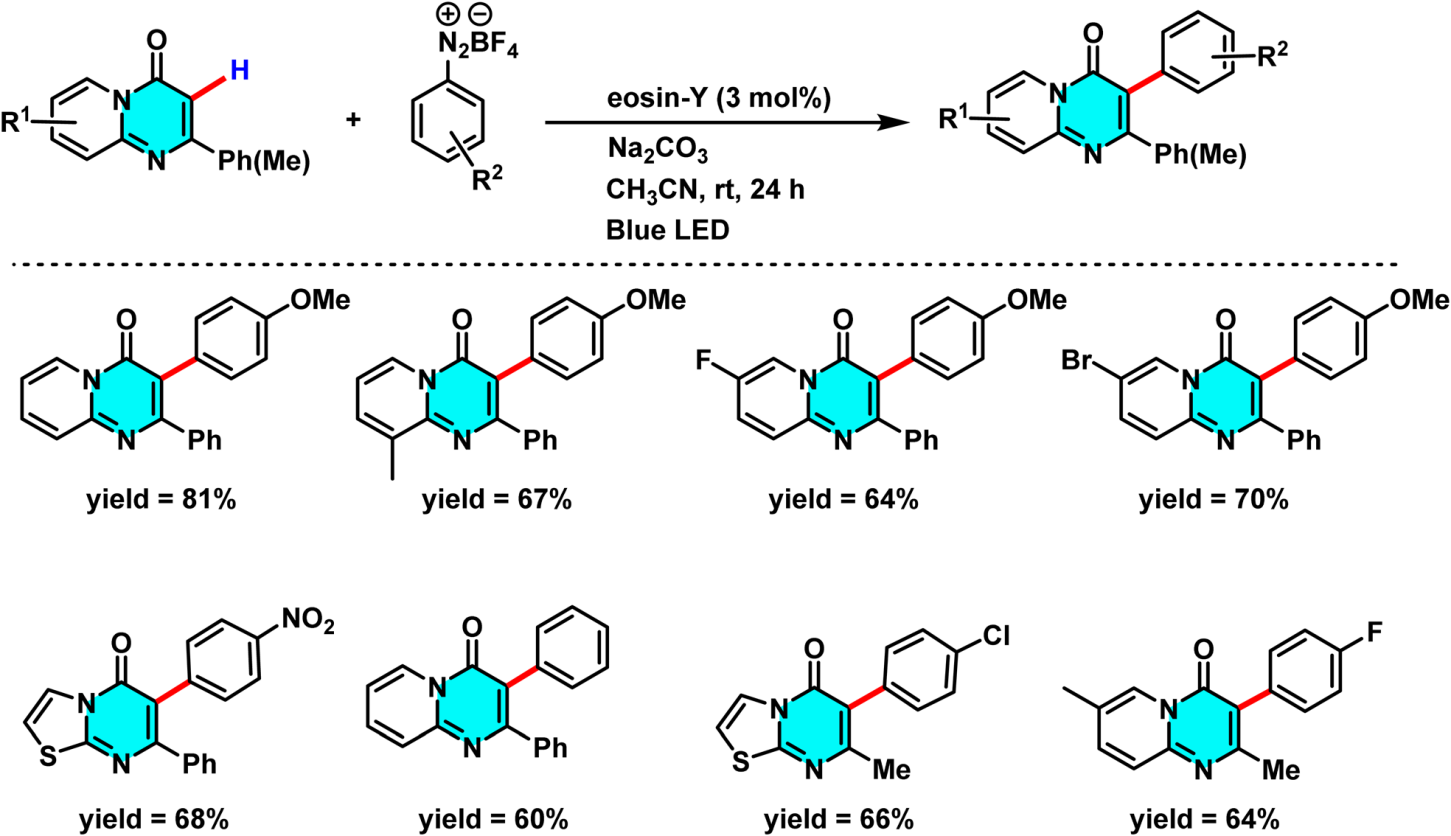
Eosin Y-catalyzed C(3)–H arylation of pyrido[2,1-*a*]pyrimidin-4-ones with aryl diazonium salts under visible-light irradiation, providing structurally diverse products in moderate to good yields under mild, metal-free conditions.

In 2023, a metal-free protocol was developed for the C-3 arylation of pyrido[2,1-*a*]pyrimidin-4-ones, utilizing visible light (blue LED) and eosin-Y as the photocatalyst. Eosin-Y, an organic dye from the xanthene family with the molecular formula C_20_H_6_Br_4_Na_2_O_5_, absorbs visible light in the 515–540 nm range, functioning as a photocatalyst to generate radicals. This water-soluble, red-orange powder is widely favored in green chemistry applications due to its stability and cost-effectiveness. The method involves the reaction of aryl diazonium tetrafluoroborate salts with pyrimidines in the presence of Na_2_CO_3_ and acetonitrile under mild conditions (room temperature, 24 hours) to yield the products with satisfactory efficiency (Scheme 96).

**Scheme 97:**
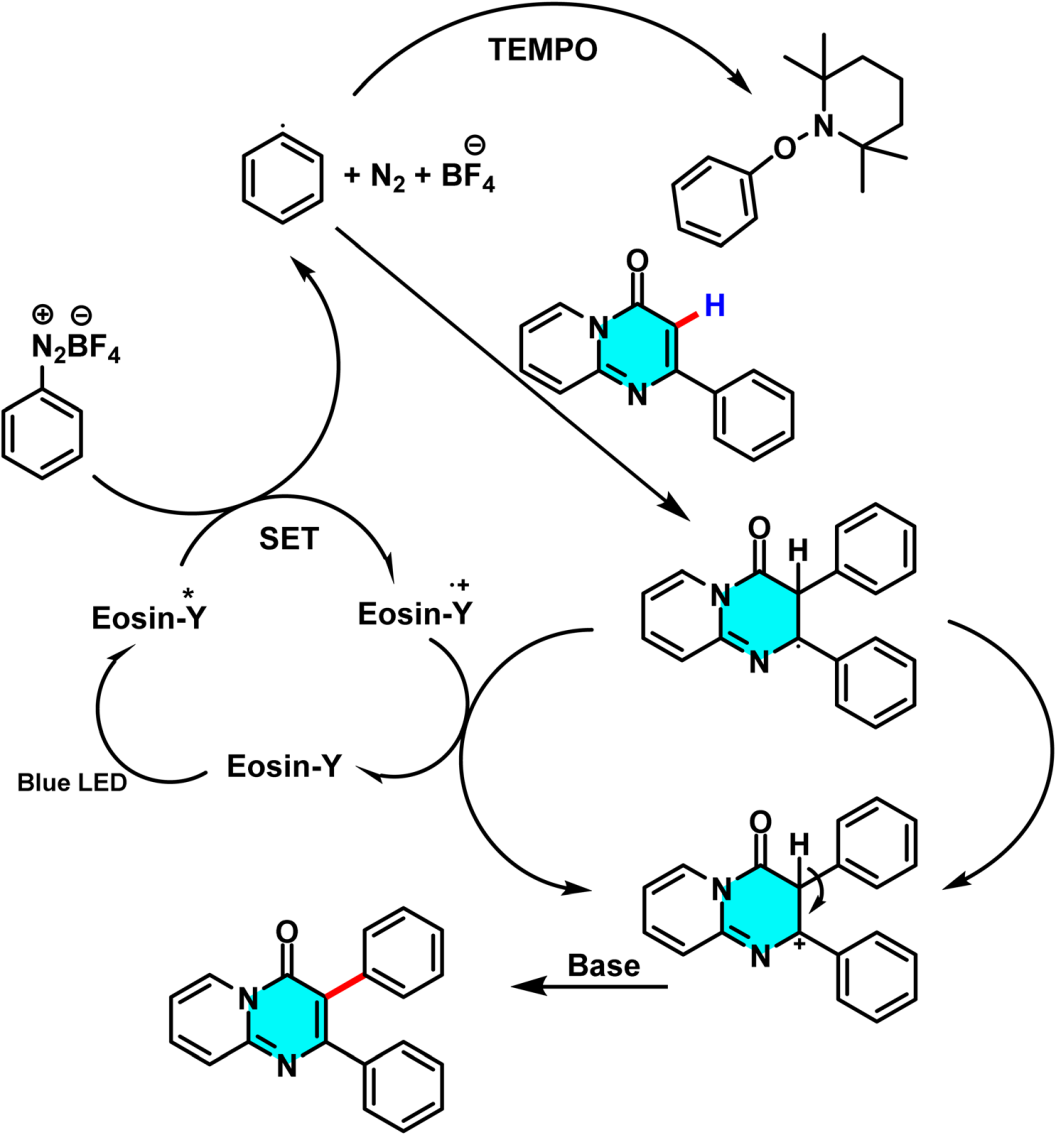
Proposed mechanism for the metal-free C(3)–H arylation of pyrido[2,1-*a*]pyrimidin-4-ones *via* visible-light-induced generation of aryl radicals from diazonium salts.

The mechanism of this reaction proceeds *via* a radical pathway. Visible light initially excites eosin-Y to its photoactivated state, which then transfers an electron to the aryl diazonium salt, generating an aryl radical that subsequently adds to the C-3 position of the pyrimidine scaffold. The resulting radical intermediate is oxidized by molecular oxygen (O_2_), leading to the formation of the aromatic product. The radical nature of this process was substantiated through control experiments, including inhibition with TEMPO and on–off light-switching studies (Scheme 97).^121^

**Scheme 98:**
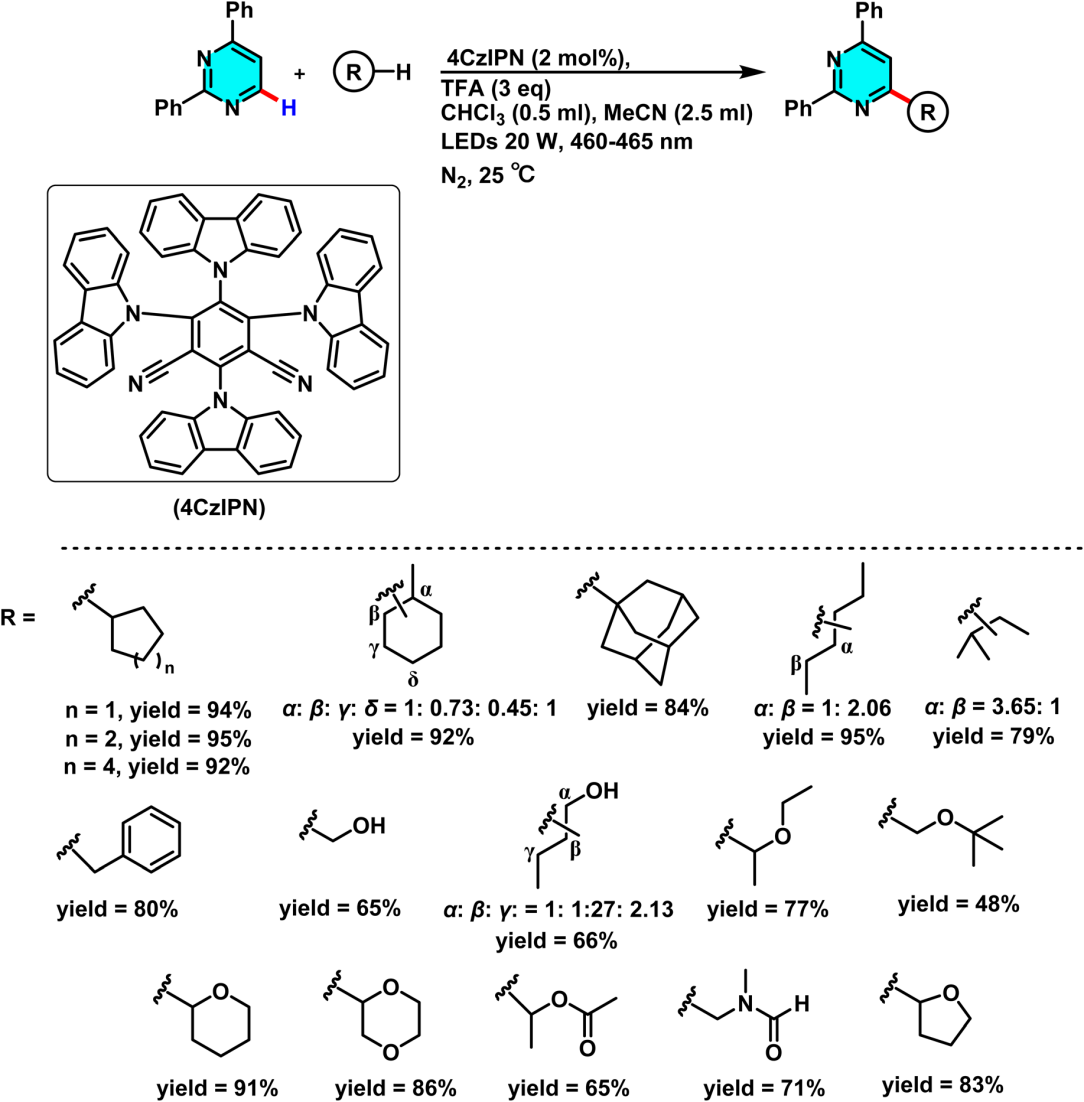
Photocatalytic C–H alkylation of pyrimidines using CHCl_3_ and 4CzIPN under blue LED light.

In the most recent development in this field, Zhang and co-workers (2025) reported an innovative and environmentally friendly protocol for the Minisci alkylation of pyrimidines, utilizing chloroform (CHCl_3_) as both the radical source and oxidant under visible-light irradiation. The reaction proceeds under blue LED light, without the need for any transition-metal-catalysts or strong oxidants, with chloroform serving a dual role as both solvent and precursor for alkyl radicals (Scheme 98). The mechanism involved visible-light irradiation inducing homolytic cleavage of chloroform to form trichloromethyl radicals (˙CCl_3_). These radicals abstracted a hydrogen from an alkane or chloroform, producing an alkyl radical (R˙). The alkyl radical added to the pyrimidine ring, forming a radical intermediate oxidized by chloroform or oxygen to yield the alkylated product. Control experiments with TEMPO confirmed the radical process.^122^

### 4.1 Critical analysis for photochemical C–H activation

Photochemical C–H activation in pyrimidines, mediated by visible-light photocatalysts such as eosin-Y and iridium complexes, facilitates selective radical-driven arylation and alkylation reactions under mild and environmentally friendly conditions. Recent protocols, including Zhang's 2025 chloroform-based alkylation, demonstrate operational simplicity and enhanced sustainability. These photochemical methods enable unique reactivities, such as remote functionalization, thereby aligning well with the principles of green chemistry.

Nonetheless, challenges remain, including the high cost and limited availability of iridium-based photocatalysts, variable efficiency with electron-deficient pyrimidine substrates, and scalability concerns stemming from limited light penetration in larger-scale reactors. To address these issues, the development of cost-effective, recyclable photocatalysts and advanced photoreactor designs is essential to facilitate industrial-scale applications.

Moreover, integrating photochemical approaches with either metal-free or metal-catalyzed strategies holds great promise to broaden reaction scope and improve overall versatility. Such synergistic methodologies could establish photochemistry as a foundational tool for sustainable and efficient synthesis of pyrimidine derivatives in pharmaceutical and materials science.

## 5 Future perspectives and challenges

Direct C–H activation in pyrimidines and related heteroarenes holds immense promise for pharmaceutical and material synthesis, yet significant challenges remain. Scalability is a primary concern: transition-metal-catalyzed methods, such as those using palladium or nickel, face limitations due to high catalyst costs and environmental concerns from metal waste. Metal-free Minisci-type reactions, while sustainable, suffer from lower regioselectivity and reliance on stoichiometric reagents, limiting atom economy. Photochemical approaches, exemplified by Zhang's 2025 chloroform-based protocol, offer green alternatives but are hindered by light penetration issues and the expense of iridium-based photocatalysts. Overcoming these challenges requires innovative strategies. For metal-catalyzed methods, developing earth-abundant catalysts like nickel or copper could enhance cost-effectiveness and sustainability. Metal-free approaches would benefit from catalytic radical initiators to improve selectivity and efficiency, potentially integrating with photochemical strategies to address regioselectivity. Photochemical methods need affordable, recyclable photocatalysts, such as eosin-Y, and advanced reactor designs to enable large-scale applications. Computational modeling to predict reaction pathways and optimize catalyst–substrate interactions could unify these approaches, expanding substrate scope and reaction efficiency. Furthermore, synergistic combinations of metal-free, metal-catalyzed, and photochemical methods could unlock novel pathways for synthesizing complex pyrimidine derivatives, advancing applications in therapeutics and materials. These advancements will be pivotal in establishing C–H activation as a cornerstone of sustainable heterocyclic chemistry.

## 6 Conclusion

Direct C–H activation in pyrimidines and related heteroarenes has transformed organic synthesis, enabling efficient production of bioactive molecules and advanced materials. This review underscores the strengths of transition-metal-catalyzed, metal-free, and photochemical approaches, achieving high selectivity and sustainable efficiency. Methods using palladium, nickel, Minisci-type reactions, and photocatalysts like eosin-Y have driven significant progress in pharmaceutical and material applications. Despite challenges, such as scalability and cost, these strategies offer promising pathways for innovation. Continued advancements, as discussed in the preceding section, will further enhance their impact, solidifying C–H activation as a cornerstone of sustainable heterocyclic chemistry for future therapeutic and material developments.

## Conflicts of interest

The authors declare that they have no known competing financial interests or personal relationships that could have appeared to influence the work reported in this paper.

## Data Availability

No data was used for the research described in the article.
